# Assessing non-inferiority for binary matched-pairs data with missing values: a powerful and flexible GEE approach based on the risk difference

**DOI:** 10.1186/s12874-025-02497-2

**Published:** 2025-02-27

**Authors:** Johannes Hengelbrock, Frank Konietschke, Juliane Herm, Heinrich Audebert, Annette Aigner

**Affiliations:** 1 Institute of Biometry and Clinical Epidemiology, Charité – Universitätsmedizin, Freie Universität Berlin and Humboldt-Universität Zu Berlin, Charitéplatz 1, 10117 Berlin, Germany; 2 Department of Neurology and Center for Stroke Research Berlin, Freie Universität Berlin and Humboldt-Universität Zu Berlin, Campus Benjamin Franklin, Charité – Universitätsmedizin, Berlin, Germany; 3 Klinik Für Neurologie, Martin Gropius Krankenhaus, Eberswalde, Germany; 4 Center for Stroke Research Berlin, Charité – Universitätsmedizin, Freie Universität Berlin and Humboldt-Universität Zu Berlin, Berlin, Germany

**Keywords:** Risk difference, Confidence interval, GEE, Binary matched pairs, Non-inferiority

## Abstract

**Background:**

Clinical studies often aim to test the non-inferiority of a treatment compared to an alternative intervention with binary matched-pairs data. These studies are often planned with methods for completely observed pairs only. However, if missingness is more frequent than expected or is anticipated in the planning phase, methods are needed that allow the inclusion of partially observed pairs to improve statistical power.

**Methods:**

We propose a flexible generalized estimating equations (GEE) approach to estimate confidence intervals for the risk difference, which accommodates partially observed pairs. Using simulated data, we compare this approach to alternative methods for completely observed pairs only and to those that also include pairs with missing observations. Additionally, we reconsider the study sample size calculation by applying these methods to a study with binary matched-pairs setting.

**Results:**

In moderate to large sample sizes, the proposed GEE approach performs similarly to alternative methods for completely observed pairs only. It even results in a higher power and narrower interval widths in scenarios with missing data and where missingness follows a missing (completely) at random (MCAR / MAR) mechanism. The GEE approach is also non-inferior to alternative methods, such as multiple imputation or confidence intervals explicitly developed for missing data settings. Reconsidering the sample size calculation for an observational study, our proposed approach leads to a considerably smaller sample size than the alternative methods.

**Conclusion:**

Our results indicate that the proposed GEE approach is a powerful alternative to existing methods and can be used for testing non-inferiority, even if the initial sample size calculation was based on a different statistical method. Furthermore, it increases the analytical flexibility by allowing the inclusion of additional covariates, in contrast to other methods.

**Supplementary Information:**

The online version contains supplementary material available at 10.1186/s12874-025-02497-2.

## Background

Studies with binary matched pairs often test the difference in the risk between the outcome of two alternative interventions. Binary matched-pairs data can typically be encountered in trials with binary endpoints such as before and after (e.g., [1–3]) or during two types of interventions [4]. The present study was motivated by a comparable study in patients hospitalized for acute ischemic stroke or transient ischemic attack, the VISIT STROKE study [5]. In this study, each patient receives both a telemedicine-based ward-round (novel approach) and an on-site ward-round (standard of care) by neurologists on the same day. The VISIT STROKE study aims to test the non-inferiority of the telemedicine-based assessments compared to the on-site ward-rounds regarding the quality of medical assessments and recommendations. The correctness of the medical assessment and recommendation is defined as a binary endpoint (correct vs. incorrect), and the non-inferiority margin is defined on the risk difference scale.

For the non-inferiority test of a risk difference for binary matched-pairs data, Nam [6] proposed a test statistic based on the score function, which has emerged as a standard and is frequently used for sample size calculation. Tango [7] developed confidence intervals (CIs) that are compatible with the test by Nam [8]. However, a frequent challenge with the analysis of binary matched-pairs data is that for some patients only one outcome observation is available. The methods Nam and Tango developed are based on information from completely observed pairs only and cannot incorporate information from pairs with partially observed data. Therefore, after the initial publications by Nam [6] and Tango [7], several different approaches have been suggested to allow the incorporation of data from partially observed pairs. So-called *hybrid* methods combine estimates based on completely and incompletely observed pairs and have shown to be an improvement upon methods solely based on completely observed pairs [9, 10]. However, these methods do not allow for the adjustment of additional covariates' impact. Previously, approaches based on generalized estimating equations (GEE) have been suggested as an alternative to McNemar’s test for testing superiority, with the potential to include additional covariates [11]. The GEE approach has also been suggested for estimating CIs for the risk difference [12]. However, to our knowledge, the GEE approach has not been compared to the standard approaches by Nam and Tango in the case of completely observed pairs or to improvements in the form of hybrid CIs [10]. Since the GEE approach allows for both the inclusion of incompletely observed pairs as well as additional covariates, it potentially increases the statistical power (in the case of missing data) as well as the flexibility of the statistical analysis (by incorporating additional covariates). This is why the comparison with standard approaches is of interest for the case of binary matched-pairs data in a non-inferiority setting.

We fill this gap with a simulation-based comparison of different methods in the presence and absence of different missing data patterns. These methods include the original CIs by Tango [7], a multiple imputation (MI) approach with Wald-type intervals similar to the one by Tango, the hybrid CIs suggested by Tang et al. [10], as well as CIs based on a GEE approach [13]. We study the power, coverage probabilities, and widths of the different CIs for the risk difference in settings without missing values, with values missing completely at random (MCAR), and values missing at random (MAR) [14].

## Methods

### Binary matched-pairs setting

In the following, we assume that all patients $c, c=1, ..., n,$ undergo two different interventions (e.g., treatments or assessments), $k, \,k=1, 2$. However, only one of the two outcomes was observed in some patients. We denote ${Y}_{c,k}$ a vector of binary outcomes,${Y}_{c,k}=\{f({\lambda }_{c,1},{\widetilde{Y}}_{c,1}), f({\lambda }_{c,2},{\widetilde{Y}}_{c,2})\}$, with ${\widetilde{Y}}_{c,k}$ as binary outcome of treatment $k$ and patient $c$ which was or would have been observed and ${\lambda }_{c,k}$ as indicator variable denoting whether an outcome has been observed (1) or is missing (0). Missing values are denoted by $x$, that is:

$$f\left({\lambda }_{c,k},{\widetilde{Y}}_{c,k}\right)={Y}_{c,k}=\left\{\begin{array}{cc}{\widetilde{Y}}_{c,k}& , \text{if}\;{\lambda }_{c,k} = 1\\ x& , \text{otherwise}\end{array}\right.$$

For our setting and similar to Tang et al. [10], we assume that the missing mechanism is MAR, which means that the probability of missingness $P({\lambda }_{c,k}=1)$ is independent of the outcome but may be dependent on the intervention [14], i.e., $P({\lambda }_{c,k}|k, {\widetilde{Y}}_{c,k})=P({\lambda }_{c,k}|k)$. We also investigate scenarios with the stronger assumption of MCAR, that is, $P({\lambda }_{c,k}|k, {\widetilde{Y}}_{c,k})=P({\lambda }_{c,k})$.

Furthermore, let ${y}_{k=1, c}$ and ${y}_{k=2, c}$ denote the observed realizations of ${Y}_{c,k}$ for a single patient, with $0$ indicating a failure (or non-event), $1$ success (or event), and $x$ a missing observation. In the following, we simplify the notation of ${y}_{k=1, c}$ and ${y}_{k=2, c}$ to ${y}_{1}$ and ${y}_{2}$, respectively. Based on these two observations, ${n}_{\text{0,0}}$ denotes the number of patients for whom both observed outcomes are 0 (that is, ${y}_{1}=0$ and ${y}_{2}=0$), ${n}_{\text{1,0}}$ the number of patients with ${y}_{1}=1$ and ${y}_{2}=0$, and ${n}_{\text{0,1}}$ and ${n}_{\text{1,1}}$ defined in the same fashion. Additionally, we denote ${n}_{+,0}$ as patients with non-missing outcome in ${y}_{1}$ and ${y}_{2}=0$ (and similarly so for ${n}_{+,1}, {n}_{0,+}$, and ${n}_{1,+}$). The observed frequencies can then be displayed in a 3 × 3 table (Table 1).

**Table 1:** Frequencies for the binary matched-pairs outcomes (${y}_{1} \text{ and}$ ${y}_{2}$)

	${y}_{2}=0$	${y}_{2}=1$	${y}_{2}=x$	Total
${y}_{1}=0$	${n}_{\text{0,0}}$	${n}_{\text{0,1}}$	${n}_{0,x}$	${n}_{0,+}+{n}_{0,x}$
${y}_{1}=1$	${n}_{\text{1,0}}$	${n}_{\text{1,1}}$	${n}_{1,x}$	${n}_{1,+}+{n}_{1,x}$
${y}_{1}=x$	${n}_{x,0}$	${n}_{x,1}$	0	${n}_{x,+}$
Total	${n}_{+,0}+{n}_{x,0}$	${n}_{+,1}+{n}_{x,1}$	${n}_{+,x}$	$n$

Note that patients for whom both observations are missing are removed from the analysis, and thus, each included patient contributes at least one observation

The probabilities corresponding to the cells in Table 1 are defined as follows: ${p}_{i,j}$ is the conditional probability of a patient having observed outcome ${y}_{1}=i$ and ${y}_{2}=j$, with both observations being non-missing (i.e., $i, j = 0, 1$). Similarly, ${p}_{0,+}$ denotes the conditional probability of observing ${y}_{1}=0$ with ${y}_{2}$ being non-missing, and equivalent for ${p}_{+,0}$. In the same fashion, ${p}_{x,+}$ is the probability of ${y}_{1}=x$ conditional on ${y}_{2}$ being non-missing (and so forth). Furthermore, let ${p}_{1,}$ and ${p}_{,1}$ denote the unconditional probabilities of observing ${y}_{1}=1$ and ${y}_{2}=1$, respectively.

We are interested in whether the assessments of the alternative intervention are non-inferior to those of the standard intervention, with a non-inferiority margin $\delta$. The risk difference $\uptheta ={p}_{1,}-{p}_{,1}$ provides a measure of equivalence. The assumption of MAR implies that while the probabilities of missingness differ between the two interventions, the risk difference is the same for all patients (i.e., those with missings and those without). Under the assumption of MAR and from Table 1, two estimates of the risk difference become obvious: one based on complete observations only as ${n}_{1,+}/{n}_{+,+}-{n}_{+,1}/{n}_{+,+}$, and a second based on all patients as ${(n}_{1,+}+{n}_{1,x})/({n}_{+,+}+{n}_{+,x})-{(n}_{+,1}+{n}_{x,1})/({n}_{+,+}+{n}_{x,+})$ (see also [10]). Note that these are valid estimates only under MAR and that the estimate based on complete observations is biased if data is missing not at random.

In the following, we first review an established method for obtaining CIs based on patients with complete observations only. Afterward, we extend it to methods which allow the inclusion of patients with only partially observed data.

### Confidence intervals based on completely observed pairs only

Nam [6] suggested a test based on the score function method for testing the null hypothesis

1 $${H}_{0}:\uptheta \le -\delta \text{ versus }{H}_{1}:\uptheta>-\delta$$

based on complete data, which has emerged as a standard for the sample size calculation for the binary matched-pairs setting [15]. The test by Nam can be accompanied by the CIs derived by Tango [7], which are based on the identical score function and thus lead to intervals consistent with the hypothesis test. The test statistic and CIs are derived under the assumption that the observations of different patients are independent and identically distributed and are based on patients with completely observed outcomes. Here and in the following, we denote ${\widehat{\theta }}_{cc}$ the estimate based on patients with complete observations, that is, ${\widehat{\theta }}_{cc}={\widehat{p}}_{1,+}-{\widehat{p}}_{+,1}={\widehat{p}}_{\text{1,0}}-{\widehat{p}}_{\text{0,1}}$. Following the previous section, the estimate based on patients with complete observations has mean $\uptheta$ and variance ${\upsigma }^{2}=\left[\left({p}_{\text{0,1}}+{p}_{\text{1,0}}\right)-{{\theta }_{cc}}^{2}\right]/n$ (for details, see [8]). A simple approach to estimate the variance involves replacing the parameters with their sample estimates, the unrestricted maximum likelihood (ML) estimator. As an alternative, Nam [6] suggested an approach based on the restricted ML estimation (RMLE). Under the null hypothesis in [1], the RMLE of ${\upsigma }^{2}$ is ${\widetilde{\upsigma }}_{l}^{2}=\left[\left({\widetilde{\text{p}}}_{\text{1,01}}+{\widetilde{\text{p}}}_{\text{1,10}}\right)-{\updelta }^{2}\right]/n$, where ${\widetilde{\text{p}}}_{\text{1,01}}$ and ${\widetilde{\text{p}}}_{\text{1,10}}$ are the RMLEs evaluated at the boundary, $\uptheta =-\delta$, which is ${\widetilde{\text{p}}}_{\text{1,01}}=[-{\widetilde{a}}_{1}+{({\widetilde{a}}_{1}^{2}-8{\widetilde{b}}_{1})}^{1/2}]/4$ and ${\widetilde{\text{p}}}_{\text{1,10}}={\widetilde{\text{p}}}_{\text{1,01}}-\updelta$, with ${\widetilde{a}}_{1}=-{\widehat{\theta }}_{cc}(1-\updelta )-2({\widehat{p}}_{\text{0,1}}+\updelta )$ and ${\widetilde{b}}_{1}=$ $\updelta (1+\updelta ){\widehat{p}}_{\text{0,1}}$. Note that for the case $\delta =0$, the test by Nam reduces to McNemar’s test statistic [6].

Following that, a REML-based $100\cdot \left(1-2{\alpha}\right) \%$ CI (denoted as $\left({\widehat{\theta }}_{cc, l},{\widehat{\theta }}_{cc, u}\right)$) based on patients with complete observations can be obtained by solving

2 $${\widehat{\theta }}_{cc, l}=inf\left\{{\uptheta }_{0}:\frac{\left({\widehat{\theta }}_{cc}-{\uptheta }_{0}\right)}{{\widetilde{\upsigma }}_{{\uptheta }_{0}}}<{z}_{{\alpha }}\right\}\text{and }{\widehat{\theta }}_{cc,u}= sup\left\{{\theta }_{0}:\frac{\left({\widehat{\theta }}_{cc}-{\theta }_{0}\right)}{{\widetilde{\upsigma }}_{{\uptheta }_{0}}}>{z}_{\alpha }\right\}$$

with ${z}_{\alpha }$ as the upper $100\cdot {\alpha \%}$ percentile of the standard normal distribution [8].

### Confidence intervals incorporating information from incompletely observed pairs

In the following, several approaches are reviewed for incorporating information from observations with missing values.

### Wald-type confidence intervals based on the score function with multiple imputation

CIs based on the score method described above are based solely on observations with discordant pairs (${n}_{\text{1,0}}$ and ${n}_{\text{0,1}}$) and ignore patients with missing observations. One way to include data from patients with incomplete information is by imputing their missing values, e.g., via multiple imputation (MI), and then applying the pooling methods on the results suggested by Rubin [16]. However, since analytical solutions do not exist (yet), using the above- introduced score-based CIs becomes challenging in the setting of MI. Specifically, the numerical solutions to Eq. (2) prevent us from fixing the mean and variance estimates to certain values, e.g., to those resulting from applying Rubin’s rules [16]. Therefore, we use Wald-type CIs for the MI approach with the above-described unrestricted ML estimate of ${\upsigma }^{2}$, ${\widehat{\sigma }}^{2}=\left[\left({\widehat{p}}_{\text{0,1}}+{\widehat{p}}_{\text{1,0}}\right)-{{\widehat{\theta }}_{cc}}^{2}\right]/n$. We follow the standard procedure implemented in the mice package in R [17], which is to fit logistic regression models for the two outcome variables based on the completely observed pairs:

$$\text{logit}\left({Y}_{c,k=1}|{\lambda }_{c,k=1}={\lambda }_{c,k=2}=1\right)={\upbeta }_{0}+{\upbeta }_{1}{y}_{c,k=2}$$

and

$$\text{logit}\left({Y}_{c,k=2}|{\lambda }_{c,k=1}={\lambda }_{c,k=2}=1\right)={\breve{\upbeta }}_{0}+{\breve{\upbeta }}_{1}{y}_{c,k=1}.$$

From these, we obtain random draws from the normally distributed regression coefficients, compute the predicted scores based on the random draws, and dichotomize the predicted scores by comparing them to random draws from $\text{U}(\text{0,1})$. We use 10 sets of imputed data and obtain estimates of the mean and variance following Rubin [16], which we then use to calculate Wald-type CIs. In contrast to the numerical solution of the score-based interval, the mean and variance estimates that follow from Rubin [16] can simply be plugged into the analytical formula for computing the Wald-type CI. In principle, MI allows for the inclusion of additional covariates in the imputation models. However, since we did not introduce any additional covariates in our setting, we stick to the simple models defined above.

### Confidence intervals based on a hybrid approach

Since Nam [6] and Tango [7] proposed the test statistic and CI based on completely observed pairs, several suggestions have been made to extend their methods to allow for missing data. The so-called *hybrid* methods combine information on the risk difference from the completely observed pairs (${p}_{\text{1,0}}-{p}_{\text{0,1}})$ with information from pairs with missings (${p}_{1,x}-{p}_{x,1}$) (see e.g., [9]). Here, we follow the recommended approach by Tang et al. [10], but note that alternative methods exist.

Tang et al. suggest a Wald-type CI for the estimator of the risk difference based on the variance estimate

$$\widehat{Var}(\widehat{\theta })=\widehat{Var}({\widehat{p}}_{1,+})+\widehat{Var}({\widehat{p}}_{0,+})-2\widehat{\rho }\sqrt{\widehat{Var}({\widehat{p}}_{1,+}) \widehat{Var}({\widehat{p}}_{0,+})},$$

with $\widehat{\rho }$ being the estimated within-patient correlation. For the estimates $\widehat{Var}({\widehat{p}}_{1,+})$ and $\widehat{Var}({\widehat{p}}_{0,+})$, Tang et al. suggest estimating Wilson score-type CIs for both ${\widehat{p}}_{1,+}$ and ${\widehat{p}}_{0,+}$, whose lower and upper bounds are denoted as ${\widehat{l}}_{0,+}$, ${\widehat{u}}_{0,+}$, ${\widehat{l}}_{1,+}$, and ${\widehat{u}}_{1,+}$. These CIs are based on the estimates ${\breve{p}}_{1,+}={(n}_{1,+}+{n}_{1,x})/({n}_{+,+}+{n}_{+,x})$ and ${\breve{p}}_{0,+}={(n}_{0,+}+{n}_{0,x})/({n}_{+,+}+{n}_{x,+})$ which include information from all patients (also those with missing values) under the assumption of MAR. Based on the estimates of the lower and upper CI bounds and the central limit theorem, Tang et al. derive the estimates ${\widehat{Var}}_{l}({\widehat{p}}_{1,+})= {({\widehat{p}}_{1,+}-{\widehat{l}}_{1,+})}^{2}/{{z}_{\alpha /2}}^{2}$ and ${\widehat{Var}}_{u}({\widehat{p}}_{1,+})= {({\widehat{u}}_{1,+}-{\widehat{p}}_{1,+})}^{2}/{{z}_{\alpha /2}}^{2}$, (and for $\widehat{Var}({\widehat{p}}_{0,+})$ in the same fashion). Plugging these estimates into

$$L=\widehat\theta-z_{\alpha/2}\sqrt{\widehat{Var}\left(\widehat\theta\right)}\text{ and }U=\widehat\theta+z_{\alpha/2}\sqrt{\widehat{Var}(\widehat\theta)}$$

leads to the following lower and upper limits for $\theta$:

$$L =\widehat{\theta }-\sqrt{{({\widehat{p}}_{1,+}-{\widehat{l}}_{1,+})}^{2} + {({\widehat{u}}_{+,1}-{\widehat{p}}_{+,1})}^{2}-2\widehat{\rho }({\widehat{p}}_{1,+}-{\widehat{l}}_{1,+})({\widehat{u}}_{+,1}-{\widehat{p}}_{+,1})}$$

and

$$U =\widehat{\theta }+\sqrt{{({\widehat{u}}_{1,+}-{\widehat{p}}_{1,+})}^{2} + {({\widehat{p}}_{+,1}-{\widehat{l}}_{+,1})}^{2}-2\widehat{\rho }({\widehat{u}}_{1,+}-{\widehat{p}}_{1,+})({\widehat{p}}_{+,1}-{\widehat{l}}_{+,1})}$$

For details regarding the estimation of the CIs based on all observed data as well as for deriving the estimate $\widehat{\rho }$, we refer to their original paper [10].

### Confidence intervals based on a GEE approach

In the GEE approach, an estimate for the risk difference is based on the marginal probabilities ${P(Y}_{c,k}=1|k)=I(k=1)({p}_{1,+}+{p}_{1,x}) + I(k=2)({p}_{+,1}+{p}_{x,1})$, with $I(\cdot )$ being the indicator function. Note that this can be rewritten as

$${P(Y}_{c,k}=1\left|k\right)={\beta }_{0}+{\beta }_{1}I\left(k=2\right),$$

with ${\beta }_{0}=({p}_{1,+}+{p}_{1,x})$ and ${\beta }_{1}$ as the difference between the two marginal probabilities, ${\beta }_{1}=$ $({p}_{+,1}+{p}_{x,1})-({p}_{1,+}+{p}_{1,x})$, i.e. the risk difference.

For a GEE model with binary outcome ${Y}_{c,k}$, the probability ${P(Y}_{c,k}=1|k)$ is usually estimated using a link function, with the logit link being the standard choice. Since the logit link does not provide a direct estimator for the risk difference, alternative models, such as a binomial model with an identity link function, have been proposed [12]. In our setting, however, we stick to the logistic model and derive the risk difference from the directly estimated log-odds ratio, as it usually has better convergence properties than alternative models, especially in scenarios with small sample sizes [12]. In the following, we denote ${P(Y}_{c,k}=1\left|k\right)$ as ${\mu }_{c,k}$ and ${{\varvec{x}}}_{c}$ as a vector of covariates, (1, $I\left(k=2\right)$). With the logit link, the GEE model has the form

$$\text{logit}({\mu }_{c,k}) = {\varvec{\beta}}^{\prime}{{\varvec{x}}}_{c},$$

with ${\varvec{\beta}}=({\beta }_{0},{\beta }_{1})\boldsymbol{^{\prime}}$ denoting the unknown vector of coefficients and ${\beta }_{1}$ the effect of the new intervention compared to the standard one (see above).

In the following, we denote ${{\varvec{y}}}_{c}=({y}_{c,1},{y}_{c,2})^{\prime}$ as a vector of observed outcomes of patient $c$. The GEE estimator $\widehat{{\varvec{\beta}}}$ of ${\varvec{\beta}}$ can be obtained by solving

$$\bf \sum_{c=1}^{n}{{\varvec{D}}^{\prime}}_{c} {{\varvec{V}}}_{c}^{-1} ({{\varvec{y}}}_{c} - {{\varvec{\mu}}}_{c})=0,$$

where ${{\varvec{\mu}}}_{c}=({\mu }_{c,1},{\mu }_{c,2})^{\prime}$ and ${{\varvec{D}}^{\prime}}_{c}=\partial {{\varvec{\mu}}}_{c}/\partial{\varvec{\beta}}$. ${\mathbf{V}}_{c}$ is defined as

$${\mathbf{V}}_{c}=\phi {A}_{c}^{1/2}R(\alpha ){A}_{c}^{1/2},$$

with dispersion parameter $\phi$, ${A}_{c}$ as a diagonal matrix with the variance functions $V({{\varvec{\mu}}}_{c})$ on the diagonal matrix and $R(\alpha )$ as a so-called “working” covariance matrix for ${{\varvec{y}}}_{c}$ [13]. We use an exchangeable covariance structure to account for the clustering of assessments within single patients (for details, see [13]). Note that in settings with more than two observations per patient (or cluster), other specifications of the covariance matrix can lead to more efficient and/or less biased estimates [18]. In our setting with $k=2$, however, it is reasonable to assume the true correlation structure is exchangeable [19]. For the estimation of the covariance $\text{Cov}(\widehat{{\varvec{\beta}}})$**,** two approaches are available: the model-based estimate ${\text{Cov}(\widehat{{\varvec{\beta}}})}_{m}={I}_{0}^{-1}$ and the so-called *sandwich estimate* ${\text{Cov}(\widehat{{\varvec{\beta}}})}_{sw}={I}_{0}^{-1}{I}_{1}{I}_{0}^{-1}$, with

$${I}_{0}=\sum\nolimits_{c=1}^{n}\frac{\partial {{\varvec{\mu}}}_{c}^{\prime}}{\partial{\varvec{\beta}}}{{\varvec{V}}}_{c}^{-1}\frac{\partial {{\varvec{\mu}}}_{c}}{\partial{\varvec{\beta}}^{\prime}}\;\text{and}\;{I}_{1}=\sum\nolimits_{c=1}^{n}\frac{{\partial {{\varvec{\mu}}}_{c}}^{\prime}}{\partial{\varvec{\beta}}}{{\varvec{V}}}_{c}^{-1}\text{Cov}({{\varvec{y}}}_{c}){{\varvec{V}}}_{c}^{-1}\frac{\partial {{\varvec{\mu}}}_{c}}{{\partial{\varvec{\beta}}}^{\prime}}.$$

Using the sandwich estimate leads to so-called *robust CIs*, i.e., CIs that are robust to correlated observations [19]*.* Importantly, in our setting, the probability ${P(Y}_{c,k}=1\left|k\right)$ does not depend on the probability of ${Y}_{c,k}$ being missing if conditioning on $k$. Thus, formally, ${P(Y}_{c,k}=1\left|k,P({\lambda }_{c,k}\right)=1)={P(Y}_{c,k}=1\left|k,P({\lambda }_{c,k}\right)=0)={\mu }_{c,k}$ [20]. Therefore, including the observations of patients for whom only one of the two outcomes has been observed still leads to unbiased inference.

The logistic model and its GEE estimator only provide a direct effect estimate on the log-odds scale. From this, an estimate of the risk difference can be obtained as:

2 $${\widehat{\theta }}_{GEE}={\text{logit}}^{-1}({\widehat{\beta }}_{0}) - {\text{logit}}^{-1}({\widehat{\beta }}_{0}+{\widehat{\beta }}_{1}).$$

Standard errors for ${\widehat{\theta }}_{GEE}$ can be calculated based on the delta method [12] with the standard errors for ${\widehat{\beta }}_{0}$ and ${\widehat{\beta }}_{1}$ from the GEE above. Based on that, we calculate the Wald-type CIs (${\widehat{\theta } }_{GEE}-{z}_{\alpha }{{\widehat{\sigma }}^{2}}_{GEE},{\widehat{\theta } }_{GEE}+{z}_{\alpha }{{\widehat{\sigma }}^{2}}_{GEE})$.

### Alternative approaches not considered here

An alternative to the GEE approach is a model that explicitly accounts for the within-patient clustering of observations. One such approach is conditional logistic regression, which is similar to the approaches based on the score function, as inference in both cases is based only on discordant pairs. Another alternative is to explicitly account for the patient effect within the linear model:

$$\text{logit}({\mu }_{c,k})= {{\varvec{\beta}}}_{{\varvec{C}}{\varvec{o}}{\varvec{n}}{\varvec{d}}}^{\prime}{{\varvec{x}}}_{c}+{\omega }_{c},$$

where ${\omega }_{c}$ is the patient-level effect. If ${\omega }_{c}$ is estimated as a constant term for each patient (“fixed effect”), the approach is similar to a conditional logistic regression. Alternatively, ${\omega }_{c}$ can be treated as a random variable (“random effect”), usually with assumed distribution ${\tau }_{c} \sim N(0, {\tau }^{2}).$ In both cases, the coefficients ${{\varvec{\beta}}}_{{\varvec{C}}{\varvec{o}}{\varvec{n}}{\varvec{d}}}$ are conditional on the patient effect and represent the effect of changing treatment for an individual patient (that is, with a specific patient-level effect ${\omega }_{c}$). In that, they are different from the coefficients of the GEE approach, which are not subject-specific but are interpreted as population-average effects [11]. Thus, obtaining a risk difference from the random effects model via Eq. (2) with ${\omega }_{c}$ at a specific level would result in a subject-specific risk difference.

While the choice of subject-specific vs. population-averaged approaches depends on which estimand is of interest, GEE approaches have been more popular in binary matched pairs. This is mainly because estimating random effects models is computationally difficult if only two observations are available per cluster. Moreover, the binary nature of the treatment and outcome variables renders the deviation from the normality assumption of the random effects so severe that it can lead to convergence issues. Improvements based on penalized quasi-likelihood and adaptive Gaussian Hermite quadrature have been suggested, but it is somewhat unclear under which conditions good convergence and unbiased estimates can be expected [21–23]. Thus, we stick to the GEE approach for estimating CIs for the population-average risk difference in this setting and do not consider a mixed model approach. Also, Bayesian approaches to obtain credibility intervals have been proposed, e.g., [24]), which we do not consider here.

### Simulation study

We study the properties of the four described methods in different simulation scenarios, including the sample size calculation for the VISIT STROKE study as one scenario, along with several alternative settings. In detail, we generate correlated binary data for the following combinations of parameters:

1. number of patients $n, n \in \{20, 30, 40, 50, 60, 70, 80, 90, 100, 200, 300, 400, 507\}$
2. within-patient correlation $\rho , \rho \in \{0, 0.37, 0.5\}$
3. non-inferiority margin for the risk difference $\delta$ and true underlying risk difference $\uptheta$: {$\delta =0,\uptheta =0.05; \delta =0.05,\uptheta =0; \delta =0.1,\uptheta =0\}$
4. probabilities of single observations being missing (${p}_{x,+}$ and ${p}_{+,x}): \{\{0, 0\}, \{0.15, 0.15\}, \{0.25, 0.25\} \text{ and } \{0.1, 0.3\}\}$,

resulting in 468 different simulation scenarios. For all scenarios, the significance level of the one-sided hypothesis test is set to $\alpha =5\%$. Accordingly, $\alpha$ is set to $10\%$ for calculating two-sided CIs. In scenarios in which the non-inferiority margin $\delta$ is equal to the true risk difference $\uptheta$, power is equal to the type I error rate. For the VISIT STROKE study, the sample size was planned for $\theta =0$, $\alpha =5\%$, and a probability of 80% for the marginal probabilities ${p}_{1,+}$ and ${p}_{+,1}$, as well as for that of concordant pairs (${p}_{\text{1,1}}+{p}_{\text{0,0}}=0.8$), which corresponds to a within-patient correlation of $\rho =0.37$ (for details see [9]) and resulted in a calculated sample size of 507 for a power of 80% based on the test by Nam. Throughout all simulation scenarios, we also stick to marginal probabilities of ${p}_{1,+}={p}_{+,1}=0.8$.

Correlated binary data are generated using the bindata package [25] in R [26]. Missingness is imposed by generating a random vector of indicators $${\varvec{g}}=({g}_{1}, ..., {g}_{n})$$ as independently and identically distributed random variables following a multinomial distribution and taking values 1, 2, and 3 with probabilities specified above. We set the first ($${y}_{k=1,c}$$) or second observation ($${y}_{k=2,c})$$ to missing if $${g}_{c}$$ equals 1 or 2, respectively, and keep the observed outcome if $${g}_{c}$$ equals 3. Thus, the missing mechanism is either MCAR (for the scenarios with balanced missings between treatment groups) or MAR (for the scenarios with imbalanced missings) and does never depend on the outcome variable (given the treatment group $$k$$). For each simulation scenario, we generate 10′000 random datasets from which we calculate power, interval width, and coverage probability. Power and coverage probability correspond to the proportion of datasets for which the null hypothesis is rejected (power) or the CI includes the true value $$\theta$$ (coverage probability). For the scenarios with $$\delta =0$$, power is expected to be close to the nominal one-sided significance level ($$\alpha =5\%)$$. Irrespective of the simulation scenario, coverage probabilities are always expected to be close to 1 minus the two-sided confidence level of 10%. We calculate the standard error of the random variation due to Monte-Carlo sampling as proposed in [27] for both power and coverage. Interval width is calculated as the upper 90% CI boundary minus the corresponding lower boundary. Other than for power and coverage, there is no nominal or target value for the intervals. If the type I error rate is respected, a lower interval width is preferred over wider ones. For the GEE approach, we used the R package geepack [28]. Note that our simulation setup is similar to the ones used in previous studies [9, 10].

## Results

### Simulation results: non-inferiority margin ${\varvec{\delta}}$ = true risk difference ${\varvec{\theta}}$

#### Power

**Fig. 1:**
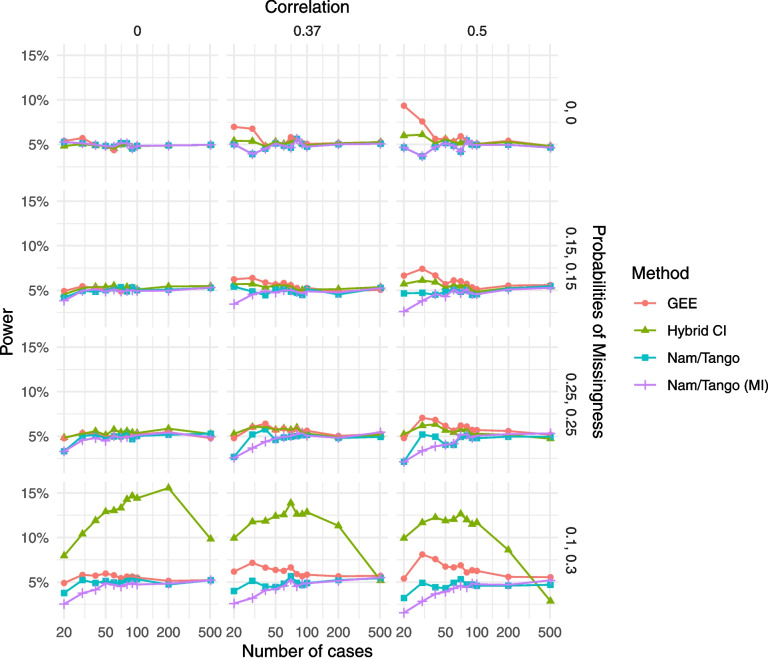
Power for the scenarios with non-inferiority margin $\delta =0.05$ and true risk difference $\theta =0.05$; power equals the type I error rate with nominal level of 5%

In scenarios where the non-inferiority margin is equal to the true underlying risk difference ($\delta =\uptheta$), the power should be close to the nominal one-sided alpha level. For the case of no missingness (first row in Fig. 1), the power of the four methods is similar throughout all scenarios and close to the nominal level of 5%. Note that Nam/Tango and Nam/Tango (MI) do not necessarily yield the same results as the former is based on the score function, while the latter is calculated as a Wald-type CI. Similarly, for all scenarios with balanced missingnes (MCAR), all methods approach the power of 5% with increasing sample size and are below that level for smaller sample sizes. For the scenario with unbalanced missingness (MAR), Nam/Tango and Nam/Tango (MI) again yield similar results close to the expected 5%. The power of the CIs of the GEE method lies slightly above 5% for sample sizes smaller than 100 (up to 9.34% for the small sample case of $n=20$). In the case of many unbalanced missings and a high within-patient correlation, the power of the GEE CIs exceeds 5% even for a sample size of 200 and converges to the nominal level only for larger sample sizes. For the hybrid CI, we observe a power (i.e., a false-positive rate) much above the nominal level, almost 20% in some scenarios, and it falls below 5% for high within-patient correlations and large sample sizes. The results presented in Fig. 1 and those from all other simulation scenarios are provided in tabular form in the supplementary material. The supplementary tables also display the standard error of the random variation due to Monte-Carlo sampling. Across all scenarios, the standard error is sufficiently small to confirm that sampling error does not influence the main findings and results (Supplementary Tables 1-3).

#### Coverage probabilities

**Fig. 2:**
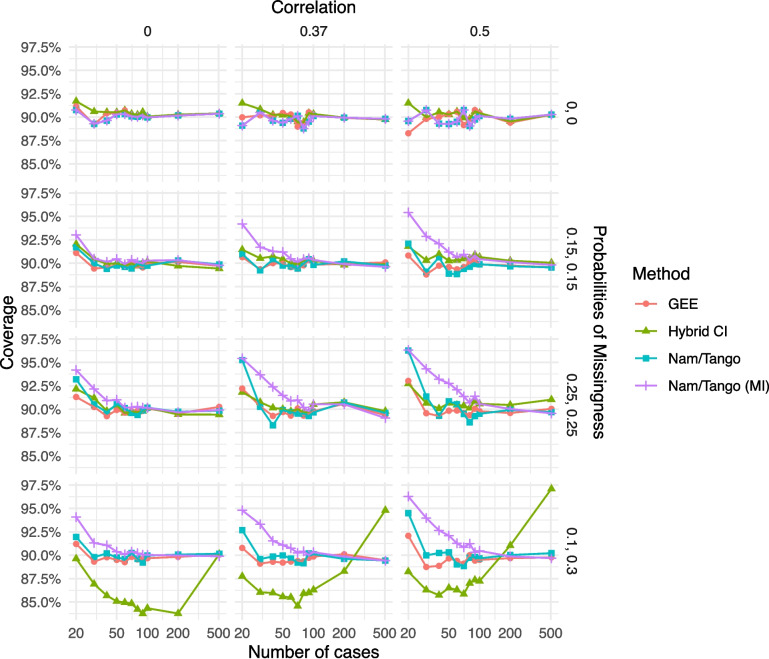
Coverage probabilities for the scenarios with non-inferiority margin $\delta =0.05$ and true risk difference $\theta =0.05$; all but one method have coverage levels of at least 90%

Assessing the coverage probabilities of the intervals for the same scenarios with $\delta =\uptheta =0.05$, the results are similar to the ones observed for power: for the case of MCAR, all methods yield coverage probabilities close to (or above) the nominal level of 90%. For the case of MAR, coverage probabilities of the GEE sometimes lie slightly below the 90% level for small sample sizes up to 100. In comparison, the hybrid CI again shows probabilities much below 90%, contrary to all other methods (Fig. 2).

### Simulation results: non-inferiority margin δ > true risk difference θ 

#### Power

Next, we consider results for scenarios with $\delta =0.05$, where the true risk difference $(\theta =0)$ is smaller than the non-inferiority margin.

**Fig. 3:**
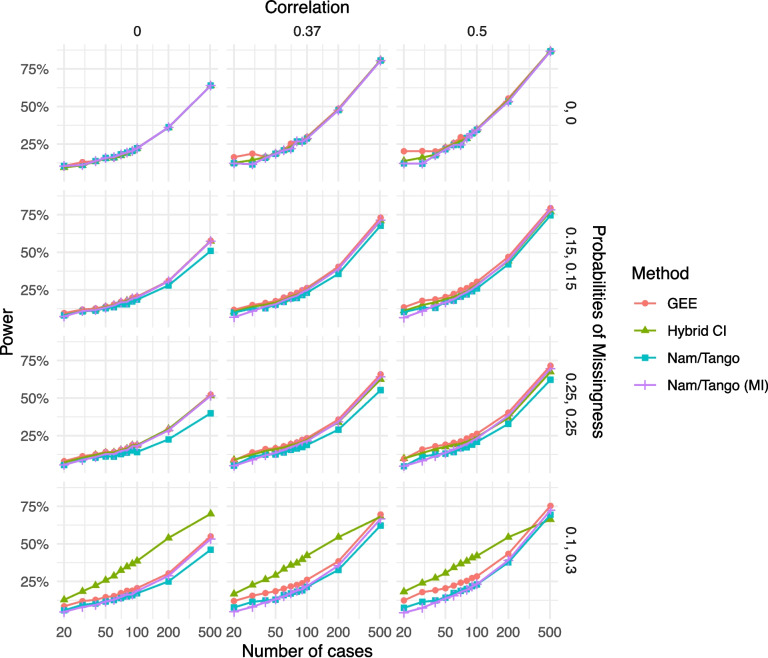
Power for the scenarios with non-inferiority margin $\delta =0.05$ and true risk difference $\theta =0$; note that the hybrid CI has an inflated type I error rate

Assessing the power in the case of no missingness (first row in Fig. 3), all methods yield similar results and show the same expected pattern of increasing power with increasing sample size. Note that for the scenario with $\rho =0.37$, the power of all methods is exactly 80% for $n=507$, which is the result of the initial sample size calculation for the VISIT STROKE study. Furthermore, the power of all methods increases with increasing within-patient correlation $\rho$. For example, for $n=507$, the power of all methods is close to 63% for a within-patient correlation of $\rho =0$ compared to 80% power for the case of $\rho =0.37$.

For the case of MCAR (rows 2 and 3 in Fig. 3), the power of all methods decreases with increasing missing probabilities, but differently between the methods. The decrease in power is most pronounced for the method based on completely observed pairs only (Nam/Tango). For all other methods, the power is similar for the scenarios with $\rho =0$, but slight differences are visible for the scenarios with a positive correlation coefficient. Concretely, the power of the GEE is slightly but consistently higher than those of Nam/Tango (MI) and the hybrid CI, especially in scenarios with a high positive within-patient correlation. Yet, the hybrid CI and Nam/Tango (MI) are improvements over the original Nam/Tango intervals.

For scenarios with MAR, the hybrid CI again shows the highest power – but recall that the type I error was inflated under the null hypothesis. Again, the GEE has the highest power of all methods, even for larger sample sizes. MI slightly increases the power compared to the original Nam/Tango intervals based only on completely observed patients. As shown by the standard error of the random variation due to Monte-Carlo sampling in the supplementary material, we can be confident that the observed differences are not merely due to random sampling error (Supplementary Tables 10-12).

#### Coverage probabilities

**Fig. 4:**
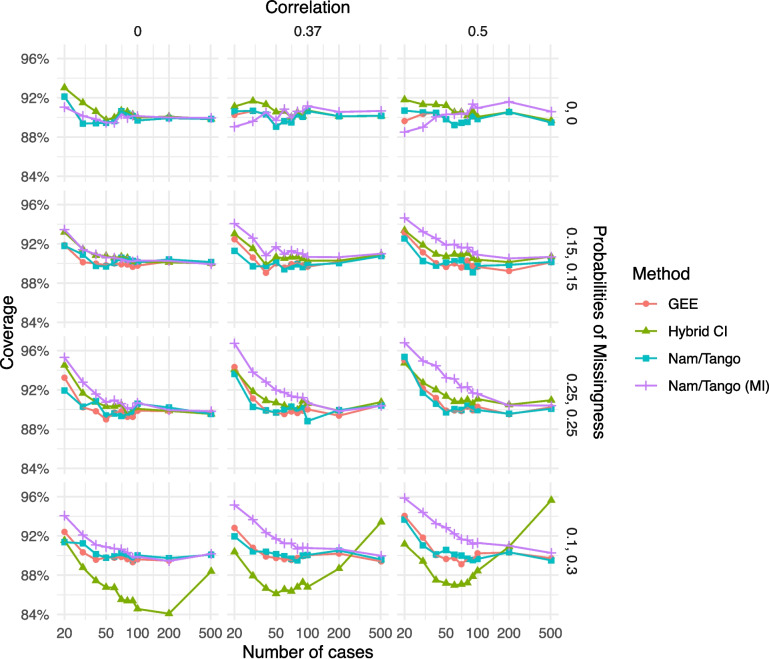
Coverage probabilities for the scenarios with non-inferiority margin $\delta =0.05$ and true risk difference $\theta =0$; all but one method have coverage levels of at least 90%

For the coverage probabilities, we again observe coverage probabilities clearly below 90% for the hybrid CI for the scenarios with the missing mechanism being MAR (Fig. 4). The coverage probabilities of all other methods are above the expected 90% and approach the nominal confidence level with increasing sample size.

#### Interval width

**Fig. 5:**
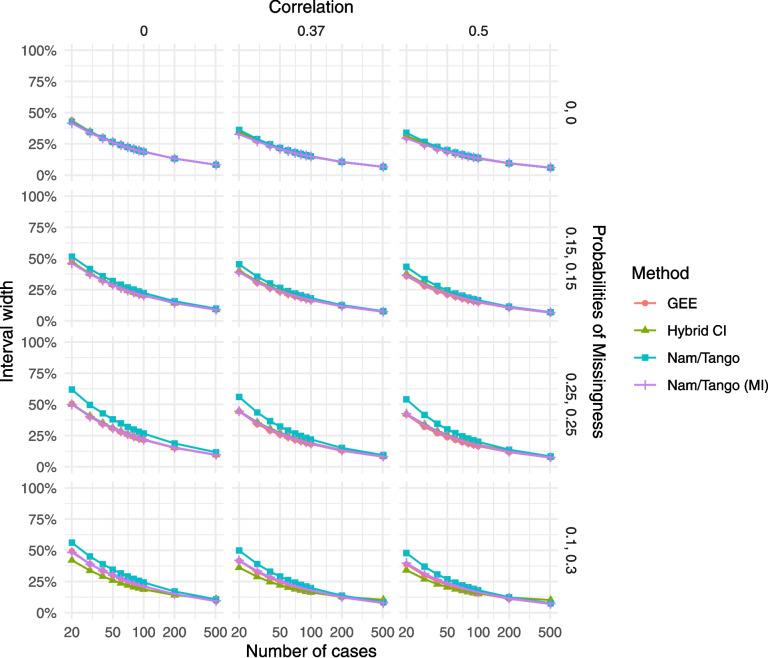
Interval width for the scenarios with non-inferiority margin $\delta =0.05$ and true risk difference $\theta =0$; note that the hybrid CI has an inflated type I error rate

In addition to power and sample size, we can also assess the interval width, again for the scenarios with $\delta =0.05$ (Fig. 5).

For all scenarios with MCAR, the CIs of all methods are either almost identical (in the case of no missingness) or show slight differences, favoring the GEE as the method with the narrowest CIs. Again, in the MAR scenario, the hybrid CI has the narrowest width, bearing in mind the inflated type I error rate.

The simulation scenarios with $\delta =0.1$ show identical patterns, so we do not display them here. The supplemental material contains results for these scenarios and those concerning interval width in the scenarios above.

### An illustrative example

As an illustrative example, we reconsider the sample size calculation for the VISIT STROKE study. The primary hypothesis of this study is the non-inferiority of a telemedicine-based ward-round in acute stroke patients by a remote neurologist compared to a conventional ward-round by an on-site neurologist. The binary endpoint based on quality indicators was planned to be analyzed via Nam [6] test with CIs for the risk difference calculated according to Tango [7]. Based on the assumed parameters $\theta =0$, $\alpha =5\%$, a probability of 80% for the marginal probabilities ${p}_{1,+}$ and ${p}_{+,1}$, as well as for that of concordant pairs (${p}_{\text{1,1}}+{p}_{\text{0,0}}=0.8$), the original sample size calculation resulted in 507 patients needed to achieve a power of 80% for a non-inferiority margin of $\delta =0.05.$ For illustrative purposes, we assume now that at the time of study planning, a missing probability of 20% for the assessment of either the on-site or the telemedicine-based ward-round had been expected. As the Nam/Tango CIs disregard all information from pairs with incompletely observed pairs, the method would have required a sample size of 634 patients in this case. As a comparison, we calculated the required sample size for the alternative methods Nam/Tango (MI) and the proposed GEE approach, both based on a simulation approach with again 10′000 random samples from the data. Since the type I error of the hybrid interval is likely inflated in the case of MAR, we do not consider it here. R code for the exemplary sample size calculation can be found in the supplemental material.

For both the Nam/Tango (MI) and the GEE approach, the simulation-based approach leads to small random variation that causes the power not to be strictly increasing with sample size. We chose a conservative approach and, for each method, reported the minimum sample size for which the power is above 80% and consistently so for all larger sample sizes. This way, improving the Nam/Tango interval by multiple imputation decreases the required sample size to 594, an improvement of about 6.3% compared to the Nam/Tango intervals without imputation. The proposed GEE approach is slightly more efficient and leads to a required sample size of 590 patients, corresponding to a reduction in sample size of 7.0% compared to the intervals by Nam/Tango.

## Discussion

For the analysis of binary matched-pairs data, many analyses still rely on the methods developed by Nam [6] and Tango [7]. These methods have the advantage of closed-form sample size formulas which are implemented in standard software. However, these methods are based solely on completely observed pairs. We reviewed several approaches for improving upon these methods in the case of missing data: multiple imputation, hybrid CIs, and a GEE approach, which all additionally make use of information from incomplete pairs. For the case of no missingness, our simulations show that these methods are non-inferior to the test by Nam (and the interval by Tango) in terms of type I error rate, power, and coverage probability. For the case of MCAR, all proposed methods showed consistent improvement in power and interval width compared to the method based on completely observed pairs only. As expected, the size of the improvement increased with increasing missing probabilities. From all the methods compared, the GEE approach had slightly more power and narrower CIs than the hybrid CIs and the MI approach. For scenarios with unbalanced missings (MAR), the GEE approach shows a slightly inflated type I error rate, but it approaches the nominal level with sample sizes greater than 100. In MAR scenarios with a high within-patient correlation, power even exceeds the nominal level up to a sample size of 200. Under the alternative, it again performed better than the intervals by Tango (with and without MI) in terms of power and interval width. The hybrid CI showed an even higher power, shorter interval width, but also an inflated type I error of more than 10%. Thus, this method cannot be recommended in settings with unbalanced missing data (MAR). In the original paper by Tang et al. [10], the authors considered one scenario with MAR. Still, they presented their simulation results in combination with a majority of balanced designs, which could explain why the authors concluded that these intervals also work well in the MAR setting.

These results suggest that the GEE is as good as the original interval by Tango in terms of power and interval width for scenarios without missing data. As long as the missing mechanism is MCAR, the GEE consistently improves over the Tango interval (with and without MI). For scenarios with MAR, GEE shows a slightly inflated type I error rate for smaller sample sizes, in our settings up to a sample size of 100 or – in cases with unbalanced missings and a high within-patient correlation – even up to a sample size of 200. Thus, for settings with MAR, small sample sizes, and high missing probabilities, alternative methods should be considered, as the approximate CIs of the GEE may not guarantee a nominal type I error rate. In the simulation scenarios, we did not investigate a setting where missings occur not at random (MNAR). In this case, all methods are expected to lead to biased results, which can only be addressed by collecting more data on the causes of missingness or by making additional but non-testable assumptions about the missing data mechanism [16]. Furthermore, we only explicitly dealt with missing observations for patients with at least one observed outcome, excluding those with both outcomes missing. This exclusion implicitly assumes that the studied effect does not differ between patients with no observations and those with at least one. While this assumption cannot be empirically tested, differences between these groups could be examined using observed baseline covariates, which could serve as a first plausibility check of this assumption. Additionally, based on additional covariates, MI could be used to impute missing values for patients with both outcomes missing when applying the GEE approach. However, excluding these patients from the analysis is the only practical strategy in the simple scenario investigated here, without additional covariates.

In scenarios with larger sample sizes (or only a few missings), the GEE approach is at least as good as and, in some scenarios, even superior to all other methods considered. In principle, the GEE approach also allows for the consideration of different levels of clustering, such as matched pairs that are additionally clustered within different study centers. The illustrative example showed that even with only moderate probabilities of missingness, both MI and the GEE approach could substantially improve the required sample size compared to the approach by Nam and Tango.

An additional advantage of the GEE approach over the other methods is its ability to incorporate additional covariates. This allows straightforward adjustment for potential confounders and testing for treatment interaction effects by including them in the model. For example, in the VISIT STROKE study, the sequence of telemedicine-based and on-site ward rounds might influence outcomes. The GEE approach enables this sequence to be included as a covariate, allowing its effect to be controlled. Furthermore, even if covariates do not act as confounders or interact with the treatment effect, including those strongly correlated with the outcome can reduce the standard error of the estimated treatment effect and, thus, increase statistical power (see e.g., the recommendation for the inclusion of relevant patient-level covariates in the ICH E9 guideline [29]).

One limitation of the GEE approach is the absence of a closed-form sample size formula. While it does exist in other settings [30, 31], the logistic regression does not provide a direct estimate of the risk difference and its variance in our setting. However, simulation-based sample size planning could be performed if a sample size calculation based on the GEE approach is needed. If the initial sample size was planned based on the Nam test, our results suggest that instead of relying on the Nam method for the final analysis, it can also be carried out with the GEE approach. This is even more true if unexpected missing data occur or if the analysis has to account for additional variables, in which case the GEE approach should be superior to the methods by Nam [6] and Tango [7].

## Conclusions

Our simulation results suggest that CIs based on the GEE approach achieve levels of power and interval width comparable to those of all other methods, including the interval by Tango [7], when no missing data is present. In MCAR settings, all methods considered are an improvement over Tango’s intervals, with the GEE intervals being slightly superior in terms of power and interval width. In settings with unbalanced missing data, we observe inflated type I error rates for the hybrid method, while the GEE still demonstrates desirable behavior under the null hypothesis for moderate to large sample sizes and has the highest power under alternative settings. Overall, our findings indicate that the GEE approach is suitable for estimating CIs even when the initial sample size calculation was based on methods by Nam [6] or Tango [7]. It is non-inferior in settings with completely observed pairs and superior in settings where only one observation is available for some pairs. Additionally, and uniquely among all methods considered, the GEE approach allows to control for the effects of additional covariates.

## Supplementary Information

Supplementary Material 1.

Supplementary Material 2.

## Data Availability

Methods and the reconsideration of the sample size calculation are available as R scripts as supplemental material.

## Abbreviations

CI: Confidence interval
GEE: Generalized estimating equation
MAR: Missing at random
MCAR: Missing completely at random
MI: Multiple imputation
