# Supplementary material for: Assessing non-inferiority for binary matched-pairs data with missing values: a powerful and flexible GEE approach based on the risk difference
Source: BMC Med Res Methodol. 2025 Feb 27;25:53. doi: 10.1186/s12874-025-02497-2 (PMC11866877; doi:10.1186/s12874-025-02497-2)
Supplement: Supplementary file 1 — Supplementary Material 1. [file 12874_2025_2497_MOESM1_ESM.docx]

**Supplementary information for *Assessing non-inferiority for binary matched-pairs data with missing values: a powerful and flexible GEE approach based on the risk difference*: Power, coverage probabilities and interval widths for the simulation scenarios**

*Figure 1: Interval width for the scenarios with* *non-inferiority margin* $\delta=0.05$ *and true risk difference* $\theta=0.05$*; note that the hybrid confidence interval (CI) has an inflated type-I-error-rate*
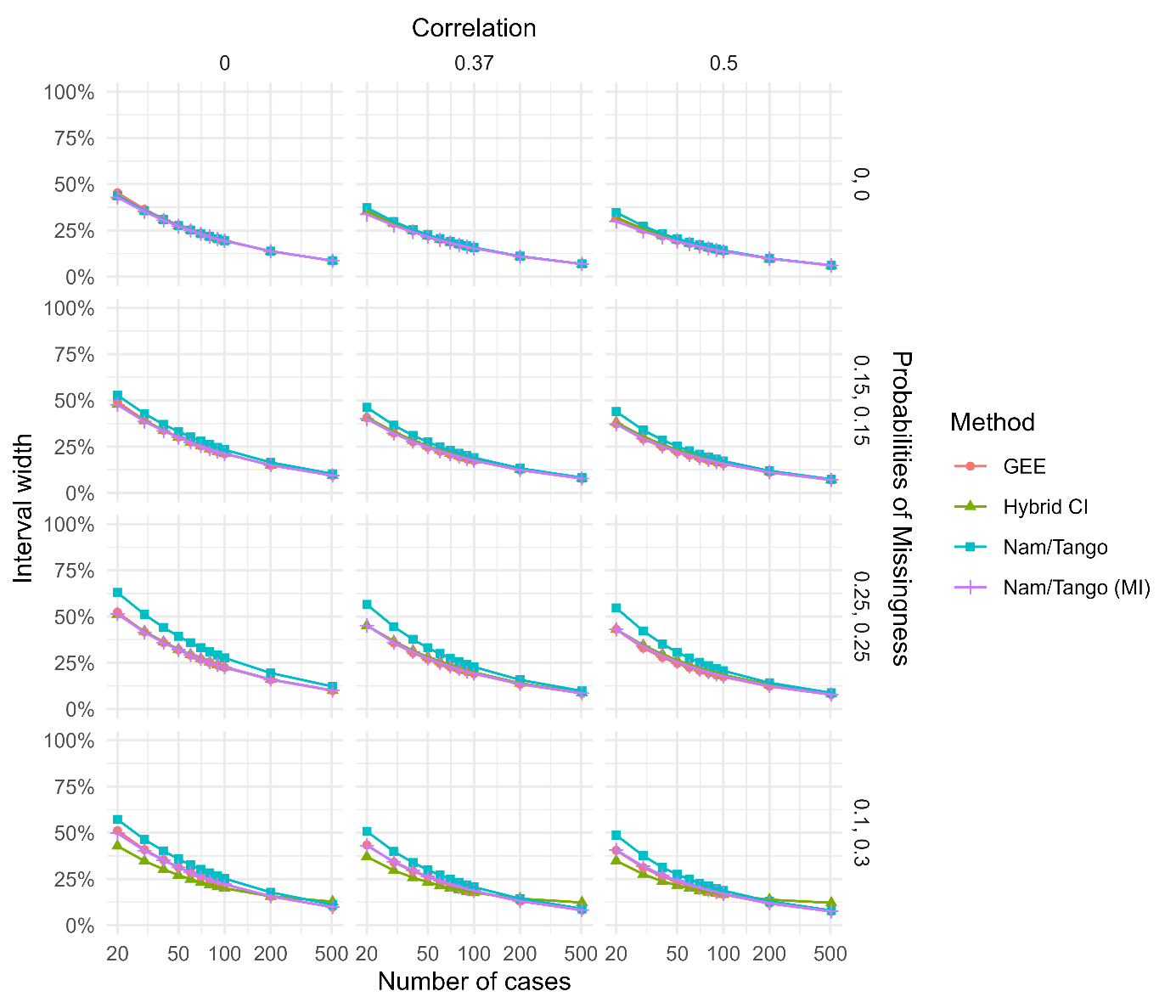


Figure 2: Power for the scenarios with non-inferiority margin $\delta=0.1$ and true risk difference $\theta=0$; note that the hybrid confidence interval (CI) has an inflated type-I-error-rate


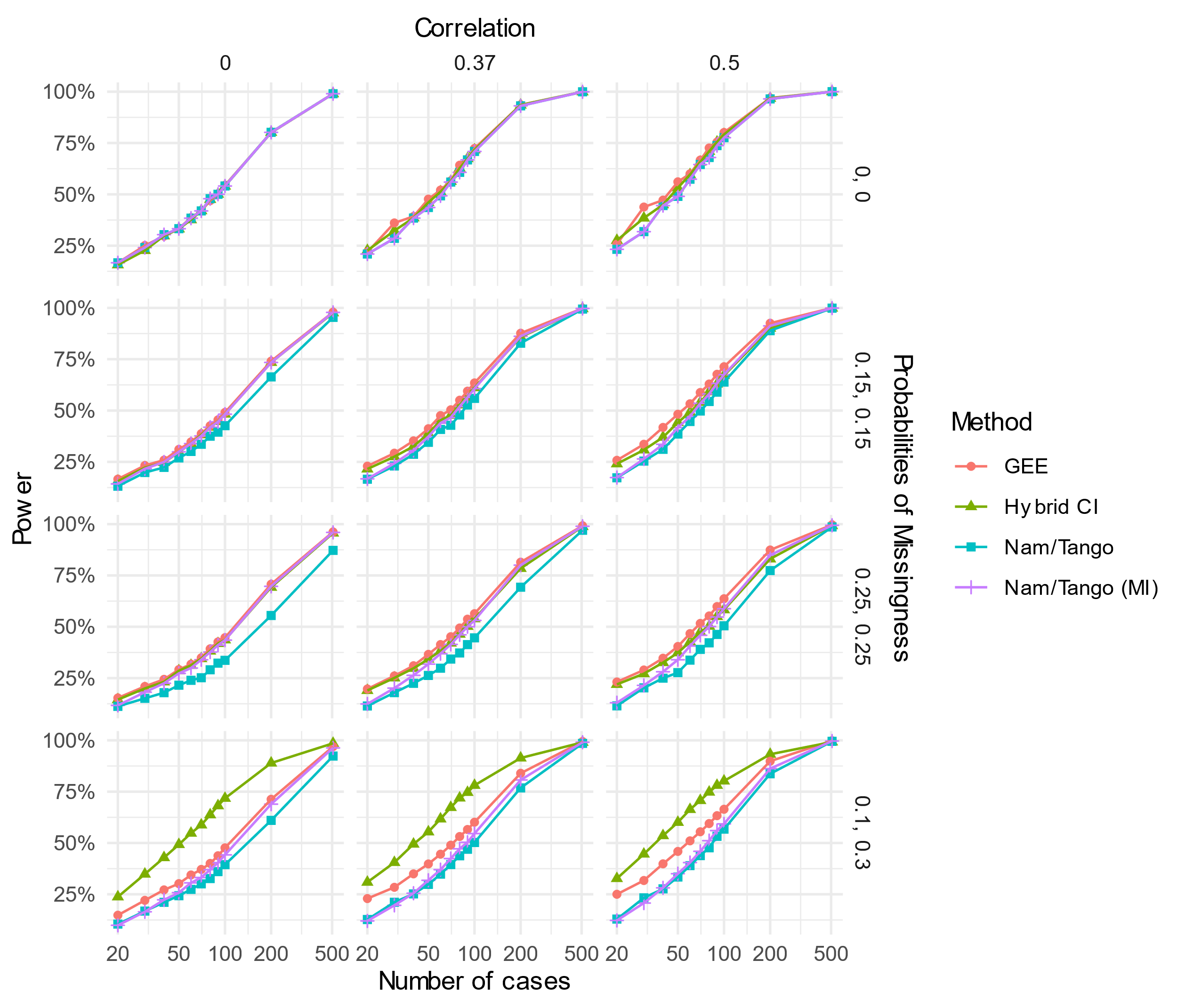


Figure 3: Coverage for the scenarios with non-inferiority margin $\delta=0.1$ and true risk difference $\theta=0$; note that the hybrid confidence interval (CI) has an inflated type-I-error-rate


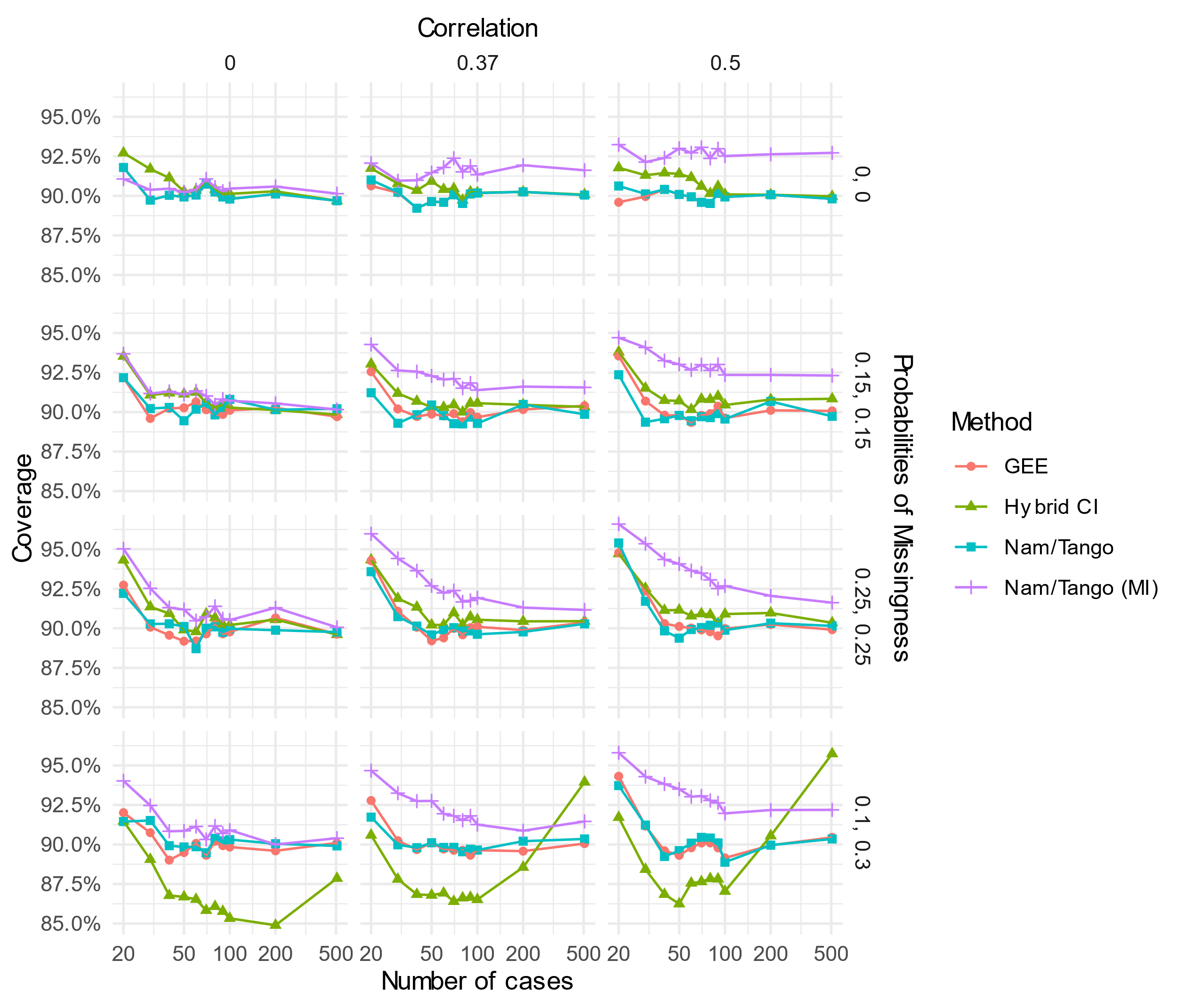


Figure 4: Interval width for the scenarios with non-inferiority margin $\delta=0.1$ and true risk difference $\theta=0$; note that the hybrid confidence interval (CI) has an inflated type-I-error-rate


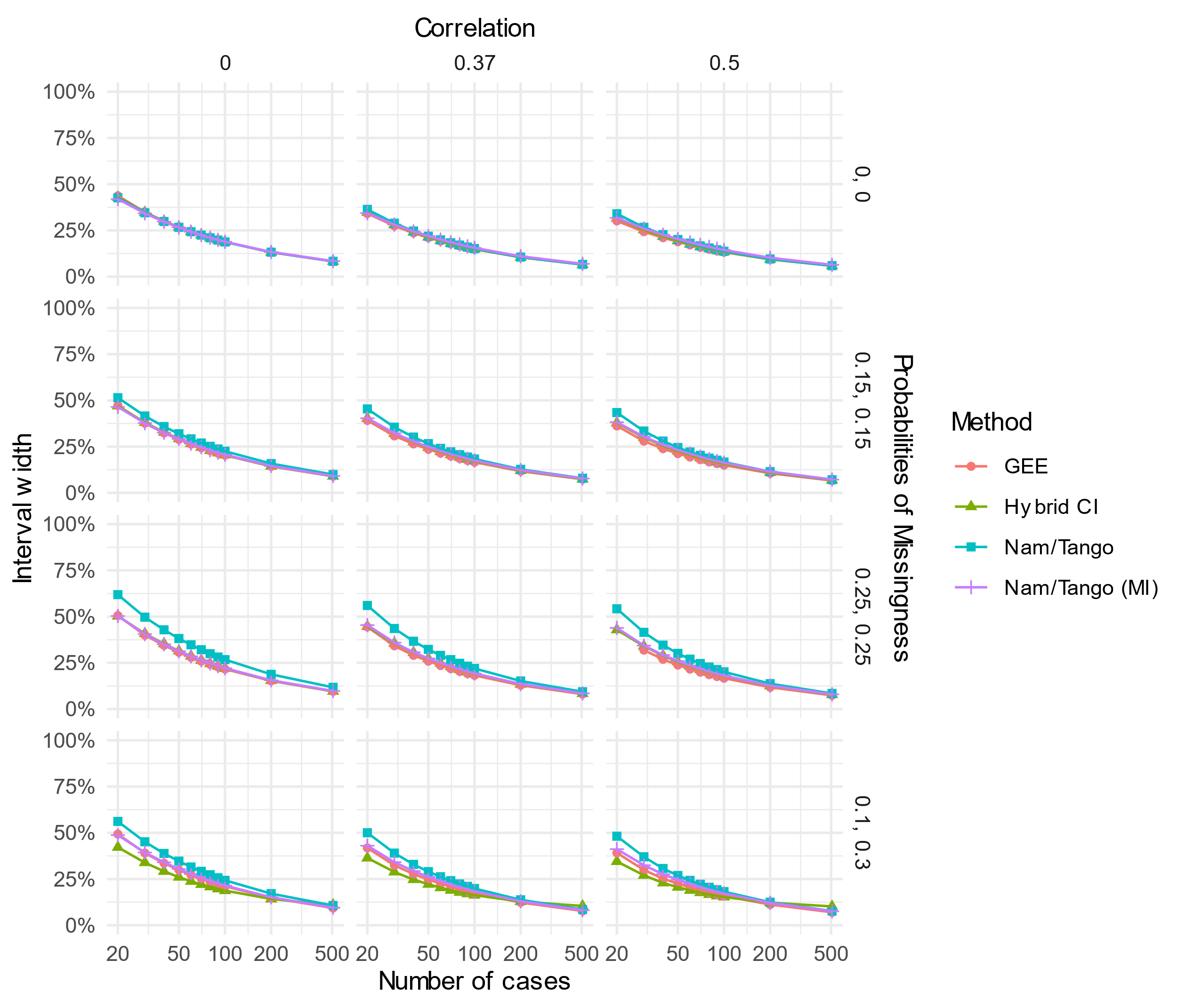


Table 1: Power for the scenarios with non-inferiority margin $\delta=0.05$, true risk difference $\theta=0.05$ and within-patient correlation $\rho=0$; Monte-Carlo standard error in parenthesis

| Probability of missingness per group | Cases | GEE | Hybrid CI | Nam/Tango | Nam/Tango with multiple imputation |
| --- | --- | --- | --- | --- | --- |
| 0, 0 | 20 | 0.0539 (0.002) | 0.0483 (0.002) | 0.0526 (0.002) | 0.0526 (0.002) |
| 0, 0 | 30 | 0.0571 (0.002) | 0.0503 (0.002) | 0.0513 (0.002) | 0.0513 (0.002) |
| 0, 0 | 40 | 0.0494 (0.002) | 0.0487 (0.002) | 0.0494 (0.002) | 0.0494 (0.002) |
| 0, 0 | 50 | 0.0480 (0.002) | 0.0481 (0.002) | 0.0482 (0.002) | 0.0482 (0.002) |
| 0, 0 | 60 | 0.0436 (0.002) | 0.0453 (0.002) | 0.0475 (0.002) | 0.0475 (0.002) |
| 0, 0 | 70 | 0.0516 (0.002) | 0.0496 (0.002) | 0.0514 (0.002) | 0.0514 (0.002) |
| 0, 0 | 80 | 0.0512 (0.002) | 0.0502 (0.002) | 0.0512 (0.002) | 0.0512 (0.002) |
| 0, 0 | 90 | 0.0454 (0.002) | 0.0464 (0.002) | 0.0455 (0.002) | 0.0455 (0.002) |
| 0, 0 | 100 | 0.0483 (0.002) | 0.0481 (0.002) | 0.0483 (0.002) | 0.0483 (0.002) |
| 0, 0 | 200 | 0.0488 (0.002) | 0.0488 (0.002) | 0.0488 (0.002) | 0.0488 (0.002) |
| 0, 0 | 507 | 0.0495 (0.002) | 0.0495 (0.002) | 0.0495 (0.002) | 0.0495 (0.002) |
| 0.15, 0.15 | 20 | 0.0492 (0.002) | 0.0456 (0.002) | 0.0418 (0.002) | 0.0383 (0.002) |
| 0.15, 0.15 | 30 | 0.0545 (0.002) | 0.0524 (0.002) | 0.0495 (0.002) | 0.0494 (0.002) |
| 0.15, 0.15 | 40 | 0.0524 (0.002) | 0.0539 (0.002) | 0.0482 (0.002) | 0.0511 (0.002) |
| 0.15, 0.15 | 50 | 0.0506 (0.002) | 0.0537 (0.002) | 0.0501 (0.002) | 0.0487 (0.002) |
| 0.15, 0.15 | 60 | 0.0526 (0.002) | 0.0553 (0.002) | 0.0523 (0.002) | 0.0511 (0.002) |
| 0.15, 0.15 | 70 | 0.0487 (0.002) | 0.0515 (0.002) | 0.0535 (0.002) | 0.0486 (0.002) |
| 0.15, 0.15 | 80 | 0.0498 (0.002) | 0.0537 (0.002) | 0.0487 (0.002) | 0.0495 (0.002) |
| 0.15, 0.15 | 90 | 0.0524 (0.002) | 0.0524 (0.002) | 0.0532 (0.002) | 0.0501 (0.002) |
| 0.15, 0.15 | 100 | 0.0498 (0.002) | 0.0509 (0.002) | 0.0500 (0.002) | 0.0493 (0.002) |
| 0.15, 0.15 | 200 | 0.0503 (0.002) | 0.0544 (0.002) | 0.0508 (0.002) | 0.0496 (0.002) |
| 0.15, 0.15 | 507 | 0.0527 (0.002) | 0.0547 (0.002) | 0.0527 (0.002) | 0.0532 (0.002) |
| 0.25, 0.25 | 20 | 0.0477 (0.002) | 0.0484 (0.002) | 0.0332 (0.002) | 0.0331 (0.002) |
| 0.25, 0.25 | 30 | 0.0536 (0.002) | 0.0528 (0.002) | 0.0503 (0.002) | 0.0456 (0.002) |
| 0.25, 0.25 | 40 | 0.0523 (0.002) | 0.0556 (0.002) | 0.0515 (0.002) | 0.0479 (0.002) |
| 0.25, 0.25 | 50 | 0.0499 (0.002) | 0.0513 (0.002) | 0.0480 (0.002) | 0.0448 (0.002) |
| 0.25, 0.25 | 60 | 0.0506 (0.002) | 0.0574 (0.002) | 0.0497 (0.002) | 0.0503 (0.002) |
| 0.25, 0.25 | 70 | 0.0488 (0.002) | 0.0544 (0.002) | 0.0501 (0.002) | 0.0486 (0.002) |
| 0.25, 0.25 | 80 | 0.0503 (0.002) | 0.0557 (0.002) | 0.0534 (0.002) | 0.0493 (0.002) |
| 0.25, 0.25 | 90 | 0.0502 (0.002) | 0.0541 (0.002) | 0.0468 (0.002) | 0.0497 (0.002) |
| 0.25, 0.25 | 100 | 0.0519 (0.002) | 0.0536 (0.002) | 0.0503 (0.002) | 0.0512 (0.002) |
| 0.25, 0.25 | 200 | 0.0546 (0.002) | 0.0585 (0.002) | 0.0516 (0.002) | 0.0539 (0.002) |
| 0.25, 0.25 | 507 | 0.0478 (0.002) | 0.0525 (0.002) | 0.0529 (0.002) | 0.0490 (0.002) |
| 0.1, 0.3 | 20 | 0.0490 (0.002) | 0.0796 (0.003) | 0.0378 (0.002) | 0.0252 (0.002) |
| 0.1, 0.3 | 30 | 0.0581 (0.002) | 0.1040 (0.003) | 0.0522 (0.002) | 0.0376 (0.002) |
| 0.1, 0.3 | 40 | 0.0573 (0.002) | 0.1191 (0.003) | 0.0490 (0.002) | 0.0414 (0.002) |
| 0.1, 0.3 | 50 | 0.0596 (0.002) | 0.1290 (0.003) | 0.0512 (0.002) | 0.0485 (0.002) |
| 0.1, 0.3 | 60 | 0.0577 (0.002) | 0.1303 (0.003) | 0.0496 (0.002) | 0.0469 (0.002) |
| 0.1, 0.3 | 70 | 0.0544 (0.002) | 0.1331 (0.003) | 0.0489 (0.002) | 0.0451 (0.002) |
| 0.1, 0.3 | 80 | 0.0562 (0.002) | 0.1429 (0.003) | 0.0523 (0.002) | 0.0476 (0.002) |
| 0.1, 0.3 | 90 | 0.0562 (0.002) | 0.1467 (0.004) | 0.0540 (0.002) | 0.0489 (0.002) |
| 0.1, 0.3 | 100 | 0.0551 (0.002) | 0.1441 (0.004) | 0.0532 (0.002) | 0.0473 (0.002) |
| 0.1, 0.3 | 200 | 0.0514 (0.002) | 0.1556 (0.004) | 0.0473 (0.002) | 0.0483 (0.002) |
| 0.1, 0.3 | 507 | 0.0522 (0.002) | 0.0984 (0.003) | 0.0521 (0.002) | 0.0521 (0.002) |

Table 2: Power for the scenarios with non-inferiority margin $\delta=0.05$, true risk difference $\theta=0.05$ and within-patient correlation $\rho=0.37$; Monte-Carlo standard error in parenthesis

| Probability of missingness per group | | Cases | | GEE | Hybrid CI | Nam/Tango | Nam/Tango with multiple imputation |
| --- | --- | --- | --- | --- | --- | --- | --- |
| 0, 0 | 20 | | 0.0697 (0.003) | | 0.0541 (0.002) | 0.0502 (0.002) | 0.0502 (0.002) |
| 0, 0 | 30 | | 0.0676 (0.003) | | 0.0537 (0.002) | 0.0391 (0.002) | 0.0391 (0.002) |
| 0, 0 | 40 | | 0.0484 (0.002) | | 0.0479 (0.002) | 0.0452 (0.002) | 0.0452 (0.002) |
| 0, 0 | 50 | | 0.0518 (0.002) | | 0.0532 (0.002) | 0.0511 (0.002) | 0.0511 (0.002) |
| 0, 0 | 60 | | 0.0490 (0.002) | | 0.0505 (0.002) | 0.0485 (0.002) | 0.0485 (0.002) |
| 0, 0 | 70 | | 0.0581 (0.002) | | 0.0532 (0.002) | 0.0464 (0.002) | 0.0464 (0.002) |
| 0, 0 | 80 | | 0.0562 (0.002) | | 0.0559 (0.002) | 0.0562 (0.002) | 0.0562 (0.002) |
| 0, 0 | 90 | | 0.0506 (0.002) | | 0.0511 (0.002) | 0.0506 (0.002) | 0.0506 (0.002) |
| 0, 0 | 100 | | 0.0505 (0.002) | | 0.0493 (0.002) | 0.0475 (0.002) | 0.0475 (0.002) |
| 0, 0 | 200 | | 0.0515 (0.002) | | 0.0511 (0.002) | 0.0501 (0.002) | 0.0501 (0.002) |
| 0, 0 | 507 | | 0.0526 (0.002) | | 0.0526 (0.002) | 0.0508 (0.002) | 0.0508 (0.002) |
| 0.15, 0.15 | 20 | | 0.0624 (0.002) | | 0.0568 (0.002) | 0.0541 (0.002) | 0.0345 (0.002) |
| 0.15, 0.15 | 30 | | 0.0639 (0.002) | | 0.0572 (0.002) | 0.0487 (0.002) | 0.0453 (0.002) |
| 0.15, 0.15 | 40 | | 0.0587 (0.002) | | 0.0533 (0.002) | 0.0445 (0.002) | 0.0491 (0.002) |
| 0.15, 0.15 | 50 | | 0.0566 (0.002) | | 0.0556 (0.002) | 0.0510 (0.002) | 0.0474 (0.002) |
| 0.15, 0.15 | 60 | | 0.0583 (0.002) | | 0.0556 (0.002) | 0.0517 (0.002) | 0.0502 (0.002) |
| 0.15, 0.15 | 70 | | 0.0558 (0.002) | | 0.0548 (0.002) | 0.0485 (0.002) | 0.0499 (0.002) |
| 0.15, 0.15 | 80 | | 0.0526 (0.002) | | 0.0505 (0.002) | 0.0471 (0.002) | 0.0482 (0.002) |
| 0.15, 0.15 | 90 | | 0.0485 (0.002) | | 0.0503 (0.002) | 0.0448 (0.002) | 0.0463 (0.002) |
| 0.15, 0.15 | 100 | | 0.0527 (0.002) | | 0.0507 (0.002) | 0.0515 (0.002) | 0.0489 (0.002) |
| 0.15, 0.15 | 200 | | 0.0487 (0.002) | | 0.0514 (0.002) | 0.0453 (0.002) | 0.0475 (0.002) |
| 0.15, 0.15 | 507 | | 0.0506 (0.002) | | 0.0536 (0.002) | 0.0528 (0.002) | 0.0525 (0.002) |
| 0.25, 0.25 | 20 | | 0.0478 (0.002) | | 0.0526 (0.002) | 0.0267 (0.002) | 0.0257 (0.002) |
| 0.25, 0.25 | 30 | | 0.0603 (0.002) | | 0.0604 (0.002) | 0.0520 (0.002) | 0.0367 (0.002) |
| 0.25, 0.25 | 40 | | 0.0641 (0.002) | | 0.0604 (0.002) | 0.0576 (0.002) | 0.0439 (0.002) |
| 0.25, 0.25 | 50 | | 0.0569 (0.002) | | 0.0572 (0.002) | 0.0459 (0.002) | 0.0474 (0.002) |
| 0.25, 0.25 | 60 | | 0.0587 (0.002) | | 0.0588 (0.002) | 0.0484 (0.002) | 0.0490 (0.002) |
| 0.25, 0.25 | 70 | | 0.0560 (0.002) | | 0.0571 (0.002) | 0.0494 (0.002) | 0.0501 (0.002) |
| 0.25, 0.25 | 80 | | 0.0582 (0.002) | | 0.0597 (0.002) | 0.0501 (0.002) | 0.0518 (0.002) |
| 0.25, 0.25 | 90 | | 0.0545 (0.002) | | 0.0544 (0.002) | 0.0511 (0.002) | 0.0535 (0.002) |
| 0.25, 0.25 | 100 | | 0.0561 (0.002) | | 0.0530 (0.002) | 0.0515 (0.002) | 0.0506 (0.002) |
| 0.25, 0.25 | 200 | | 0.0503 (0.002) | | 0.0488 (0.002) | 0.0480 (0.002) | 0.0482 (0.002) |
| 0.25, 0.25 | 507 | | 0.0527 (0.002) | | 0.0516 (0.002) | 0.0492 (0.002) | 0.0544 (0.002) |
| 0.1, 0.3 | 20 | | 0.0618 (0.002) | | 0.0992 (0.003) | 0.0400 (0.002) | 0.0260 (0.002) |
| 0.1, 0.3 | 30 | | 0.0716 (0.003) | | 0.1177 (0.003) | 0.0514 (0.002) | 0.0320 (0.002) |
| 0.1, 0.3 | 40 | | 0.0663 (0.002) | | 0.1185 (0.003) | 0.0450 (0.002) | 0.0409 (0.002) |
| 0.1, 0.3 | 50 | | 0.0637 (0.002) | | 0.1240 (0.003) | 0.0445 (0.002) | 0.0423 (0.002) |
| 0.1, 0.3 | 60 | | 0.0626 (0.002) | | 0.1255 (0.003) | 0.0482 (0.002) | 0.0459 (0.002) |
| 0.1, 0.3 | 70 | | 0.0665 (0.002) | | 0.1388 (0.003) | 0.0567 (0.002) | 0.0527 (0.002) |
| 0.1, 0.3 | 80 | | 0.0590 (0.002) | | 0.1263 (0.003) | 0.0492 (0.002) | 0.0452 (0.002) |
| 0.1, 0.3 | 90 | | 0.0566 (0.002) | | 0.1262 (0.003) | 0.0467 (0.002) | 0.0479 (0.002) |
| 0.1, 0.3 | 100 | | 0.0583 (0.002) | | 0.1285 (0.003) | 0.0489 (0.002) | 0.0486 (0.002) |
| 0.1, 0.3 | 200 | | 0.0565 (0.002) | | 0.1132 (0.003) | 0.0523 (0.002) | 0.0515 (0.002) |
| 0.1, 0.3 | 507 | | 0.0571 (0.002) | | 0.0518 (0.002) | 0.0543 (0.002) | 0.0552 (0.002) |

Table 3: Power for the scenarios with non-inferiority margin $\delta=0.05$, true risk difference $\theta=0.05$ and within-patient correlation $\rho=0.5$; Monte-Carlo standard error in parenthesis

| Probability of missingness per group | Cases | GEE | Hybrid CI | Nam/Tango | Nam/Tango with multiple imputation |
| --- | --- | --- | --- | --- | --- |
| 0, 0 | 20 | 0.0934 (0.003) | 0.0598 (0.002) | 0.0466 (0.002) | 0.0466 (0.002) |
| 0, 0 | 30 | 0.0758 (0.003) | 0.0610 (0.002) | 0.0368 (0.002) | 0.0368 (0.002) |
| 0, 0 | 40 | 0.0562 (0.002) | 0.0512 (0.002) | 0.0471 (0.002) | 0.0471 (0.002) |
| 0, 0 | 50 | 0.0560 (0.002) | 0.0559 (0.002) | 0.0514 (0.002) | 0.0514 (0.002) |
| 0, 0 | 60 | 0.0533 (0.002) | 0.0525 (0.002) | 0.0486 (0.002) | 0.0486 (0.002) |
| 0, 0 | 70 | 0.0592 (0.002) | 0.0516 (0.002) | 0.0419 (0.002) | 0.0419 (0.002) |
| 0, 0 | 80 | 0.0546 (0.002) | 0.0542 (0.002) | 0.0545 (0.002) | 0.0545 (0.002) |
| 0, 0 | 90 | 0.0502 (0.002) | 0.0501 (0.002) | 0.0498 (0.002) | 0.0498 (0.002) |
| 0, 0 | 100 | 0.0506 (0.002) | 0.0503 (0.002) | 0.0494 (0.002) | 0.0494 (0.002) |
| 0, 0 | 200 | 0.0540 (0.002) | 0.0527 (0.002) | 0.0496 (0.002) | 0.0496 (0.002) |
| 0, 0 | 507 | 0.0481 (0.002) | 0.0481 (0.002) | 0.0465 (0.002) | 0.0465 (0.002) |
| 0.15, 0.15 | 20 | 0.0665 (0.002) | 0.0571 (0.002) | 0.0466 (0.002) | 0.0262 (0.002) |
| 0.15, 0.15 | 30 | 0.0741 (0.003) | 0.0611 (0.002) | 0.0471 (0.002) | 0.0381 (0.002) |
| 0.15, 0.15 | 40 | 0.0667 (0.002) | 0.0591 (0.002) | 0.0450 (0.002) | 0.0457 (0.002) |
| 0.15, 0.15 | 50 | 0.0570 (0.002) | 0.0533 (0.002) | 0.0483 (0.002) | 0.0430 (0.002) |
| 0.15, 0.15 | 60 | 0.0611 (0.002) | 0.0556 (0.002) | 0.0512 (0.002) | 0.0509 (0.002) |
| 0.15, 0.15 | 70 | 0.0602 (0.002) | 0.0550 (0.002) | 0.0483 (0.002) | 0.0469 (0.002) |
| 0.15, 0.15 | 80 | 0.0573 (0.002) | 0.0559 (0.002) | 0.0505 (0.002) | 0.0509 (0.002) |
| 0.15, 0.15 | 90 | 0.0534 (0.002) | 0.0502 (0.002) | 0.0449 (0.002) | 0.0460 (0.002) |
| 0.15, 0.15 | 100 | 0.0512 (0.002) | 0.0484 (0.002) | 0.0457 (0.002) | 0.0457 (0.002) |
| 0.15, 0.15 | 200 | 0.0553 (0.002) | 0.0526 (0.002) | 0.0521 (0.002) | 0.0509 (0.002) |
| 0.15, 0.15 | 507 | 0.0560 (0.002) | 0.0540 (0.002) | 0.0547 (0.002) | 0.0528 (0.002) |
| 0.25, 0.25 | 20 | 0.0478 (0.002) | 0.0522 (0.002) | 0.0214 (0.001) | 0.0213 (0.001) |
| 0.25, 0.25 | 30 | 0.0706 (0.003) | 0.0619 (0.002) | 0.0520 (0.002) | 0.0337 (0.002) |
| 0.25, 0.25 | 40 | 0.0683 (0.003) | 0.0634 (0.002) | 0.0493 (0.002) | 0.0387 (0.002) |
| 0.25, 0.25 | 50 | 0.0617 (0.002) | 0.0566 (0.002) | 0.0403 (0.002) | 0.0404 (0.002) |
| 0.25, 0.25 | 60 | 0.0563 (0.002) | 0.0543 (0.002) | 0.0403 (0.002) | 0.0418 (0.002) |
| 0.25, 0.25 | 70 | 0.0620 (0.002) | 0.0574 (0.002) | 0.0493 (0.002) | 0.0513 (0.002) |
| 0.25, 0.25 | 80 | 0.0609 (0.002) | 0.0578 (0.002) | 0.0508 (0.002) | 0.0518 (0.002) |
| 0.25, 0.25 | 90 | 0.0571 (0.002) | 0.0520 (0.002) | 0.0480 (0.002) | 0.0481 (0.002) |
| 0.25, 0.25 | 100 | 0.0569 (0.002) | 0.0528 (0.002) | 0.0478 (0.002) | 0.0505 (0.002) |
| 0.25, 0.25 | 200 | 0.0558 (0.002) | 0.0515 (0.002) | 0.0495 (0.002) | 0.0522 (0.002) |
| 0.25, 0.25 | 507 | 0.0517 (0.002) | 0.0472 (0.002) | 0.0495 (0.002) | 0.0532 (0.002) |
| 0.1, 0.3 | 20 | 0.0540 (0.002) | 0.0992 (0.003) | 0.0322 (0.002) | 0.0156 (0.001) |
| 0.1, 0.3 | 30 | 0.0810 (0.003) | 0.1168 (0.003) | 0.0493 (0.002) | 0.0284 (0.002) |
| 0.1, 0.3 | 40 | 0.0758 (0.003) | 0.1225 (0.003) | 0.0443 (0.002) | 0.0368 (0.002) |
| 0.1, 0.3 | 50 | 0.0674 (0.003) | 0.1188 (0.003) | 0.0430 (0.002) | 0.0391 (0.002) |
| 0.1, 0.3 | 60 | 0.0665 (0.002) | 0.1204 (0.003) | 0.0493 (0.002) | 0.0430 (0.002) |
| 0.1, 0.3 | 70 | 0.0688 (0.003) | 0.1265 (0.003) | 0.0533 (0.002) | 0.0460 (0.002) |
| 0.1, 0.3 | 80 | 0.0608 (0.002) | 0.1200 (0.003) | 0.0474 (0.002) | 0.0441 (0.002) |
| 0.1, 0.3 | 90 | 0.0633 (0.002) | 0.1148 (0.003) | 0.0474 (0.002) | 0.0483 (0.002) |
| 0.1, 0.3 | 100 | 0.0626 (0.002) | 0.1168 (0.003) | 0.0459 (0.002) | 0.0475 (0.002) |
| 0.1, 0.3 | 200 | 0.0559 (0.002) | 0.0860 (0.003) | 0.0460 (0.002) | 0.0470 (0.002) |
| 0.1, 0.3 | 507 | 0.0555 (0.002) | 0.0287 (0.002) | 0.0470 (0.002) | 0.0520 (0.002) |

Table 4: Coverage for the scenarios with non-inferiority margin $\delta=0.05$, true risk difference $\theta=0.05$ and within-patient correlation $\rho=0$; Monte-Carlo standard error in parenthesis

| Probability of missingness per group | Cases | GEE | Hybrid CI | Nam/Tango | Nam/Tango with multiple imputation |
| --- | --- | --- | --- | --- | --- |
| 0, 0 | 20 | 0.9115 (0.003) | 0.9170 (0.003) | 0.9076 (0.003) | 0.9076 (0.003) |
| 0, 0 | 30 | 0.8921 (0.003) | 0.9059 (0.003) | 0.8928 (0.003) | 0.8928 (0.003) |
| 0, 0 | 40 | 0.9042 (0.003) | 0.9054 (0.003) | 0.8963 (0.003) | 0.8963 (0.003) |
| 0, 0 | 50 | 0.9046 (0.003) | 0.9052 (0.003) | 0.9029 (0.003) | 0.9029 (0.003) |
| 0, 0 | 60 | 0.9071 (0.003) | 0.9072 (0.003) | 0.9032 (0.003) | 0.9032 (0.003) |
| 0, 0 | 70 | 0.9006 (0.003) | 0.9034 (0.003) | 0.9008 (0.003) | 0.9008 (0.003) |
| 0, 0 | 80 | 0.9000 (0.003) | 0.9025 (0.003) | 0.9003 (0.003) | 0.9003 (0.003) |
| 0, 0 | 90 | 0.9021 (0.003) | 0.9055 (0.003) | 0.9018 (0.003) | 0.9018 (0.003) |
| 0, 0 | 100 | 0.9000 (0.003) | 0.9006 (0.003) | 0.8996 (0.003) | 0.8996 (0.003) |
| 0, 0 | 200 | 0.9016 (0.003) | 0.9027 (0.003) | 0.9017 (0.003) | 0.9017 (0.003) |
| 0, 0 | 507 | 0.9036 (0.003) | 0.9040 (0.003) | 0.9036 (0.003) | 0.9036 (0.003) |
| 0.15, 0.15 | 20 | 0.9109 (0.003) | 0.9204 (0.003) | 0.9170 (0.003) | 0.9302 (0.003) |
| 0.15, 0.15 | 30 | 0.8942 (0.003) | 0.9042 (0.003) | 0.8998 (0.003) | 0.9048 (0.003) |
| 0.15, 0.15 | 40 | 0.8954 (0.003) | 0.8993 (0.003) | 0.8939 (0.003) | 0.9014 (0.003) |
| 0.15, 0.15 | 50 | 0.8980 (0.003) | 0.8996 (0.003) | 0.8975 (0.003) | 0.9044 (0.003) |
| 0.15, 0.15 | 60 | 0.8961 (0.003) | 0.8974 (0.003) | 0.8960 (0.003) | 0.8988 (0.003) |
| 0.15, 0.15 | 70 | 0.9000 (0.003) | 0.8995 (0.003) | 0.8943 (0.003) | 0.9035 (0.003) |
| 0.15, 0.15 | 80 | 0.8978 (0.003) | 0.8970 (0.003) | 0.8993 (0.003) | 0.9008 (0.003) |
| 0.15, 0.15 | 90 | 0.8954 (0.003) | 0.9001 (0.003) | 0.8971 (0.003) | 0.9000 (0.003) |
| 0.15, 0.15 | 100 | 0.8998 (0.003) | 0.9017 (0.003) | 0.8973 (0.003) | 0.9027 (0.003) |
| 0.15, 0.15 | 200 | 0.9013 (0.003) | 0.8970 (0.003) | 0.9028 (0.003) | 0.9032 (0.003) |
| 0.15, 0.15 | 507 | 0.8970 (0.003) | 0.8942 (0.003) | 0.8986 (0.003) | 0.8976 (0.003) |
| 0.25, 0.25 | 20 | 0.9130 (0.003) | 0.9216 (0.003) | 0.9319 (0.003) | 0.9418 (0.002) |
| 0.25, 0.25 | 30 | 0.9023 (0.003) | 0.9119 (0.003) | 0.9052 (0.003) | 0.9216 (0.003) |
| 0.25, 0.25 | 40 | 0.8925 (0.003) | 0.8980 (0.003) | 0.8967 (0.003) | 0.9091 (0.003) |
| 0.25, 0.25 | 50 | 0.8992 (0.003) | 0.9041 (0.003) | 0.9053 (0.003) | 0.9100 (0.003) |
| 0.25, 0.25 | 60 | 0.8963 (0.003) | 0.8958 (0.003) | 0.9011 (0.003) | 0.9016 (0.003) |
| 0.25, 0.25 | 70 | 0.8976 (0.003) | 0.8973 (0.003) | 0.8960 (0.003) | 0.9018 (0.003) |
| 0.25, 0.25 | 80 | 0.8980 (0.003) | 0.8969 (0.003) | 0.8939 (0.003) | 0.9028 (0.003) |
| 0.25, 0.25 | 90 | 0.8981 (0.003) | 0.8977 (0.003) | 0.8988 (0.003) | 0.9016 (0.003) |
| 0.25, 0.25 | 100 | 0.9007 (0.003) | 0.9015 (0.003) | 0.9016 (0.003) | 0.9015 (0.003) |
| 0.25, 0.25 | 200 | 0.8955 (0.003) | 0.8943 (0.003) | 0.8972 (0.003) | 0.8966 (0.003) |
| 0.25, 0.25 | 507 | 0.9024 (0.003) | 0.8942 (0.003) | 0.8984 (0.003) | 0.8991 (0.003) |
| 0.1, 0.3 | 20 | 0.9120 (0.003) | 0.8964 (0.003) | 0.9194 (0.003) | 0.9406 (0.002) |
| 0.1, 0.3 | 30 | 0.8931 (0.003) | 0.8692 (0.003) | 0.8979 (0.003) | 0.9131 (0.003) |
| 0.1, 0.3 | 40 | 0.8978 (0.003) | 0.8568 (0.004) | 0.9019 (0.003) | 0.9106 (0.003) |
| 0.1, 0.3 | 50 | 0.8950 (0.003) | 0.8507 (0.004) | 0.8975 (0.003) | 0.9038 (0.003) |
| 0.1, 0.3 | 60 | 0.8925 (0.003) | 0.8495 (0.004) | 0.8959 (0.003) | 0.9009 (0.003) |
| 0.1, 0.3 | 70 | 0.8982 (0.003) | 0.8483 (0.004) | 0.9024 (0.003) | 0.9037 (0.003) |
| 0.1, 0.3 | 80 | 0.8954 (0.003) | 0.8421 (0.004) | 0.8962 (0.003) | 0.9021 (0.003) |
| 0.1, 0.3 | 90 | 0.8960 (0.003) | 0.8375 (0.004) | 0.8921 (0.003) | 0.9005 (0.003) |
| 0.1, 0.3 | 100 | 0.8967 (0.003) | 0.8433 (0.004) | 0.8996 (0.003) | 0.9003 (0.003) |
| 0.1, 0.3 | 200 | 0.8982 (0.003) | 0.8377 (0.004) | 0.9006 (0.003) | 0.8997 (0.003) |
| 0.1, 0.3 | 507 | 0.9006 (0.003) | 0.9012 (0.003) | 0.9016 (0.003) | 0.8988 (0.003) |

Table 5: Coverage for the scenarios with non-inferiority margin $\delta=0.05$, true risk difference $\theta=0.05$ and within-patient correlation $\rho=0.37$; Monte-Carlo standard error in parenthesis

| Probability of missingness per group | Cases | GEE | Hybrid CI | Nam/Tango | Nam/Tango with multiple imputation |
| --- | --- | --- | --- | --- | --- |
| 0, 0 | 20 | 0.8997 (0.003) | 0.9149 (0.003) | 0.8909 (0.003) | 0.8909 (0.003) |
| 0, 0 | 30 | 0.9019 (0.003) | 0.9084 (0.003) | 0.9048 (0.003) | 0.9048 (0.003) |
| 0, 0 | 40 | 0.9017 (0.003) | 0.9025 (0.003) | 0.8962 (0.003) | 0.8962 (0.003) |
| 0, 0 | 50 | 0.9044 (0.003) | 0.9024 (0.003) | 0.8939 (0.003) | 0.8939 (0.003) |
| 0, 0 | 60 | 0.9028 (0.003) | 0.9011 (0.003) | 0.8981 (0.003) | 0.8981 (0.003) |
| 0, 0 | 70 | 0.8898 (0.003) | 0.8950 (0.003) | 0.9013 (0.003) | 0.9013 (0.003) |
| 0, 0 | 80 | 0.8941 (0.003) | 0.8945 (0.003) | 0.8878 (0.003) | 0.8878 (0.003) |
| 0, 0 | 90 | 0.9050 (0.003) | 0.9043 (0.003) | 0.8952 (0.003) | 0.8952 (0.003) |
| 0, 0 | 100 | 0.9021 (0.003) | 0.9033 (0.003) | 0.9011 (0.003) | 0.9011 (0.003) |
| 0, 0 | 200 | 0.8991 (0.003) | 0.8995 (0.003) | 0.8994 (0.003) | 0.8994 (0.003) |
| 0, 0 | 507 | 0.8974 (0.003) | 0.8974 (0.003) | 0.8981 (0.003) | 0.8981 (0.003) |
| 0.15, 0.15 | 20 | 0.9064 (0.003) | 0.9144 (0.003) | 0.9097 (0.003) | 0.9419 (0.002) |
| 0.15, 0.15 | 30 | 0.8935 (0.003) | 0.9052 (0.003) | 0.8922 (0.003) | 0.9171 (0.003) |
| 0.15, 0.15 | 40 | 0.9000 (0.003) | 0.9072 (0.003) | 0.9038 (0.003) | 0.9125 (0.003) |
| 0.15, 0.15 | 50 | 0.8994 (0.003) | 0.9043 (0.003) | 0.8974 (0.003) | 0.9119 (0.003) |
| 0.15, 0.15 | 60 | 0.8957 (0.003) | 0.8999 (0.003) | 0.8967 (0.003) | 0.9046 (0.003) |
| 0.15, 0.15 | 70 | 0.8940 (0.003) | 0.8993 (0.003) | 0.8942 (0.003) | 0.9010 (0.003) |
| 0.15, 0.15 | 80 | 0.8976 (0.003) | 0.9025 (0.003) | 0.8994 (0.003) | 0.9043 (0.003) |
| 0.15, 0.15 | 90 | 0.9033 (0.003) | 0.9045 (0.003) | 0.9041 (0.003) | 0.9052 (0.003) |
| 0.15, 0.15 | 100 | 0.8982 (0.003) | 0.9025 (0.003) | 0.8981 (0.003) | 0.9033 (0.003) |
| 0.15, 0.15 | 200 | 0.8993 (0.003) | 0.8980 (0.003) | 0.9018 (0.003) | 0.8988 (0.003) |
| 0.15, 0.15 | 507 | 0.9008 (0.003) | 0.8990 (0.003) | 0.8975 (0.003) | 0.8960 (0.003) |
| 0.25, 0.25 | 20 | 0.9220 (0.003) | 0.9182 (0.003) | 0.9525 (0.002) | 0.9543 (0.002) |
| 0.25, 0.25 | 30 | 0.9031 (0.003) | 0.9074 (0.003) | 0.9025 (0.003) | 0.9366 (0.002) |
| 0.25, 0.25 | 40 | 0.8927 (0.003) | 0.9014 (0.003) | 0.8827 (0.003) | 0.9238 (0.003) |
| 0.25, 0.25 | 50 | 0.8969 (0.003) | 0.9001 (0.003) | 0.8990 (0.003) | 0.9146 (0.003) |
| 0.25, 0.25 | 60 | 0.8931 (0.003) | 0.8977 (0.003) | 0.8967 (0.003) | 0.9087 (0.003) |
| 0.25, 0.25 | 70 | 0.9007 (0.003) | 0.8999 (0.003) | 0.8953 (0.003) | 0.9099 (0.003) |
| 0.25, 0.25 | 80 | 0.8931 (0.003) | 0.8967 (0.003) | 0.8951 (0.003) | 0.9019 (0.003) |
| 0.25, 0.25 | 90 | 0.8946 (0.003) | 0.8967 (0.003) | 0.8928 (0.003) | 0.8977 (0.003) |
| 0.25, 0.25 | 100 | 0.8978 (0.003) | 0.9050 (0.003) | 0.8965 (0.003) | 0.9052 (0.003) |
| 0.25, 0.25 | 200 | 0.9057 (0.003) | 0.9074 (0.003) | 0.9071 (0.003) | 0.9052 (0.003) |
| 0.25, 0.25 | 507 | 0.8935 (0.003) | 0.8979 (0.003) | 0.8959 (0.003) | 0.8905 (0.003) |
| 0.1, 0.3 | 20 | 0.9077 (0.003) | 0.8774 (0.003) | 0.9267 (0.003) | 0.9479 (0.002) |
| 0.1, 0.3 | 30 | 0.8910 (0.003) | 0.8604 (0.003) | 0.8959 (0.003) | 0.9330 (0.003) |
| 0.1, 0.3 | 40 | 0.8930 (0.003) | 0.8597 (0.003) | 0.8986 (0.003) | 0.9154 (0.003) |
| 0.1, 0.3 | 50 | 0.8921 (0.003) | 0.8556 (0.004) | 0.8997 (0.003) | 0.9107 (0.003) |
| 0.1, 0.3 | 60 | 0.8932 (0.003) | 0.8550 (0.004) | 0.8965 (0.003) | 0.9074 (0.003) |
| 0.1, 0.3 | 70 | 0.8937 (0.003) | 0.8456 (0.004) | 0.8920 (0.003) | 0.9025 (0.003) |
| 0.1, 0.3 | 80 | 0.8935 (0.003) | 0.8591 (0.003) | 0.8914 (0.003) | 0.9041 (0.003) |
| 0.1, 0.3 | 90 | 0.8972 (0.003) | 0.8599 (0.003) | 0.9020 (0.003) | 0.9003 (0.003) |
| 0.1, 0.3 | 100 | 0.8986 (0.003) | 0.8630 (0.003) | 0.9010 (0.003) | 0.9031 (0.003) |
| 0.1, 0.3 | 200 | 0.9010 (0.003) | 0.8828 (0.003) | 0.8961 (0.003) | 0.8984 (0.003) |
| 0.1, 0.3 | 507 | 0.8946 (0.003) | 0.9481 (0.002) | 0.8943 (0.003) | 0.8941 (0.003) |

Table 6: Coverage for the scenarios with non-inferiority margin$\delta=0.05$, true risk difference $\theta=0.05$ and within-patient correlation $\rho=0.5$; Monte-Carlo standard error in parenthesis

| Probability of missingness per group | Cases | GEE | Hybrid CI | Nam/Tango | Nam/Tango with multiple imputation |
| --- | --- | --- | --- | --- | --- |
| 0, 0 | 20 | 0.8827 (0.003) | 0.9150 (0.003) | 0.8957 (0.003) | 0.8957 (0.003) |
| 0, 0 | 30 | 0.8981 (0.003) | 0.8998 (0.003) | 0.9075 (0.003) | 0.9075 (0.003) |
| 0, 0 | 40 | 0.9000 (0.003) | 0.9053 (0.003) | 0.8931 (0.003) | 0.8931 (0.003) |
| 0, 0 | 50 | 0.9033 (0.003) | 0.9028 (0.003) | 0.8925 (0.003) | 0.8925 (0.003) |
| 0, 0 | 60 | 0.9056 (0.003) | 0.9059 (0.003) | 0.8950 (0.003) | 0.8950 (0.003) |
| 0, 0 | 70 | 0.8915 (0.003) | 0.8993 (0.003) | 0.9076 (0.003) | 0.9076 (0.003) |
| 0, 0 | 80 | 0.8979 (0.003) | 0.8982 (0.003) | 0.8905 (0.003) | 0.8905 (0.003) |
| 0, 0 | 90 | 0.9075 (0.003) | 0.9062 (0.003) | 0.8976 (0.003) | 0.8976 (0.003) |
| 0, 0 | 100 | 0.9043 (0.003) | 0.9043 (0.003) | 0.9012 (0.003) | 0.9012 (0.003) |
| 0, 0 | 200 | 0.8941 (0.003) | 0.8954 (0.003) | 0.8982 (0.003) | 0.8982 (0.003) |
| 0, 0 | 507 | 0.9026 (0.003) | 0.9026 (0.003) | 0.9027 (0.003) | 0.9027 (0.003) |
| 0.15, 0.15 | 20 | 0.9081 (0.003) | 0.9179 (0.003) | 0.9209 (0.003) | 0.9542 (0.002) |
| 0.15, 0.15 | 30 | 0.8879 (0.003) | 0.9030 (0.003) | 0.8909 (0.003) | 0.9286 (0.003) |
| 0.15, 0.15 | 40 | 0.8972 (0.003) | 0.9095 (0.003) | 0.9062 (0.003) | 0.9207 (0.003) |
| 0.15, 0.15 | 50 | 0.8958 (0.003) | 0.9026 (0.003) | 0.8887 (0.003) | 0.9119 (0.003) |
| 0.15, 0.15 | 60 | 0.8933 (0.003) | 0.9033 (0.003) | 0.8882 (0.003) | 0.9063 (0.003) |
| 0.15, 0.15 | 70 | 0.8958 (0.003) | 0.9047 (0.003) | 0.8938 (0.003) | 0.9093 (0.003) |
| 0.15, 0.15 | 80 | 0.9004 (0.003) | 0.9049 (0.003) | 0.8962 (0.003) | 0.9036 (0.003) |
| 0.15, 0.15 | 90 | 0.9025 (0.003) | 0.9091 (0.003) | 0.8991 (0.003) | 0.9083 (0.003) |
| 0.15, 0.15 | 100 | 0.8985 (0.003) | 0.9065 (0.003) | 0.8988 (0.003) | 0.9042 (0.003) |
| 0.15, 0.15 | 200 | 0.8975 (0.003) | 0.9026 (0.003) | 0.8967 (0.003) | 0.9011 (0.003) |
| 0.15, 0.15 | 507 | 0.8956 (0.003) | 0.9004 (0.003) | 0.8954 (0.003) | 0.8981 (0.003) |
| 0.25, 0.25 | 20 | 0.9302 (0.003) | 0.9272 (0.003) | 0.9626 (0.002) | 0.9631 (0.002) |
| 0.25, 0.25 | 30 | 0.8956 (0.003) | 0.9063 (0.003) | 0.9136 (0.003) | 0.9432 (0.002) |
| 0.25, 0.25 | 40 | 0.8927 (0.003) | 0.9006 (0.003) | 0.8930 (0.003) | 0.9320 (0.003) |
| 0.25, 0.25 | 50 | 0.8983 (0.003) | 0.9065 (0.003) | 0.9083 (0.003) | 0.9276 (0.003) |
| 0.25, 0.25 | 60 | 0.8985 (0.003) | 0.9045 (0.003) | 0.9053 (0.003) | 0.9206 (0.003) |
| 0.25, 0.25 | 70 | 0.8960 (0.003) | 0.9037 (0.003) | 0.8949 (0.003) | 0.9136 (0.003) |
| 0.25, 0.25 | 80 | 0.8934 (0.003) | 0.9012 (0.003) | 0.8859 (0.003) | 0.9068 (0.003) |
| 0.25, 0.25 | 90 | 0.9005 (0.003) | 0.9086 (0.003) | 0.8926 (0.003) | 0.9139 (0.003) |
| 0.25, 0.25 | 100 | 0.8978 (0.003) | 0.9057 (0.003) | 0.8950 (0.003) | 0.9056 (0.003) |
| 0.25, 0.25 | 200 | 0.8959 (0.003) | 0.9045 (0.003) | 0.8992 (0.003) | 0.9002 (0.003) |
| 0.25, 0.25 | 507 | 0.9003 (0.003) | 0.9102 (0.003) | 0.8969 (0.003) | 0.8955 (0.003) |
| 0.1, 0.3 | 20 | 0.9207 (0.003) | 0.8824 (0.003) | 0.9450 (0.002) | 0.9628 (0.002) |
| 0.1, 0.3 | 30 | 0.8874 (0.003) | 0.8629 (0.003) | 0.8998 (0.003) | 0.9397 (0.002) |
| 0.1, 0.3 | 40 | 0.8886 (0.003) | 0.8572 (0.003) | 0.9023 (0.003) | 0.9265 (0.003) |
| 0.1, 0.3 | 50 | 0.8966 (0.003) | 0.8650 (0.003) | 0.9031 (0.003) | 0.9208 (0.003) |
| 0.1, 0.3 | 60 | 0.8939 (0.003) | 0.8630 (0.003) | 0.8902 (0.003) | 0.9128 (0.003) |
| 0.1, 0.3 | 70 | 0.8910 (0.003) | 0.8584 (0.003) | 0.8884 (0.003) | 0.9084 (0.003) |
| 0.1, 0.3 | 80 | 0.9000 (0.003) | 0.8701 (0.003) | 0.8973 (0.003) | 0.9123 (0.003) |
| 0.1, 0.3 | 90 | 0.8942 (0.003) | 0.8736 (0.003) | 0.8982 (0.003) | 0.9031 (0.003) |
| 0.1, 0.3 | 100 | 0.8950 (0.003) | 0.8724 (0.003) | 0.8966 (0.003) | 0.9046 (0.003) |
| 0.1, 0.3 | 200 | 0.8968 (0.003) | 0.9103 (0.003) | 0.9002 (0.003) | 0.8987 (0.003) |
| 0.1, 0.3 | 507 | 0.8976 (0.003) | 0.9710 (0.002) | 0.9021 (0.003) | 0.8968 (0.003) |

Table 7: Interval width for the scenarios with non-inferiority margin $\delta=0.05$, true risk difference $\theta=0.05$ and within-patient correlation $\rho=0$

| Probability of missingness per group | Cases | GEE | Hybrid CI | Nam/Tango | Nam/Tango with multiple imputation |
| --- | --- | --- | --- | --- | --- |
| 0, 0 | 20 | 0.4535 | 0.4357 | 0.4371 | 0.4286 |
| 0, 0 | 30 | 0.3641 | 0.3561 | 0.3566 | 0.3514 |
| 0, 0 | 40 | 0.3129 | 0.3081 | 0.3086 | 0.3048 |
| 0, 0 | 50 | 0.2785 | 0.2752 | 0.2757 | 0.2728 |
| 0, 0 | 60 | 0.2535 | 0.2511 | 0.2515 | 0.2492 |
| 0, 0 | 70 | 0.2345 | 0.2326 | 0.2329 | 0.2310 |
| 0, 0 | 80 | 0.2188 | 0.2173 | 0.2176 | 0.2160 |
| 0, 0 | 90 | 0.2063 | 0.2050 | 0.2053 | 0.2040 |
| 0, 0 | 100 | 0.1956 | 0.1945 | 0.1948 | 0.1936 |
| 0, 0 | 200 | 0.1377 | 0.1373 | 0.1374 | 0.1370 |
| 0, 0 | 507 | 0.0863 | 0.0862 | 0.0862 | 0.0861 |
| 0.15, 0.15 | 20 | 0.4917 | 0.4784 | 0.5277 | 0.4763 |
| 0.15, 0.15 | 30 | 0.3927 | 0.3900 | 0.4280 | 0.3859 |
| 0.15, 0.15 | 40 | 0.3378 | 0.3370 | 0.3699 | 0.3346 |
| 0.15, 0.15 | 50 | 0.3017 | 0.3014 | 0.3314 | 0.3000 |
| 0.15, 0.15 | 60 | 0.2748 | 0.2753 | 0.3022 | 0.2735 |
| 0.15, 0.15 | 70 | 0.2537 | 0.2545 | 0.2792 | 0.2531 |
| 0.15, 0.15 | 80 | 0.2374 | 0.2382 | 0.2615 | 0.2370 |
| 0.15, 0.15 | 90 | 0.2231 | 0.2242 | 0.2457 | 0.2229 |
| 0.15, 0.15 | 100 | 0.2116 | 0.2122 | 0.2329 | 0.2116 |
| 0.15, 0.15 | 200 | 0.1493 | 0.1499 | 0.1645 | 0.1499 |
| 0.15, 0.15 | 507 | 0.0935 | 0.0940 | 0.1030 | 0.0940 |
| 0.25, 0.25 | 20 | 0.5232 | 0.5121 | 0.6299 | 0.5141 |
| 0.25, 0.25 | 30 | 0.4171 | 0.4202 | 0.5119 | 0.4150 |
| 0.25, 0.25 | 40 | 0.3588 | 0.3631 | 0.4410 | 0.3593 |
| 0.25, 0.25 | 50 | 0.3198 | 0.3237 | 0.3934 | 0.3208 |
| 0.25, 0.25 | 60 | 0.2907 | 0.2947 | 0.3581 | 0.2917 |
| 0.25, 0.25 | 70 | 0.2693 | 0.2729 | 0.3311 | 0.2707 |
| 0.25, 0.25 | 80 | 0.2517 | 0.2548 | 0.3097 | 0.2532 |
| 0.25, 0.25 | 90 | 0.2374 | 0.2406 | 0.2923 | 0.2389 |
| 0.25, 0.25 | 100 | 0.2249 | 0.2277 | 0.2764 | 0.2263 |
| 0.25, 0.25 | 200 | 0.1586 | 0.1606 | 0.1947 | 0.1599 |
| 0.25, 0.25 | 507 | 0.0995 | 0.1006 | 0.1221 | 0.1005 |
| 0.1, 0.3 | 20 | 0.5114 | 0.4283 | 0.5718 | 0.4974 |
| 0.1, 0.3 | 30 | 0.4085 | 0.3475 | 0.4633 | 0.4037 |
| 0.1, 0.3 | 40 | 0.3529 | 0.3023 | 0.4017 | 0.3511 |
| 0.1, 0.3 | 50 | 0.3139 | 0.2711 | 0.3580 | 0.3130 |
| 0.1, 0.3 | 60 | 0.2857 | 0.2487 | 0.3261 | 0.2857 |
| 0.1, 0.3 | 70 | 0.2644 | 0.2322 | 0.3019 | 0.2650 |
| 0.1, 0.3 | 80 | 0.2474 | 0.2195 | 0.2826 | 0.2479 |
| 0.1, 0.3 | 90 | 0.2327 | 0.2083 | 0.2659 | 0.2333 |
| 0.1, 0.3 | 100 | 0.2210 | 0.1999 | 0.2527 | 0.2220 |
| 0.1, 0.3 | 200 | 0.1557 | 0.1565 | 0.1778 | 0.1567 |
| 0.1, 0.3 | 507 | 0.0976 | 0.1276 | 0.1113 | 0.0984 |

Table 8: Interval width for the scenarios with non-inferiority margin$\delta=0.05$, true risk difference $\theta=0.05$ and within-patient correlation $\rho=0.37$

| Probability of missingness per group | Cases | GEE | Hybrid CI | Nam/Tango | Nam/Tango with multiple imputation |
| --- | --- | --- | --- | --- | --- |
| 0, 0 | 20 | 0.3569 | 0.3533 | 0.3722 | 0.3369 |
| 0, 0 | 30 | 0.2875 | 0.2870 | 0.2977 | 0.2770 |
| 0, 0 | 40 | 0.2478 | 0.2479 | 0.2551 | 0.2412 |
| 0, 0 | 50 | 0.2203 | 0.2204 | 0.2261 | 0.2157 |
| 0, 0 | 60 | 0.2012 | 0.2014 | 0.2059 | 0.1977 |
| 0, 0 | 70 | 0.1863 | 0.1865 | 0.1901 | 0.1836 |
| 0, 0 | 80 | 0.1739 | 0.1741 | 0.1771 | 0.1717 |
| 0, 0 | 90 | 0.1635 | 0.1637 | 0.1663 | 0.1617 |
| 0, 0 | 100 | 0.1553 | 0.1554 | 0.1577 | 0.1537 |
| 0, 0 | 200 | 0.1094 | 0.1094 | 0.1103 | 0.1088 |
| 0, 0 | 507 | 0.0686 | 0.0686 | 0.0688 | 0.0684 |
| 0.15, 0.15 | 20 | 0.4055 | 0.4076 | 0.4621 | 0.3998 |
| 0.15, 0.15 | 30 | 0.3227 | 0.3303 | 0.3656 | 0.3220 |
| 0.15, 0.15 | 40 | 0.2759 | 0.2843 | 0.3099 | 0.2767 |
| 0.15, 0.15 | 50 | 0.2468 | 0.2535 | 0.2750 | 0.2473 |
| 0.15, 0.15 | 60 | 0.2244 | 0.2306 | 0.2489 | 0.2252 |
| 0.15, 0.15 | 70 | 0.2078 | 0.2135 | 0.2294 | 0.2088 |
| 0.15, 0.15 | 80 | 0.1943 | 0.1996 | 0.2137 | 0.1954 |
| 0.15, 0.15 | 90 | 0.1832 | 0.1883 | 0.2011 | 0.1843 |
| 0.15, 0.15 | 100 | 0.1738 | 0.1785 | 0.1902 | 0.1748 |
| 0.15, 0.15 | 200 | 0.1227 | 0.1259 | 0.1327 | 0.1236 |
| 0.15, 0.15 | 507 | 0.0769 | 0.0789 | 0.0825 | 0.0775 |
| 0.25, 0.25 | 20 | 4.707.523 | 0.4515 | 0.5655 | 0.4516 |
| 0.25, 0.25 | 30 | 0.3569 | 0.3670 | 0.4453 | 0.3600 |
| 0.25, 0.25 | 40 | 0.3037 | 0.3151 | 0.3766 | 0.3094 |
| 0.25, 0.25 | 50 | 0.2701 | 0.2816 | 0.3309 | 0.2745 |
| 0.25, 0.25 | 60 | 0.2462 | 0.2571 | 0.2995 | 0.2502 |
| 0.25, 0.25 | 70 | 0.2272 | 0.2374 | 0.2747 | 0.2305 |
| 0.25, 0.25 | 80 | 0.2132 | 0.2222 | 0.2563 | 0.2162 |
| 0.25, 0.25 | 90 | 0.2008 | 0.2094 | 0.2404 | 0.2035 |
| 0.25, 0.25 | 100 | 0.1906 | 0.1985 | 0.2278 | 0.1931 |
| 0.25, 0.25 | 200 | 0.1344 | 0.1399 | 0.1576 | 0.1360 |
| 0.25, 0.25 | 507 | 0.0844 | 0.0879 | 0.0978 | 0.0856 |
| 0.1, 0.3 | 20 | 0.4341 | 0.3706 | 0.5077 | 0.4308 |
| 0.1, 0.3 | 30 | 0.3406 | 0.2960 | 0.3990 | 0.3432 |
| 0.1, 0.3 | 40 | 0.2924 | 0.2577 | 0.3387 | 0.2954 |
| 0.1, 0.3 | 50 | 0.2610 | 0.2328 | 0.2993 | 0.2634 |
| 0.1, 0.3 | 60 | 0.2378 | 0.2149 | 0.2711 | 0.2398 |
| 0.1, 0.3 | 70 | 0.2199 | 0.2014 | 0.2495 | 0.2220 |
| 0.1, 0.3 | 80 | 0.2057 | 0.1919 | 0.2321 | 0.2080 |
| 0.1, 0.3 | 90 | 0.1941 | 0.1833 | 0.2184 | 0.1960 |
| 0.1, 0.3 | 100 | 0.1840 | 0.1763 | 0.2066 | 0.1859 |
| 0.1, 0.3 | 200 | 0.1298 | 0.1438 | 0.1436 | 0.1311 |
| 0.1, 0.3 | 507 | 0.0815 | 0.1230 | 0.0892 | 0.0824 |

Table 9: Interval width for the scenarios with non-inferiority margin$\delta=0.05$, true risk difference $\theta=0.05$ and within-patient correlation $\rho=0.5$

| Probability of missingness per group | Cases | GEE | Hybrid CI | Nam/Tango | Nam/Tango with multiple imputation |
| --- | --- | --- | --- | --- | --- |
| 0, 0 | 20 | 0.3164 | 0.3199 | 0.3471 | 0.2986 |
| 0, 0 | 30 | 0.2546 | 0.2579 | 0.2734 | 0.2454 |
| 0, 0 | 40 | 0.2193 | 0.2220 | 0.2323 | 0.2132 |
| 0, 0 | 50 | 0.1955 | 0.1977 | 0.2053 | 0.1913 |
| 0, 0 | 60 | 0.1787 | 0.1802 | 0.1864 | 0.1755 |
| 0, 0 | 70 | 0.1658 | 0.1671 | 0.1721 | 0.1633 |
| 0, 0 | 80 | 0.1544 | 0.1555 | 0.1598 | 0.1525 |
| 0, 0 | 90 | 0.1455 | 0.1465 | 0.1501 | 0.1439 |
| 0, 0 | 100 | 0.1381 | 0.1389 | 0.1420 | 0.1366 |
| 0, 0 | 200 | 0.0976 | 0.0979 | 0.0990 | 0.0971 |
| 0, 0 | 507 | 0.0610 | 0.0611 | 0.0614 | 0.0609 |
| 0.15, 0.15 | 20 | 0.3725 | 0.3814 | 0.4394 | 0.3717 |
| 0.15, 0.15 | 30 | 0.2902 | 0.3041 | 0.3392 | 0.2941 |
| 0.15, 0.15 | 40 | 0.2482 | 0.2608 | 0.2862 | 0.2521 |
| 0.15, 0.15 | 50 | 0.2214 | 0.2327 | 0.2512 | 0.2244 |
| 0.15, 0.15 | 60 | 0.2022 | 0.2125 | 0.2273 | 0.2048 |
| 0.15, 0.15 | 70 | 0.1868 | 0.1957 | 0.2085 | 0.1891 |
| 0.15, 0.15 | 80 | 0.1748 | 0.1831 | 0.1939 | 0.1771 |
| 0.15, 0.15 | 90 | 0.1649 | 0.1727 | 0.1820 | 0.1665 |
| 0.15, 0.15 | 100 | 0.1563 | 0.1634 | 0.1719 | 0.1581 |
| 0.15, 0.15 | 200 | 0.1104 | 0.1153 | 0.1190 | 0.1116 |
| 0.15, 0.15 | 507 | 0.0694 | 0.0723 | 0.0737 | 0.0699 |
| 0.25, 0.25 | 20 | 0.4308 | 0.4314 | 0.5468 | 0.4315 |
| 0.25, 0.25 | 30 | 0.3298 | 0.3451 | 0.4218 | 0.3391 |
| 0.25, 0.25 | 40 | 0.2792 | 0.2962 | 0.3521 | 0.2885 |
| 0.25, 0.25 | 50 | 0.2470 | 0.2635 | 0.3071 | 0.2549 |
| 0.25, 0.25 | 60 | 0.2249 | 0.2402 | 0.2759 | 0.2318 |
| 0.25, 0.25 | 70 | 0.2077 | 0.2217 | 0.2523 | 0.2133 |
| 0.25, 0.25 | 80 | 0.1946 | 0.2077 | 0.2342 | 0.1995 |
| 0.25, 0.25 | 90 | 0.1831 | 0.1958 | 0.2192 | 0.1876 |
| 0.25, 0.25 | 100 | 0.1736 | 0.1852 | 0.2068 | 0.1777 |
| 0.25, 0.25 | 200 | 0.1227 | 0.1309 | 0.1420 | 0.1251 |
| 0.25, 0.25 | 507 | 0.0771 | 0.0822 | 0.0876 | 0.0784 |
| 0.1, 0.3 | 20 | 0.4048 | 0.3484 | 0.4870 | 0.4068 |
| 0.1, 0.3 | 30 | 0.3117 | 0.2759 | 0.3759 | 0.3189 |
| 0.1, 0.3 | 40 | 0.2634 | 0.2375 | 0.3125 | 0.2704 |
| 0.1, 0.3 | 50 | 0.2356 | 0.2156 | 0.2751 | 0.2413 |
| 0.1, 0.3 | 60 | 0.2149 | 0.1999 | 0.2477 | 0.2197 |
| 0.1, 0.3 | 70 | 0.1985 | 0.1876 | 0.2273 | 0.2030 |
| 0.1, 0.3 | 80 | 0.1860 | 0.1791 | 0.2113 | 0.1899 |
| 0.1, 0.3 | 90 | 0.1754 | 0.1722 | 0.1980 | 0.1788 |
| 0.1, 0.3 | 100 | 0.1667 | 0.1663 | 0.1874 | 0.1695 |
| 0.1, 0.3 | 200 | 0.1176 | 0.1383 | 0.1292 | 0.1194 |
| 0.1, 0.3 | 507 | 0.0739 | 0.1211 | 0.0798 | 0.0748 |

Table 10: Power for the scenarios with non-inferiority margin $\delta=0.05$, true risk difference $\theta=0$ and within-patient correlation $\rho=0$; Monte-Carlo standard error in parenthesis

| Probability of missingness per group | Cases | GEE | Hybrid CI | Nam/Tango | Nam/Tango with multiple imputation |
| --- | --- | --- | --- | --- | --- |
| 0, 0 | 20 | 0.1041 (0.003) | 0.0943 (0.003) | 0.1037 (0.003) | 0.1037 (0.003) |
| 0, 0 | 30 | 0.1299 (0.003) | 0.1082 (0.003) | 0.1107 (0.003) | 0.1107 (0.003) |
| 0, 0 | 40 | 0.1370 (0.003) | 0.1335 (0.003) | 0.1368 (0.003) | 0.1368 (0.003) |
| 0, 0 | 50 | 0.1573 (0.004) | 0.1554 (0.004) | 0.1575 (0.004) | 0.1575 (0.004) |
| 0, 0 | 60 | 0.1568 (0.004) | 0.1581 (0.004) | 0.1629 (0.004) | 0.1629 (0.004) |
| 0, 0 | 70 | 0.1834 (0.004) | 0.1731 (0.004) | 0.1809 (0.004) | 0.1809 (0.004) |
| 0, 0 | 80 | 0.1931 (0.004) | 0.1873 (0.004) | 0.1931 (0.004) | 0.1931 (0.004) |
| 0, 0 | 90 | 0.2038 (0.004) | 0.2049 (0.004) | 0.2043 (0.004) | 0.2043 (0.004) |
| 0, 0 | 100 | 0.2239 (0.004) | 0.2219 (0.004) | 0.2219 (0.004) | 0.2219 (0.004) |
| 0, 0 | 200 | 0.3615 (0.005) | 0.3599 (0.005) | 0.3615 (0.005) | 0.3615 (0.005) |
| 0, 0 | 507 | 0.6393 (0.005) | 0.6393 (0.005) | 0.6393 (0.005) | 0.6393 (0.005) |
| 0.15, 0.15 | 20 | 0.0959 (0.003) | 0.0826 (0.003) | 0.0803 (0.003) | 0.0735 (0.003) |
| 0.15, 0.15 | 30 | 0.1192 (0.003) | 0.1095 (0.003) | 0.1068 (0.003) | 0.1090 (0.003) |
| 0.15, 0.15 | 40 | 0.1262 (0.003) | 0.1179 (0.003) | 0.1126 (0.003) | 0.1161 (0.003) |
| 0.15, 0.15 | 50 | 0.1408 (0.003) | 0.1361 (0.003) | 0.1277 (0.003) | 0.1318 (0.003) |
| 0.15, 0.15 | 60 | 0.1526 (0.004) | 0.1505 (0.004) | 0.1339 (0.003) | 0.1465 (0.004) |
| 0.15, 0.15 | 70 | 0.1708 (0.004) | 0.1671 (0.004) | 0.1546 (0.004) | 0.1665 (0.004) |
| 0.15, 0.15 | 80 | 0.1756 (0.004) | 0.1788 (0.004) | 0.1547 (0.004) | 0.1711 (0.004) |
| 0.15, 0.15 | 90 | 0.1973 (0.004) | 0.1946 (0.004) | 0.1725 (0.004) | 0.1926 (0.004) |
| 0.15, 0.15 | 100 | 0.2049 (0.004) | 0.2006 (0.004) | 0.1851 (0.004) | 0.1994 (0.004) |
| 0.15, 0.15 | 200 | 0.3101 (0.005) | 0.3068 (0.005) | 0.2795 (0.004) | 0.3071 (0.005) |
| 0.15, 0.15 | 507 | 0.5774 (0.005) | 0.5742 (0.005) | 0.5088 (0.005) | 0.5734 (0.005) |
| 0.25, 0.25 | 20 | 0.0803 (0.003) | 0.0721 (0.003) | 0.0554 (0.002) | 0.0558 (0.002) |
| 0.25, 0.25 | 30 | 0.1134 (0.003) | 0.1031 (0.003) | 0.0930 (0.003) | 0.0890 (0.003) |
| 0.25, 0.25 | 40 | 0.1255 (0.003) | 0.1223 (0.003) | 0.1042 (0.003) | 0.1083 (0.003) |
| 0.25, 0.25 | 50 | 0.1418 (0.003) | 0.1365 (0.003) | 0.1123 (0.003) | 0.1277 (0.003) |
| 0.25, 0.25 | 60 | 0.1416 (0.003) | 0.1385 (0.003) | 0.1107 (0.003) | 0.1286 (0.003) |
| 0.25, 0.25 | 70 | 0.1562 (0.004) | 0.1549 (0.004) | 0.1276 (0.003) | 0.1485 (0.004) |
| 0.25, 0.25 | 80 | 0.1654 (0.004) | 0.1636 (0.004) | 0.1371 (0.003) | 0.1552 (0.004) |
| 0.25, 0.25 | 90 | 0.1882 (0.004) | 0.1866 (0.004) | 0.1523 (0.004) | 0.1797 (0.004) |
| 0.25, 0.25 | 100 | 0.1877 (0.004) | 0.1876 (0.004) | 0.1415 (0.003) | 0.1817 (0.004) |
| 0.25, 0.25 | 200 | 0.2905 (0.005) | 0.2950 (0.005) | 0.2254 (0.004) | 0.2858 (0.005) |
| 0.25, 0.25 | 507 | 0.5233 (0.005) | 0.5166 (0.005) | 0.3992 (0.005) | 0.5154 (0.005) |
| 0.1, 0.3 | 20 | 0.0851 (0.003) | 0.1280 (0.003) | 0.0579 (0.002) | 0.0479 (0.002) |
| 0.1, 0.3 | 30 | 0.1194 (0.003) | 0.1842 (0.004) | 0.0952 (0.003) | 0.0803 (0.003) |
| 0.1, 0.3 | 40 | 0.1280 (0.003) | 0.2230 (0.004) | 0.1037 (0.003) | 0.0945 (0.003) |
| 0.1, 0.3 | 50 | 0.1460 (0.004) | 0.2584 (0.004) | 0.1167 (0.003) | 0.1159 (0.003) |
| 0.1, 0.3 | 60 | 0.1530 (0.004) | 0.2864 (0.005) | 0.1258 (0.003) | 0.1286 (0.003) |
| 0.1, 0.3 | 70 | 0.1728 (0.004) | 0.3239 (0.005) | 0.1390 (0.003) | 0.1459 (0.004) |
| 0.1, 0.3 | 80 | 0.1878 (0.004) | 0.3479 (0.005) | 0.1509 (0.004) | 0.1623 (0.004) |
| 0.1, 0.3 | 90 | 0.1888 (0.004) | 0.3673 (0.005) | 0.1584 (0.004) | 0.1632 (0.004) |
| 0.1, 0.3 | 100 | 0.2056 (0.004) | 0.3867 (0.005) | 0.1717 (0.004) | 0.1864 (0.004) |
| 0.1, 0.3 | 200 | 0.3035 (0.005) | 0.5395 (0.005) | 0.2503 (0.004) | 0.2876 (0.005) |
| 0.1, 0.3 | 507 | 0.5502 (0.005) | 0.7003 (0.005) | 0.4612 (0.005) | 0.5329 (0.005) |

Table 11: Power for the scenarios with non-inferiority margin $\delta=0.05$, true risk difference $\theta=0$ and within-patient correlation $\rho=0.37$; Monte-Carlo standard error in parenthesis

| Probability of missingness per group | Cases | GEE | Hybrid CI | Nam/Tango | Nam/Tango with multiple imputation |
| --- | --- | --- | --- | --- | --- |
| 0, 0 | 20 | 0.1629 (0.004) | 0.1250 (0.003) | 0.1223 (0.003) | 0.1223 (0.003) |
| 0, 0 | 30 | 0.1861 (0.004) | 0.1433 (0.004) | 0.1151 (0.003) | 0.1151 (0.003) |
| 0, 0 | 40 | 0.1651 (0.004) | 0.1632 (0.004) | 0.1591 (0.004) | 0.1591 (0.004) |
| 0, 0 | 50 | 0.1865 (0.004) | 0.1872 (0.004) | 0.1850 (0.004) | 0.1850 (0.004) |
| 0, 0 | 60 | 0.2110 (0.004) | 0.2094 (0.004) | 0.2085 (0.004) | 0.2085 (0.004) |
| 0, 0 | 70 | 0.2527 (0.004) | 0.2320 (0.004) | 0.2189 (0.004) | 0.2189 (0.004) |
| 0, 0 | 80 | 0.2679 (0.004) | 0.2615 (0.004) | 0.2679 (0.004) | 0.2679 (0.004) |
| 0, 0 | 90 | 0.2670 (0.004) | 0.2673 (0.004) | 0.2669 (0.004) | 0.2669 (0.004) |
| 0, 0 | 100 | 0.2976 (0.005) | 0.2916 (0.005) | 0.2869 (0.005) | 0.2869 (0.005) |
| 0, 0 | 200 | 0.4850 (0.005) | 0.4783 (0.005) | 0.4756 (0.005) | 0.4756 (0.005) |
| 0, 0 | 507 | 0.8097 (0.004) | 0.8093 (0.004) | 0.8054 (0.004) | 0.8054 (0.004) |
| 0.15, 0.15 | 20 | 0.1175 (0.003) | 0.1052 (0.003) | 0.0989 (0.003) | 0.0679 (0.003) |
| 0.15, 0.15 | 30 | 0.1492 (0.004) | 0.1379 (0.003) | 0.1266 (0.003) | 0.1083 (0.003) |
| 0.15, 0.15 | 40 | 0.1631 (0.004) | 0.1518 (0.004) | 0.1279 (0.003) | 0.1395 (0.003) |
| 0.15, 0.15 | 50 | 0.1752 (0.004) | 0.1657 (0.004) | 0.1519 (0.004) | 0.1547 (0.004) |
| 0.15, 0.15 | 60 | 0.1992 (0.004) | 0.1846 (0.004) | 0.1698 (0.004) | 0.1801 (0.004) |
| 0.15, 0.15 | 70 | 0.2175 (0.004) | 0.2055 (0.004) | 0.1934 (0.004) | 0.1984 (0.004) |
| 0.15, 0.15 | 80 | 0.2298 (0.004) | 0.2168 (0.004) | 0.1962 (0.004) | 0.2111 (0.004) |
| 0.15, 0.15 | 90 | 0.2481 (0.004) | 0.2345 (0.004) | 0.2153 (0.004) | 0.2342 (0.004) |
| 0.15, 0.15 | 100 | 0.2629 (0.004) | 0.2527 (0.004) | 0.2320 (0.004) | 0.2489 (0.004) |
| 0.15, 0.15 | 200 | 0.4037 (0.005) | 0.3898 (0.005) | 0.3564 (0.005) | 0.3894 (0.005) |
| 0.15, 0.15 | 507 | 0.7309 (0.004) | 0.7098 (0.005) | 0.6771 (0.005) | 0.7137 (0.005) |
| 0.25, 0.25 | 20 | 0.0853 (0.003) | 0.0892 (0.003) | 0.0505 (0.002) | 0.0497 (0.002) |
| 0.25, 0.25 | 30 | 0.1388 (0.003) | 0.1239 (0.003) | 0.1106 (0.003) | 0.0894 (0.003) |
| 0.25, 0.25 | 40 | 0.1612 (0.004) | 0.1490 (0.004) | 0.1244 (0.003) | 0.1199 (0.003) |
| 0.25, 0.25 | 50 | 0.1668 (0.004) | 0.1610 (0.004) | 0.1257 (0.003) | 0.1345 (0.003) |
| 0.25, 0.25 | 60 | 0.1796 (0.004) | 0.1713 (0.004) | 0.1381 (0.003) | 0.1554 (0.004) |
| 0.25, 0.25 | 70 | 0.1949 (0.004) | 0.1880 (0.004) | 0.1555 (0.004) | 0.1744 (0.004) |
| 0.25, 0.25 | 80 | 0.2050 (0.004) | 0.1963 (0.004) | 0.1650 (0.004) | 0.1881 (0.004) |
| 0.25, 0.25 | 90 | 0.2223 (0.004) | 0.2094 (0.004) | 0.1743 (0.004) | 0.2046 (0.004) |
| 0.25, 0.25 | 100 | 0.2335 (0.004) | 0.2223 (0.004) | 0.1882 (0.004) | 0.2145 (0.004) |
| 0.25, 0.25 | 200 | 0.3574 (0.005) | 0.3385 (0.005) | 0.2899 (0.005) | 0.3419 (0.005) |
| 0.25, 0.25 | 507 | 0.6583 (0.005) | 0.6246 (0.005) | 0.5526 (0.005) | 0.6420 (0.005) |
| 0.1, 0.3 | 20 | 0.1197 (0.003) | 0.1672 (0.004) | 0.0769 (0.003) | 0.0487 (0.002) |
| 0.1, 0.3 | 30 | 0.1550 (0.004) | 0.2266 (0.004) | 0.1146 (0.003) | 0.0808 (0.003) |
| 0.1, 0.3 | 40 | 0.1728 (0.004) | 0.2633 (0.004) | 0.1236 (0.003) | 0.1117 (0.003) |
| 0.1, 0.3 | 50 | 0.1850 (0.004) | 0.2916 (0.005) | 0.1293 (0.003) | 0.1317 (0.003) |
| 0.1, 0.3 | 60 | 0.2024 (0.004) | 0.3328 (0.005) | 0.1595 (0.004) | 0.1526 (0.004) |
| 0.1, 0.3 | 70 | 0.2172 (0.004) | 0.3589 (0.005) | 0.1706 (0.004) | 0.1709 (0.004) |
| 0.1, 0.3 | 80 | 0.2270 (0.004) | 0.3725 (0.005) | 0.1817 (0.004) | 0.1879 (0.004) |
| 0.1, 0.3 | 90 | 0.2382 (0.004) | 0.3974 (0.005) | 0.1912 (0.004) | 0.1966 (0.004) |
| 0.1, 0.3 | 100 | 0.2611 (0.004) | 0.4227 (0.005) | 0.2143 (0.004) | 0.2190 (0.004) |
| 0.1, 0.3 | 200 | 0.3843 (0.005) | 0.5445 (0.005) | 0.3273 (0.005) | 0.3561 (0.005) |
| 0.1, 0.3 | 507 | 0.6958 (0.005) | 0.6810 (0.005) | 0.6223 (0.005) | 0.6662 (0.005) |

Table 12: Power for the scenarios with non-inferiority margin $\delta=0.05$, true risk difference $\theta=0$ and within-patient correlation $\rho=0.5$; Monte-Carlo standard error in parenthesis

| Probability of missingness per group | Cases | GEE | Hybrid CI | Nam/Tango | Nam/Tango with multiple imputation |
| --- | --- | --- | --- | --- | --- |
| 0, 0 | 20 | 0.2026 (0.004) | 0.1372 (0.003) | 0.1208 (0.003) | 0.1208 (0.003) |
| 0, 0 | 30 | 0.2037 (0.004) | 0.1602 (0.004) | 0.1204 (0.003) | 0.1204 (0.003) |
| 0, 0 | 40 | 0.2013 (0.004) | 0.1795 (0.004) | 0.1756 (0.004) | 0.1756 (0.004) |
| 0, 0 | 50 | 0.2280 (0.004) | 0.2240 (0.004) | 0.2153 (0.004) | 0.2153 (0.004) |
| 0, 0 | 60 | 0.2574 (0.004) | 0.2501 (0.004) | 0.2441 (0.004) | 0.2441 (0.004) |
| 0, 0 | 70 | 0.2958 (0.005) | 0.2706 (0.004) | 0.2445 (0.004) | 0.2445 (0.004) |
| 0, 0 | 80 | 0.2915 (0.005) | 0.2872 (0.005) | 0.2905 (0.005) | 0.2905 (0.005) |
| 0, 0 | 90 | 0.3225 (0.005) | 0.3216 (0.005) | 0.3206 (0.005) | 0.3206 (0.005) |
| 0, 0 | 100 | 0.3498 (0.005) | 0.3477 (0.005) | 0.3442 (0.005) | 0.3442 (0.005) |
| 0, 0 | 200 | 0.5529 (0.005) | 0.5426 (0.005) | 0.5329 (0.005) | 0.5329 (0.005) |
| 0, 0 | 507 | 0.8717 (0.003) | 0.8703 (0.003) | 0.8673 (0.003) | 0.8673 (0.003) |
| 0.15, 0.15 | 20 | 0.1339 (0.003) | 0.1109 (0.003) | 0.1025 (0.003) | 0.0648 (0.002) |
| 0.15, 0.15 | 30 | 0.1779 (0.004) | 0.1488 (0.004) | 0.1306 (0.003) | 0.1091 (0.003) |
| 0.15, 0.15 | 40 | 0.1871 (0.004) | 0.1679 (0.004) | 0.1311 (0.003) | 0.1459 (0.004) |
| 0.15, 0.15 | 50 | 0.2027 (0.004) | 0.1887 (0.004) | 0.1713 (0.004) | 0.1669 (0.004) |
| 0.15, 0.15 | 60 | 0.2227 (0.004) | 0.2038 (0.004) | 0.1784 (0.004) | 0.1869 (0.004) |
| 0.15, 0.15 | 70 | 0.2472 (0.004) | 0.2227 (0.004) | 0.2045 (0.004) | 0.2149 (0.004) |
| 0.15, 0.15 | 80 | 0.2615 (0.004) | 0.2393 (0.004) | 0.2195 (0.004) | 0.2342 (0.004) |
| 0.15, 0.15 | 90 | 0.2811 (0.004) | 0.2653 (0.004) | 0.2408 (0.004) | 0.2561 (0.004) |
| 0.15, 0.15 | 100 | 0.3036 (0.005) | 0.2853 (0.005) | 0.2595 (0.004) | 0.2798 (0.004) |
| 0.15, 0.15 | 200 | 0.4690 (0.005) | 0.4419 (0.005) | 0.4205 (0.005) | 0.4478 (0.005) |
| 0.15, 0.15 | 507 | 0.7941 (0.004) | 0.7722 (0.004) | 0.7456 (0.004) | 0.7820 (0.004) |
| 0.25, 0.25 | 20 | 0.0949 (0.003) | 0.0983 (0.003) | 0.0461 (0.002) | 0.0477 (0.002) |
| 0.25, 0.25 | 30 | 0.1582 (0.004) | 0.1350 (0.003) | 0.1100 (0.003) | 0.0834 (0.003) |
| 0.25, 0.25 | 40 | 0.1796 (0.004) | 0.1611 (0.004) | 0.1249 (0.003) | 0.1140 (0.003) |
| 0.25, 0.25 | 50 | 0.1903 (0.004) | 0.1761 (0.004) | 0.1304 (0.003) | 0.1344 (0.003) |
| 0.25, 0.25 | 60 | 0.2027 (0.004) | 0.1901 (0.004) | 0.1412 (0.003) | 0.1571 (0.004) |
| 0.25, 0.25 | 70 | 0.2122 (0.004) | 0.1966 (0.004) | 0.1663 (0.004) | 0.1751 (0.004) |
| 0.25, 0.25 | 80 | 0.2310 (0.004) | 0.2098 (0.004) | 0.1725 (0.004) | 0.1955 (0.004) |
| 0.25, 0.25 | 90 | 0.2464 (0.004) | 0.2230 (0.004) | 0.1901 (0.004) | 0.2156 (0.004) |
| 0.25, 0.25 | 100 | 0.2627 (0.004) | 0.2409 (0.004) | 0.2087 (0.004) | 0.2331 (0.004) |
| 0.25, 0.25 | 200 | 0.4033 (0.005) | 0.3682 (0.005) | 0.3283 (0.005) | 0.3824 (0.005) |
| 0.25, 0.25 | 507 | 0.7152 (0.005) | 0.6745 (0.005) | 0.6216 (0.005) | 0.6963 (0.005) |
| 0.1, 0.3 | 20 | 0.1240 (0.003) | 0.1822 (0.004) | 0.0745 (0.003) | 0.0423 (0.002) |
| 0.1, 0.3 | 30 | 0.1789 (0.004) | 0.2399 (0.004) | 0.1140 (0.003) | 0.0744 (0.003) |
| 0.1, 0.3 | 40 | 0.1918 (0.004) | 0.2728 (0.004) | 0.1233 (0.003) | 0.1102 (0.003) |
| 0.1, 0.3 | 50 | 0.2049 (0.004) | 0.3053 (0.005) | 0.1419 (0.003) | 0.1385 (0.003) |
| 0.1, 0.3 | 60 | 0.2229 (0.004) | 0.3421 (0.005) | 0.1736 (0.004) | 0.1556 (0.004) |
| 0.1, 0.3 | 70 | 0.2419 (0.004) | 0.3682 (0.005) | 0.1881 (0.004) | 0.1809 (0.004) |
| 0.1, 0.3 | 80 | 0.2543 (0.004) | 0.3848 (0.005) | 0.1994 (0.004) | 0.1948 (0.004) |
| 0.1, 0.3 | 90 | 0.2731 (0.004) | 0.4075 (0.005) | 0.2188 (0.004) | 0.2164 (0.004) |
| 0.1, 0.3 | 100 | 0.2841 (0.005) | 0.4200 (0.005) | 0.2298 (0.004) | 0.2320 (0.004) |
| 0.1, 0.3 | 200 | 0.4335 (0.005) | 0.5445 (0.005) | 0.3768 (0.005) | 0.3905 (0.005) |
| 0.1, 0.3 | 507 | 0.7531 (0.004) | 0.6625 (0.005) | 0.6940 (0.005) | 0.7242 (0.004) |

Table 13: Coverage for the scenarios with non-inferiority margin$\delta=0.05$, true risk difference $\theta=0$ and within-patient correlation $\rho=0$; Monte-Carlo standard error in parenthesis

| Probability of missingness per group | Cases | GEE | Hybrid CI | Nam/Tango | Nam/Tango with multiple imputation |
| --- | --- | --- | --- | --- | --- |
| 0, 0 | 20 | 0.9213 (0.003) | 0.9303 (0.003) | 0.9213 (0.003) | 0.9104 (0.003) |
| 0, 0 | 30 | 0.8938 (0.003) | 0.9149 (0.003) | 0.8938 (0.003) | 0.9016 (0.003) |
| 0, 0 | 40 | 0.8942 (0.003) | 0.9061 (0.003) | 0.8942 (0.003) | 0.8981 (0.003) |
| 0, 0 | 50 | 0.8944 (0.003) | 0.8974 (0.003) | 0.8944 (0.003) | 0.8945 (0.003) |
| 0, 0 | 60 | 0.8964 (0.003) | 0.8998 (0.003) | 0.8964 (0.003) | 0.8945 (0.003) |
| 0, 0 | 70 | 0.9063 (0.003) | 0.9068 (0.003) | 0.9063 (0.003) | 0.9032 (0.003) |
| 0, 0 | 80 | 0.9038 (0.003) | 0.9057 (0.003) | 0.9038 (0.003) | 0.8994 (0.003) |
| 0, 0 | 90 | 0.9013 (0.003) | 0.9029 (0.003) | 0.9013 (0.003) | 0.9018 (0.003) |
| 0, 0 | 100 | 0.8969 (0.003) | 0.8999 (0.003) | 0.8969 (0.003) | 0.9013 (0.003) |
| 0, 0 | 200 | 0.8992 (0.003) | 0.9009 (0.003) | 0.8992 (0.003) | 0.8999 (0.003) |
| 0, 0 | 507 | 0.8984 (0.003) | 0.8984 (0.003) | 0.8984 (0.003) | 0.8995 (0.003) |
| 0.15, 0.15 | 20 | 0.9187 (0.003) | 0.9318 (0.003) | 0.9180 (0.003) | 0.9344 (0.002) |
| 0.15, 0.15 | 30 | 0.9012 (0.003) | 0.9144 (0.003) | 0.9090 (0.003) | 0.9141 (0.003) |
| 0.15, 0.15 | 40 | 0.8999 (0.003) | 0.9083 (0.003) | 0.8976 (0.003) | 0.9092 (0.003) |
| 0.15, 0.15 | 50 | 0.8982 (0.003) | 0.9077 (0.003) | 0.8970 (0.003) | 0.9063 (0.003) |
| 0.15, 0.15 | 60 | 0.9000 (0.003) | 0.9057 (0.003) | 0.9006 (0.003) | 0.9058 (0.003) |
| 0.15, 0.15 | 70 | 0.8991 (0.003) | 0.9068 (0.003) | 0.9056 (0.003) | 0.9045 (0.003) |
| 0.15, 0.15 | 80 | 0.8989 (0.003) | 0.9055 (0.003) | 0.9042 (0.003) | 0.9026 (0.003) |
| 0.15, 0.15 | 90 | 0.8966 (0.003) | 0.9000 (0.003) | 0.9026 (0.003) | 0.9020 (0.003) |
| 0.15, 0.15 | 100 | 0.8977 (0.003) | 0.9016 (0.003) | 0.9012 (0.003) | 0.9030 (0.003) |
| 0.15, 0.15 | 200 | 0.9021 (0.003) | 0.9010 (0.003) | 0.9041 (0.003) | 0.9026 (0.003) |
| 0.15, 0.15 | 507 | 0.8999 (0.003) | 0.9001 (0.003) | 0.9014 (0.003) | 0.8992 (0.003) |
| 0.25, 0.25 | 20 | 0.9323 (0.003) | 0.9451 (0.002) | 0.9193 (0.003) | 0.9530 (0.002) |
| 0.25, 0.25 | 30 | 0.9022 (0.003) | 0.9165 (0.003) | 0.9029 (0.003) | 0.9278 (0.003) |
| 0.25, 0.25 | 40 | 0.8982 (0.003) | 0.9074 (0.003) | 0.9086 (0.003) | 0.9157 (0.003) |
| 0.25, 0.25 | 50 | 0.8899 (0.003) | 0.9031 (0.003) | 0.8938 (0.003) | 0.9073 (0.003) |
| 0.25, 0.25 | 60 | 0.8969 (0.003) | 0.9034 (0.003) | 0.8960 (0.003) | 0.9094 (0.003) |
| 0.25, 0.25 | 70 | 0.8980 (0.003) | 0.9048 (0.003) | 0.8935 (0.003) | 0.9059 (0.003) |
| 0.25, 0.25 | 80 | 0.8925 (0.003) | 0.8952 (0.003) | 0.8949 (0.003) | 0.9006 (0.003) |
| 0.25, 0.25 | 90 | 0.8924 (0.003) | 0.8992 (0.003) | 0.8978 (0.003) | 0.9016 (0.003) |
| 0.25, 0.25 | 100 | 0.8987 (0.003) | 0.9006 (0.003) | 0.9059 (0.003) | 0.9068 (0.003) |
| 0.25, 0.25 | 200 | 0.8984 (0.003) | 0.8986 (0.003) | 0.9020 (0.003) | 0.8993 (0.003) |
| 0.25, 0.25 | 507 | 0.8968 (0.003) | 0.8954 (0.003) | 0.8954 (0.003) | 0.8982 (0.003) |
| 0.1, 0.3 | 20 | 0.9241 (0.003) | 0.9154 (0.003) | 0.9136 (0.003) | 0.9406 (0.002) |
| 0.1, 0.3 | 30 | 0.9033 (0.003) | 0.8877 (0.003) | 0.9124 (0.003) | 0.9208 (0.003) |
| 0.1, 0.3 | 40 | 0.8957 (0.003) | 0.8744 (0.003) | 0.9015 (0.003) | 0.9109 (0.003) |
| 0.1, 0.3 | 50 | 0.8979 (0.003) | 0.8676 (0.003) | 0.8977 (0.003) | 0.9088 (0.003) |
| 0.1, 0.3 | 60 | 0.8977 (0.003) | 0.8672 (0.003) | 0.8991 (0.003) | 0.9070 (0.003) |
| 0.1, 0.3 | 70 | 0.8985 (0.003) | 0.8553 (0.004) | 0.9021 (0.003) | 0.9064 (0.003) |
| 0.1, 0.3 | 80 | 0.8963 (0.003) | 0.8538 (0.004) | 0.8994 (0.003) | 0.9029 (0.003) |
| 0.1, 0.3 | 90 | 0.8932 (0.003) | 0.8538 (0.004) | 0.8957 (0.003) | 0.8975 (0.003) |
| 0.1, 0.3 | 100 | 0.8961 (0.003) | 0.8456 (0.004) | 0.9002 (0.003) | 0.8989 (0.003) |
| 0.1, 0.3 | 200 | 0.8947 (0.003) | 0.8408 (0.004) | 0.8973 (0.003) | 0.8944 (0.003) |
| 0.1, 0.3 | 507 | 0.9015 (0.003) | 0.8839 (0.003) | 0.9008 (0.003) | 0.9017 (0.003) |

Table 14: Coverage for the scenarios with non-inferiority margin $\delta=0.05$, true risk difference $\theta=0$ and within-patient correlation $\rho=0.37$; Monte-Carlo standard error in parenthesis

| Probability of missingness per group | Cases | GEE | Hybrid CI | Nam/Tango | Nam/Tango with multiple imputation |
| --- | --- | --- | --- | --- | --- |
| 0, 0 | 20 | 0.9026 (0.003) | 0.9111 (0.003) | 0.9062 (0.003) | 0.8905 (0.003) |
| 0, 0 | 30 | 0.9066 (0.003) | 0.9168 (0.003) | 0.9068 (0.003) | 0.8960 (0.003) |
| 0, 0 | 40 | 0.9030 (0.003) | 0.9132 (0.003) | 0.9030 (0.003) | 0.9050 (0.003) |
| 0, 0 | 50 | 0.8907 (0.003) | 0.9055 (0.003) | 0.8907 (0.003) | 0.8970 (0.003) |
| 0, 0 | 60 | 0.8961 (0.003) | 0.9061 (0.003) | 0.8961 (0.003) | 0.9085 (0.003) |
| 0, 0 | 70 | 0.8949 (0.003) | 0.9003 (0.003) | 0.8949 (0.003) | 0.8997 (0.003) |
| 0, 0 | 80 | 0.9030 (0.003) | 0.9052 (0.003) | 0.9030 (0.003) | 0.9061 (0.003) |
| 0, 0 | 90 | 0.9005 (0.003) | 0.9014 (0.003) | 0.9005 (0.003) | 0.9062 (0.003) |
| 0, 0 | 100 | 0.9065 (0.003) | 0.9072 (0.003) | 0.9065 (0.003) | 0.9116 (0.003) |
| 0, 0 | 200 | 0.9012 (0.003) | 0.9012 (0.003) | 0.9012 (0.003) | 0.9056 (0.003) |
| 0, 0 | 507 | 0.9016 (0.003) | 0.9016 (0.003) | 0.9016 (0.003) | 0.9066 (0.003) |
| 0.15, 0.15 | 20 | 0.9246 (0.003) | 0.9301 (0.003) | 0.9129 (0.003) | 0.9407 (0.002) |
| 0.15, 0.15 | 30 | 0.9060 (0.003) | 0.9151 (0.003) | 0.8971 (0.003) | 0.9258 (0.003) |
| 0.15, 0.15 | 40 | 0.8905 (0.003) | 0.8984 (0.003) | 0.8973 (0.003) | 0.9079 (0.003) |
| 0.15, 0.15 | 50 | 0.9000 (0.003) | 0.9065 (0.003) | 0.9009 (0.003) | 0.9171 (0.003) |
| 0.15, 0.15 | 60 | 0.8958 (0.003) | 0.9052 (0.003) | 0.8941 (0.003) | 0.9095 (0.003) |
| 0.15, 0.15 | 70 | 0.8993 (0.003) | 0.9065 (0.003) | 0.8967 (0.003) | 0.9129 (0.003) |
| 0.15, 0.15 | 80 | 0.9003 (0.003) | 0.9065 (0.003) | 0.8991 (0.003) | 0.9106 (0.003) |
| 0.15, 0.15 | 90 | 0.8980 (0.003) | 0.9048 (0.003) | 0.8964 (0.003) | 0.9097 (0.003) |
| 0.15, 0.15 | 100 | 0.8967 (0.003) | 0.9028 (0.003) | 0.8983 (0.003) | 0.9066 (0.003) |
| 0.15, 0.15 | 200 | 0.9016 (0.003) | 0.9029 (0.003) | 0.9004 (0.003) | 0.9064 (0.003) |
| 0.15, 0.15 | 507 | 0.9074 (0.003) | 0.9089 (0.003) | 0.9075 (0.003) | 0.9099 (0.003) |
| 0.25, 0.25 | 20 | 0.9432 (0.002) | 0.9408 (0.002) | 0.9363 (0.002) | 0.9672 (0.002) |
| 0.25, 0.25 | 30 | 0.9112 (0.003) | 0.9183 (0.003) | 0.9026 (0.003) | 0.9379 (0.002) |
| 0.25, 0.25 | 40 | 0.8987 (0.003) | 0.9090 (0.003) | 0.8992 (0.003) | 0.9279 (0.003) |
| 0.25, 0.25 | 50 | 0.8967 (0.003) | 0.9068 (0.003) | 0.8969 (0.003) | 0.9197 (0.003) |
| 0.25, 0.25 | 60 | 0.8953 (0.003) | 0.9039 (0.003) | 0.8999 (0.003) | 0.9171 (0.003) |
| 0.25, 0.25 | 70 | 0.8980 (0.003) | 0.9019 (0.003) | 0.9029 (0.003) | 0.9136 (0.003) |
| 0.25, 0.25 | 80 | 0.8963 (0.003) | 0.8996 (0.003) | 0.8992 (0.003) | 0.9124 (0.003) |
| 0.25, 0.25 | 90 | 0.8987 (0.003) | 0.9085 (0.003) | 0.9017 (0.003) | 0.9121 (0.003) |
| 0.25, 0.25 | 100 | 0.9001 (0.003) | 0.9061 (0.003) | 0.8882 (0.003) | 0.9065 (0.003) |
| 0.25, 0.25 | 200 | 0.8937 (0.003) | 0.8987 (0.003) | 0.8994 (0.003) | 0.8983 (0.003) |
| 0.25, 0.25 | 507 | 0.9044 (0.003) | 0.9075 (0.003) | 0.9041 (0.003) | 0.9037 (0.003) |
| 0.1, 0.3 | 20 | 0.9281 (0.003) | 0.9038 (0.003) | 0.9194 (0.003) | 0.9514 (0.002) |
| 0.1, 0.3 | 30 | 0.9079 (0.003) | 0.8793 (0.003) | 0.9043 (0.003) | 0.9365 (0.002) |
| 0.1, 0.3 | 40 | 0.8990 (0.003) | 0.8666 (0.003) | 0.9038 (0.003) | 0.9232 (0.003) |
| 0.1, 0.3 | 50 | 0.8975 (0.003) | 0.8612 (0.003) | 0.9014 (0.003) | 0.9169 (0.003) |
| 0.1, 0.3 | 60 | 0.8962 (0.003) | 0.8654 (0.003) | 0.8992 (0.003) | 0.9124 (0.003) |
| 0.1, 0.3 | 70 | 0.8955 (0.003) | 0.8637 (0.003) | 0.8967 (0.003) | 0.9124 (0.003) |
| 0.1, 0.3 | 80 | 0.8975 (0.003) | 0.8678 (0.003) | 0.8951 (0.003) | 0.9067 (0.003) |
| 0.1, 0.3 | 90 | 0.8993 (0.003) | 0.8727 (0.003) | 0.8999 (0.003) | 0.9083 (0.003) |
| 0.1, 0.3 | 100 | 0.9002 (0.003) | 0.8679 (0.003) | 0.9005 (0.003) | 0.9076 (0.003) |
| 0.1, 0.3 | 200 | 0.9020 (0.003) | 0.8867 (0.003) | 0.9051 (0.003) | 0.9066 (0.003) |
| 0.1, 0.3 | 507 | 0.8940 (0.003) | 0.9341 (0.002) | 0.8959 (0.003) | 0.8997 (0.003) |

Table 15: Coverage for the scenarios with non-inferiority margin $\delta=0.05$, true risk difference $\theta=0$ and within-patient correlation $\rho=0.5$; Monte-Carlo standard error in parenthesis

| Probability of missingness per group | Cases | GEE | Hybrid CI | Nam/Tango | Nam/Tango with multiple imputation |
| --- | --- | --- | --- | --- | --- |
| 0, 0 | 20 | 0.8963 (0.003) | 0.9181 (0.003) | 0.9070 (0.003) | 0.8850 (0.003) |
| 0, 0 | 30 | 0.9035 (0.003) | 0.9132 (0.003) | 0.9052 (0.003) | 0.8902 (0.003) |
| 0, 0 | 40 | 0.9047 (0.003) | 0.9129 (0.003) | 0.9048 (0.003) | 0.9005 (0.003) |
| 0, 0 | 50 | 0.8981 (0.003) | 0.9121 (0.003) | 0.8982 (0.003) | 0.9031 (0.003) |
| 0, 0 | 60 | 0.8922 (0.003) | 0.9052 (0.003) | 0.8922 (0.003) | 0.9032 (0.003) |
| 0, 0 | 70 | 0.8944 (0.003) | 0.9054 (0.003) | 0.8944 (0.003) | 0.9044 (0.003) |
| 0, 0 | 80 | 0.8954 (0.003) | 0.9017 (0.003) | 0.8954 (0.003) | 0.9030 (0.003) |
| 0, 0 | 90 | 0.9012 (0.003) | 0.9063 (0.003) | 0.9012 (0.003) | 0.9135 (0.003) |
| 0, 0 | 100 | 0.8984 (0.003) | 0.9006 (0.003) | 0.8984 (0.003) | 0.9095 (0.003) |
| 0, 0 | 200 | 0.9055 (0.003) | 0.9055 (0.003) | 0.9055 (0.003) | 0.9159 (0.003) |
| 0, 0 | 507 | 0.8948 (0.003) | 0.8968 (0.003) | 0.8948 (0.003) | 0.9059 (0.003) |
| 0.15, 0.15 | 20 | 0.9314 (0.003) | 0.9337 (0.002) | 0.9253 (0.003) | 0.9464 (0.002) |
| 0.15, 0.15 | 30 | 0.9113 (0.003) | 0.9186 (0.003) | 0.9025 (0.003) | 0.9322 (0.003) |
| 0.15, 0.15 | 40 | 0.9002 (0.003) | 0.9095 (0.003) | 0.8977 (0.003) | 0.9255 (0.003) |
| 0.15, 0.15 | 50 | 0.8965 (0.003) | 0.9071 (0.003) | 0.9009 (0.003) | 0.9187 (0.003) |
| 0.15, 0.15 | 60 | 0.9001 (0.003) | 0.9094 (0.003) | 0.9022 (0.003) | 0.9194 (0.003) |
| 0.15, 0.15 | 70 | 0.8959 (0.003) | 0.9089 (0.003) | 0.9032 (0.003) | 0.9159 (0.003) |
| 0.15, 0.15 | 80 | 0.9028 (0.003) | 0.9103 (0.003) | 0.8968 (0.003) | 0.9163 (0.003) |
| 0.15, 0.15 | 90 | 0.8974 (0.003) | 0.9051 (0.003) | 0.8911 (0.003) | 0.9118 (0.003) |
| 0.15, 0.15 | 100 | 0.8965 (0.003) | 0.9038 (0.003) | 0.8975 (0.003) | 0.9089 (0.003) |
| 0.15, 0.15 | 200 | 0.8923 (0.003) | 0.9014 (0.003) | 0.8985 (0.003) | 0.9051 (0.003) |
| 0.15, 0.15 | 507 | 0.9012 (0.003) | 0.9070 (0.003) | 0.9015 (0.003) | 0.9067 (0.003) |
| 0.25, 0.25 | 20 | 0.9497 (0.002) | 0.9473 (0.002) | 0.9536 (0.002) | 0.9680 (0.002) |
| 0.25, 0.25 | 30 | 0.9214 (0.003) | 0.9272 (0.003) | 0.9170 (0.003) | 0.9493 (0.002) |
| 0.25, 0.25 | 40 | 0.9117 (0.003) | 0.9200 (0.003) | 0.9058 (0.003) | 0.9445 (0.002) |
| 0.25, 0.25 | 50 | 0.8989 (0.003) | 0.9135 (0.003) | 0.8971 (0.003) | 0.9323 (0.003) |
| 0.25, 0.25 | 60 | 0.8990 (0.003) | 0.9080 (0.003) | 0.9003 (0.003) | 0.9311 (0.003) |
| 0.25, 0.25 | 70 | 0.8984 (0.003) | 0.9082 (0.003) | 0.8995 (0.003) | 0.9222 (0.003) |
| 0.25, 0.25 | 80 | 0.9028 (0.003) | 0.9098 (0.003) | 0.9039 (0.003) | 0.9230 (0.003) |
| 0.25, 0.25 | 90 | 0.8991 (0.003) | 0.9060 (0.003) | 0.9008 (0.003) | 0.9169 (0.003) |
| 0.25, 0.25 | 100 | 0.9027 (0.003) | 0.9107 (0.003) | 0.8994 (0.003) | 0.9159 (0.003) |
| 0.25, 0.25 | 200 | 0.8951 (0.003) | 0.9048 (0.003) | 0.8956 (0.003) | 0.9041 (0.003) |
| 0.25, 0.25 | 507 | 0.9023 (0.003) | 0.9094 (0.003) | 0.9008 (0.003) | 0.9040 (0.003) |
| 0.1, 0.3 | 20 | 0.9403 (0.002) | 0.9116 (0.003) | 0.9366 (0.002) | 0.9584 (0.002) |
| 0.1, 0.3 | 30 | 0.9182 (0.003) | 0.8942 (0.003) | 0.9103 (0.003) | 0.9438 (0.002) |
| 0.1, 0.3 | 40 | 0.9004 (0.003) | 0.8749 (0.003) | 0.9012 (0.003) | 0.9325 (0.003) |
| 0.1, 0.3 | 50 | 0.8964 (0.003) | 0.8718 (0.003) | 0.9057 (0.003) | 0.9283 (0.003) |
| 0.1, 0.3 | 60 | 0.8975 (0.003) | 0.8696 (0.003) | 0.9008 (0.003) | 0.9220 (0.003) |
| 0.1, 0.3 | 70 | 0.8911 (0.003) | 0.8708 (0.003) | 0.8999 (0.003) | 0.9166 (0.003) |
| 0.1, 0.3 | 80 | 0.8964 (0.003) | 0.8721 (0.003) | 0.8968 (0.003) | 0.9157 (0.003) |
| 0.1, 0.3 | 90 | 0.8958 (0.003) | 0.8787 (0.003) | 0.8952 (0.003) | 0.9117 (0.003) |
| 0.1, 0.3 | 100 | 0.9022 (0.003) | 0.8844 (0.003) | 0.8965 (0.003) | 0.9128 (0.003) |
| 0.1, 0.3 | 200 | 0.9030 (0.003) | 0.9090 (0.003) | 0.9033 (0.003) | 0.9099 (0.003) |
| 0.1, 0.3 | 507 | 0.8972 (0.003) | 0.9563 (0.002) | 0.8951 (0.003) | 0.9026 (0.003) |

Table 16: Interval width for the scenarios with non-inferiority margin $\delta=0.05$, true risk difference $\theta=0$ and within-patient correlation $\rho=0$

| Probability of missingness per group | Cases | GEE | Hybrid CI | Nam/Tango | Nam/Tango with multiple imputation |
| --- | --- | --- | --- | --- | --- |
| 0, 0 | 20 | 0.4382 | 0.4299 | 0.4270 | 0.4156 |
| 0, 0 | 30 | 0.3489 | 0.3473 | 0.3451 | 0.3381 |
| 0, 0 | 40 | 0.2997 | 0.2994 | 0.2978 | 0.2934 |
| 0, 0 | 50 | 0.2677 | 0.2676 | 0.2665 | 0.2635 |
| 0, 0 | 60 | 0.2431 | 0.2433 | 0.2425 | 0.2400 |
| 0, 0 | 70 | 0.2251 | 0.2252 | 0.2246 | 0.2228 |
| 0, 0 | 80 | 0.2100 | 0.2102 | 0.2097 | 0.2083 |
| 0, 0 | 90 | 0.1977 | 0.1979 | 0.1975 | 0.1964 |
| 0, 0 | 100 | 0.1875 | 0.1876 | 0.1873 | 0.1864 |
| 0, 0 | 200 | 0.1321 | 0.1322 | 0.1321 | 0.1320 |
| 0, 0 | 507 | 0.0828 | 0.0828 | 0.0828 | 0.0830 |
| 0.15, 0.15 | 20 | 0.4738 | 0.4708 | 0.5162 | 0.4621 |
| 0.15, 0.15 | 30 | 0.3766 | 0.3804 | 0.4159 | 0.3741 |
| 0.15, 0.15 | 40 | 0.3240 | 0.3288 | 0.3590 | 0.3228 |
| 0.15, 0.15 | 50 | 0.2886 | 0.2922 | 0.3199 | 0.2887 |
| 0.15, 0.15 | 60 | 0.2635 | 0.2668 | 0.2917 | 0.2637 |
| 0.15, 0.15 | 70 | 0.2430 | 0.2461 | 0.2692 | 0.2437 |
| 0.15, 0.15 | 80 | 0.2275 | 0.2302 | 0.2519 | 0.2285 |
| 0.15, 0.15 | 90 | 0.2143 | 0.2166 | 0.2373 | 0.2154 |
| 0.15, 0.15 | 100 | 0.2030 | 0.2051 | 0.2247 | 0.2040 |
| 0.15, 0.15 | 200 | 0.1430 | 0.1440 | 0.1581 | 0.1441 |
| 0.15, 0.15 | 507 | 0.0897 | 0.0902 | 0.0990 | 0.0905 |
| 0.25, 0.25 | 20 | 0.5065 | 0.5027 | 0.6195 | 0.4999 |
| 0.25, 0.25 | 30 | 0.4008 | 0.4090 | 0.4967 | 0.4032 |
| 0.25, 0.25 | 40 | 0.3435 | 0.3523 | 0.4283 | 0.3469 |
| 0.25, 0.25 | 50 | 0.3067 | 0.3139 | 0.3815 | 0.3105 |
| 0.25, 0.25 | 60 | 0.2799 | 0.2868 | 0.3486 | 0.2836 |
| 0.25, 0.25 | 70 | 0.2584 | 0.2639 | 0.3202 | 0.2618 |
| 0.25, 0.25 | 80 | 0.2414 | 0.2461 | 0.2990 | 0.2442 |
| 0.25, 0.25 | 90 | 0.2273 | 0.2318 | 0.2815 | 0.2302 |
| 0.25, 0.25 | 100 | 0.2156 | 0.2196 | 0.2668 | 0.2186 |
| 0.25, 0.25 | 200 | 0.1523 | 0.1544 | 0.1877 | 0.1543 |
| 0.25, 0.25 | 507 | 0.0955 | 0.0964 | 0.1173 | 0.0968 |
| 0.1, 0.3 | 20 | 0.4927 | 0.4211 | 0.5616 | 0.4841 |
| 0.1, 0.3 | 30 | 0.3907 | 0.3388 | 0.4515 | 0.3905 |
| 0.1, 0.3 | 40 | 0.3359 | 0.2916 | 0.3886 | 0.3377 |
| 0.1, 0.3 | 50 | 0.2996 | 0.2606 | 0.3466 | 0.3017 |
| 0.1, 0.3 | 60 | 0.2730 | 0.2382 | 0.3157 | 0.2752 |
| 0.1, 0.3 | 70 | 0.2521 | 0.2207 | 0.2918 | 0.2544 |
| 0.1, 0.3 | 80 | 0.2357 | 0.2076 | 0.2722 | 0.2381 |
| 0.1, 0.3 | 90 | 0.2223 | 0.1968 | 0.2566 | 0.2248 |
| 0.1, 0.3 | 100 | 0.2105 | 0.1875 | 0.2430 | 0.2124 |
| 0.1, 0.3 | 200 | 0.1486 | 0.1420 | 0.1710 | 0.1501 |
| 0.1, 0.3 | 507 | 0.0932 | 0.1098 | 0.1070 | 0.0944 |

Table 17: Interval width for the scenarios with non-inferiority margin $\delta=0.05$, true risk difference $\theta=0$ and within-patient correlation $\rho=0.37$

| Probability of missingness per group | Cases | GEE | Hybrid CI | Nam/Tango | Nam/Tango with multiple imputation |
| --- | --- | --- | --- | --- | --- |
| 0, 0 | 20 | 0.3384 | 0.3461 | 0.3618 | 0.3265 |
| 0, 0 | 30 | 0.2739 | 0.2801 | 0.2885 | 0.2694 |
| 0, 0 | 40 | 0.2358 | 0.2405 | 0.2463 | 0.2339 |
| 0, 0 | 50 | 0.2104 | 0.2140 | 0.2185 | 0.2098 |
| 0, 0 | 60 | 0.1918 | 0.1946 | 0.1983 | 0.1920 |
| 0, 0 | 70 | 0.1775 | 0.1798 | 0.1827 | 0.1781 |
| 0, 0 | 80 | 0.1657 | 0.1677 | 0.1702 | 0.1666 |
| 0, 0 | 90 | 0.1562 | 0.1579 | 0.1599 | 0.1571 |
| 0, 0 | 100 | 0.1479 | 0.1494 | 0.1512 | 0.1490 |
| 0, 0 | 200 | 0.1044 | 0.1050 | 0.1057 | 0.1058 |
| 0, 0 | 507 | 0.0656 | 0.0657 | 0.0659 | 0.0666 |
| 0.15, 0.15 | 20 | 0.3925 | 0.4025 | 0.4543 | 0.3921 |
| 0.15, 0.15 | 30 | 0.3070 | 0.3207 | 0.3548 | 0.3118 |
| 0.15, 0.15 | 40 | 0.2632 | 0.2755 | 0.3006 | 0.2684 |
| 0.15, 0.15 | 50 | 0.2353 | 0.2454 | 0.2660 | 0.2400 |
| 0.15, 0.15 | 60 | 0.2145 | 0.2235 | 0.2407 | 0.2189 |
| 0.15, 0.15 | 70 | 0.1988 | 0.2064 | 0.2218 | 0.2029 |
| 0.15, 0.15 | 80 | 0.1858 | 0.1927 | 0.2063 | 0.1893 |
| 0.15, 0.15 | 90 | 0.1747 | 0.1812 | 0.1934 | 0.1786 |
| 0.15, 0.15 | 100 | 0.1659 | 0.1718 | 0.1830 | 0.1694 |
| 0.15, 0.15 | 200 | 0.1172 | 0.1205 | 0.1273 | 0.1196 |
| 0.15, 0.15 | 507 | 0.0735 | 0.0754 | 0.0788 | 0.0751 |
| 0.25, 0.25 | 20 | 0.4451 | 0.4478 | 0.5610 | 0.4461 |
| 0.25, 0.25 | 30 | 0.3430 | 0.3582 | 0.4363 | 0.3514 |
| 0.25, 0.25 | 40 | 0.2906 | 0.3072 | 0.3666 | 0.3001 |
| 0.25, 0.25 | 50 | 0.2590 | 0.2729 | 0.3231 | 0.2673 |
| 0.25, 0.25 | 60 | 0.2358 | 0.2479 | 0.2911 | 0.2431 |
| 0.25, 0.25 | 70 | 0.2176 | 0.2287 | 0.2667 | 0.2241 |
| 0.25, 0.25 | 80 | 0.2041 | 0.2146 | 0.2486 | 0.2098 |
| 0.25, 0.25 | 90 | 0.1921 | 0.2016 | 0.2328 | 0.1974 |
| 0.25, 0.25 | 100 | 0.1826 | 0.1913 | 0.2202 | 0.1877 |
| 0.25, 0.25 | 200 | 0.1288 | 0.1343 | 0.1518 | 0.1320 |
| 0.25, 0.25 | 507 | 0.0808 | 0.0839 | 0.0937 | 0.0828 |
| 0.1, 0.3 | 20 | 0.4162 | 0.3625 | 0.4989 | 0.4196 |
| 0.1, 0.3 | 30 | 0.3264 | 0.2893 | 0.3913 | 0.3348 |
| 0.1, 0.3 | 40 | 0.2777 | 0.2477 | 0.3297 | 0.2858 |
| 0.1, 0.3 | 50 | 0.2478 | 0.2221 | 0.2907 | 0.2553 |
| 0.1, 0.3 | 60 | 0.2255 | 0.2032 | 0.2622 | 0.2317 |
| 0.1, 0.3 | 70 | 0.2088 | 0.1897 | 0.2412 | 0.2143 |
| 0.1, 0.3 | 80 | 0.1954 | 0.1793 | 0.2243 | 0.2006 |
| 0.1, 0.3 | 90 | 0.1846 | 0.1709 | 0.2112 | 0.1893 |
| 0.1, 0.3 | 100 | 0.1745 | 0.1632 | 0.1988 | 0.1790 |
| 0.1, 0.3 | 200 | 0.1233 | 0.1281 | 0.1378 | 0.1265 |
| 0.1, 0.3 | 507 | 0.0774 | 0.1045 | 0.0853 | 0.0792 |

Table 18: Interval width for the scenarios with non-inferiority margin $\delta=0.05$, true risk difference $\theta=0$ and within-patient correlation $\rho=0.5$

| Probability of missingness per group | Cases | GEE | Hybrid CI | Nam/Tango | Nam/Tango with multiple imputation |
| --- | --- | --- | --- | --- | --- |
| 0, 0 | 20 | 0.3000 | 0.3155 | 0.3394 | 0.2941 |
| 0, 0 | 30 | 0.2431 | 0.2533 | 0.2668 | 0.2422 |
| 0, 0 | 40 | 0.2097 | 0.2170 | 0.2262 | 0.2110 |
| 0, 0 | 50 | 0.1878 | 0.1932 | 0.2001 | 0.1899 |
| 0, 0 | 60 | 0.1712 | 0.1754 | 0.1809 | 0.1736 |
| 0, 0 | 70 | 0.1584 | 0.1618 | 0.1664 | 0.1610 |
| 0, 0 | 80 | 0.1477 | 0.1506 | 0.1544 | 0.1503 |
| 0, 0 | 90 | 0.1395 | 0.1419 | 0.1451 | 0.1422 |
| 0, 0 | 100 | 0.1323 | 0.1343 | 0.1372 | 0.1350 |
| 0, 0 | 200 | 0.0933 | 0.0941 | 0.0952 | 0.0957 |
| 0, 0 | 507 | 0.0586 | 0.0588 | 0.0591 | 0.0602 |
| 0.15, 0.15 | 20 | 0.3611 | 0.3781 | 0.4334 | 0.3676 |
| 0.15, 0.15 | 30 | 0.2794 | 0.2988 | 0.3342 | 0.2902 |
| 0.15, 0.15 | 40 | 0.2388 | 0.2555 | 0.2798 | 0.2483 |
| 0.15, 0.15 | 50 | 0.2119 | 0.2261 | 0.2452 | 0.2207 |
| 0.15, 0.15 | 60 | 0.1937 | 0.2056 | 0.2214 | 0.2012 |
| 0.15, 0.15 | 70 | 0.1795 | 0.1902 | 0.2032 | 0.1864 |
| 0.15, 0.15 | 80 | 0.1673 | 0.1771 | 0.1881 | 0.1733 |
| 0.15, 0.15 | 90 | 0.1577 | 0.1666 | 0.1762 | 0.1635 |
| 0.15, 0.15 | 100 | 0.1497 | 0.1577 | 0.1664 | 0.1551 |
| 0.15, 0.15 | 200 | 0.1058 | 0.1107 | 0.1147 | 0.1095 |
| 0.15, 0.15 | 507 | 0.0665 | 0.0692 | 0.0708 | 0.0687 |
| 0.25, 0.25 | 20 | 0.4203 | 0.4258 | 0.5410 | 0.4275 |
| 0.25, 0.25 | 30 | 0.3206 | 0.3403 | 0.4160 | 0.3344 |
| 0.25, 0.25 | 40 | 0.2687 | 0.2876 | 0.3459 | 0.2832 |
| 0.25, 0.25 | 50 | 0.2374 | 0.2559 | 0.3009 | 0.2503 |
| 0.25, 0.25 | 60 | 0.2162 | 0.2319 | 0.2701 | 0.2276 |
| 0.25, 0.25 | 70 | 0.1986 | 0.2141 | 0.2455 | 0.2088 |
| 0.25, 0.25 | 80 | 0.1861 | 0.2002 | 0.2274 | 0.1946 |
| 0.25, 0.25 | 90 | 0.1759 | 0.1887 | 0.2137 | 0.1835 |
| 0.25, 0.25 | 100 | 0.1668 | 0.1786 | 0.2013 | 0.1738 |
| 0.25, 0.25 | 200 | 0.1179 | 0.1257 | 0.1375 | 0.1222 |
| 0.25, 0.25 | 507 | 0.0741 | 0.0786 | 0.0843 | 0.0765 |
| 0.1, 0.3 | 20 | 0.3878 | 0.3423 | 0.4787 | 0.3975 |
| 0.1, 0.3 | 30 | 0.2998 | 0.2700 | 0.3702 | 0.3131 |
| 0.1, 0.3 | 40 | 0.2528 | 0.2300 | 0.3078 | 0.2658 |
| 0.1, 0.3 | 50 | 0.2248 | 0.2058 | 0.2693 | 0.2360 |
| 0.1, 0.3 | 60 | 0.2046 | 0.1888 | 0.2418 | 0.2145 |
| 0.1, 0.3 | 70 | 0.1893 | 0.1765 | 0.2215 | 0.1981 |
| 0.1, 0.3 | 80 | 0.1769 | 0.1669 | 0.2053 | 0.1845 |
| 0.1, 0.3 | 90 | 0.1668 | 0.1594 | 0.1921 | 0.1739 |
| 0.1, 0.3 | 100 | 0.1582 | 0.1532 | 0.1812 | 0.1646 |
| 0.1, 0.3 | 200 | 0.1119 | 0.1223 | 0.1245 | 0.1161 |
| 0.1, 0.3 | 507 | 0.0704 | 0.1021 | 0.0767 | 0.0729 |

Table 19: Power for the scenarios with non-inferiority margin $\delta=0.1$, true risk difference $\theta=0$ and within-patient correlation $\rho=0$; Monte-Carlo standard error in parenthesis

| Probability of missingness per group | Cases | GEE | Hybrid CI | Nam/Tango | Nam/Tango with multiple imputation |
| --- | --- | --- | --- | --- | --- |
| 0, 0 | 20 | 0.1667 (0.004) | 0.1569 (0.004) | 0.1667 (0.004) | 0.1667 (0.004) |
| 0, 0 | 30 | 0.2508 (0.004) | 0.2266 (0.004) | 0.2408 (0.004) | 0.2408 (0.004) |
| 0, 0 | 40 | 0.2964 (0.005) | 0.2980 (0.005) | 0.3037 (0.005) | 0.3037 (0.005) |
| 0, 0 | 50 | 0.3331 (0.005) | 0.3317 (0.005) | 0.3326 (0.005) | 0.3326 (0.005) |
| 0, 0 | 60 | 0.3868 (0.005) | 0.3762 (0.005) | 0.3839 (0.005) | 0.3839 (0.005) |
| 0, 0 | 70 | 0.4195 (0.005) | 0.4204 (0.005) | 0.4194 (0.005) | 0.4194 (0.005) |
| 0, 0 | 80 | 0.4693 (0.005) | 0.4754 (0.005) | 0.4789 (0.005) | 0.4789 (0.005) |
| 0, 0 | 90 | 0.5005 (0.005) | 0.5007 (0.005) | 0.5005 (0.005) | 0.5005 (0.005) |
| 0, 0 | 100 | 0.5442 (0.005) | 0.5402 (0.005) | 0.5407 (0.005) | 0.5407 (0.005) |
| 0, 0 | 200 | 0.8006 (0.004) | 0.8021 (0.004) | 0.8020 (0.004) | 0.8020 (0.004) |
| 0, 0 | 507 | 0.9897 (0.001) | 0.9900 (0.001) | 0.9900 (0.001) | 0.9900 (0.001) |
| 0.15, 0.15 | 20 | 0.1663 (0.004) | 0.1561 (0.004) | 0.1314 (0.003) | 0.1428 (0.003) |
| 0.15, 0.15 | 30 | 0.2325 (0.004) | 0.2219 (0.004) | 0.1982 (0.004) | 0.2166 (0.004) |
| 0.15, 0.15 | 40 | 0.2587 (0.004) | 0.2472 (0.004) | 0.2231 (0.004) | 0.2492 (0.004) |
| 0.15, 0.15 | 50 | 0.3110 (0.005) | 0.2978 (0.005) | 0.2692 (0.004) | 0.2991 (0.005) |
| 0.15, 0.15 | 60 | 0.3473 (0.005) | 0.3391 (0.005) | 0.3008 (0.005) | 0.3392 (0.005) |
| 0.15, 0.15 | 70 | 0.3878 (0.005) | 0.3821 (0.005) | 0.3357 (0.005) | 0.3766 (0.005) |
| 0.15, 0.15 | 80 | 0.4260 (0.005) | 0.4190 (0.005) | 0.3749 (0.005) | 0.4187 (0.005) |
| 0.15, 0.15 | 90 | 0.4550 (0.005) | 0.4465 (0.005) | 0.3947 (0.005) | 0.4454 (0.005) |
| 0.15, 0.15 | 100 | 0.4910 (0.005) | 0.4828 (0.005) | 0.4263 (0.005) | 0.4824 (0.005) |
| 0.15, 0.15 | 200 | 0.7407 (0.004) | 0.7345 (0.004) | 0.6640 (0.005) | 0.7344 (0.004) |
| 0.15, 0.15 | 507 | 0.9795 (0.001) | 0.9760 (0.002) | 0.9539 (0.002) | 0.9782 (0.001) |
| 0.25, 0.25 | 20 | 0.1535 (0.004) | 0.1452 (0.004) | 0.1124 (0.003) | 0.1171 (0.003) |
| 0.25, 0.25 | 30 | 0.2096 (0.004) | 0.1996 (0.004) | 0.1515 (0.004) | 0.1814 (0.004) |
| 0.25, 0.25 | 40 | 0.2440 (0.004) | 0.2316 (0.004) | 0.1790 (0.004) | 0.2248 (0.004) |
| 0.25, 0.25 | 50 | 0.2917 (0.005) | 0.2856 (0.005) | 0.2153 (0.004) | 0.2730 (0.004) |
| 0.25, 0.25 | 60 | 0.3179 (0.005) | 0.3121 (0.005) | 0.2392 (0.004) | 0.2992 (0.005) |
| 0.25, 0.25 | 70 | 0.3490 (0.005) | 0.3449 (0.005) | 0.2516 (0.004) | 0.3394 (0.005) |
| 0.25, 0.25 | 80 | 0.3927 (0.005) | 0.3818 (0.005) | 0.2905 (0.005) | 0.3769 (0.005) |
| 0.25, 0.25 | 90 | 0.4264 (0.005) | 0.4184 (0.005) | 0.3228 (0.005) | 0.4105 (0.005) |
| 0.25, 0.25 | 100 | 0.4471 (0.005) | 0.4363 (0.005) | 0.3366 (0.005) | 0.4350 (0.005) |
| 0.25, 0.25 | 200 | 0.7074 (0.005) | 0.6925 (0.005) | 0.5549 (0.005) | 0.6970 (0.005) |
| 0.25, 0.25 | 507 | 0.9620 (0.002) | 0.9566 (0.002) | 0.8720 (0.003) | 0.9597 (0.002) |
| 0.1, 0.3 | 20 | 0.1483 (0.004) | 0.2377 (0.004) | 0.1048 (0.003) | 0.0992 (0.003) |
| 0.1, 0.3 | 30 | 0.2206 (0.004) | 0.3485 (0.005) | 0.1682 (0.004) | 0.1649 (0.004) |
| 0.1, 0.3 | 40 | 0.2715 (0.004) | 0.4288 (0.005) | 0.2109 (0.004) | 0.2233 (0.004) |
| 0.1, 0.3 | 50 | 0.3020 (0.005) | 0.4924 (0.005) | 0.2431 (0.004) | 0.2581 (0.004) |
| 0.1, 0.3 | 60 | 0.3442 (0.005) | 0.5479 (0.005) | 0.2741 (0.004) | 0.3058 (0.005) |
| 0.1, 0.3 | 70 | 0.3713 (0.005) | 0.5880 (0.005) | 0.3008 (0.005) | 0.3318 (0.005) |
| 0.1, 0.3 | 80 | 0.4005 (0.005) | 0.6372 (0.005) | 0.3267 (0.005) | 0.3701 (0.005) |
| 0.1, 0.3 | 90 | 0.4379 (0.005) | 0.6815 (0.005) | 0.3606 (0.005) | 0.4043 (0.005) |
| 0.1, 0.3 | 100 | 0.4764 (0.005) | 0.7177 (0.005) | 0.3944 (0.005) | 0.4421 (0.005) |
| 0.1, 0.3 | 200 | 0.7129 (0.005) | 0.8894 (0.003) | 0.6102 (0.005) | 0.6892 (0.005) |
| 0.1, 0.3 | 507 | 0.9696 (0.002) | 0.9856 (0.001) | 0.9234 (0.003) | 0.9632 (0.002) |

Table 20: Power for the scenarios with non-inferiority margin $\delta=0.1$, true risk difference $\theta=0$ and within-patient correlation $\rho=0.37$; Monte-Carlo standard error in parenthesis

| Probability of missingness per group | Cases | GEE | Hybrid CI | Nam/Tango | Nam/Tango with multiple imputation |
| --- | --- | --- | --- | --- | --- |
| 0, 0 | 20 | 0.2184 (0.004) | 0.2268 (0.004) | 0.2094 (0.004) | 0.2094 (0.004) |
| 0, 0 | 30 | 0.3609 (0.005) | 0.3228 (0.005) | 0.2857 (0.005) | 0.2857 (0.005) |
| 0, 0 | 40 | 0.3922 (0.005) | 0.3853 (0.005) | 0.3844 (0.005) | 0.3844 (0.005) |
| 0, 0 | 50 | 0.4769 (0.005) | 0.4606 (0.005) | 0.4360 (0.005) | 0.4360 (0.005) |
| 0, 0 | 60 | 0.5208 (0.005) | 0.5126 (0.005) | 0.4928 (0.005) | 0.4928 (0.005) |
| 0, 0 | 70 | 0.5656 (0.005) | 0.5633 (0.005) | 0.5596 (0.005) | 0.5596 (0.005) |
| 0, 0 | 80 | 0.6420 (0.005) | 0.6274 (0.005) | 0.6072 (0.005) | 0.6072 (0.005) |
| 0, 0 | 90 | 0.6710 (0.005) | 0.6742 (0.005) | 0.6678 (0.005) | 0.6678 (0.005) |
| 0, 0 | 100 | 0.7236 (0.004) | 0.7166 (0.005) | 0.7089 (0.005) | 0.7089 (0.005) |
| 0, 0 | 200 | 0.9358 (0.002) | 0.9351 (0.002) | 0.9311 (0.003) | 0.9311 (0.003) |
| 0, 0 | 507 | 0.9995 (0.000) | 0.9994 (0.000) | 0.9994 (0.000) | 0.9994 (0.000) |
| 0.15, 0.15 | 20 | 0.2295 (0.004) | 0.2154 (0.004) | 0.1657 (0.004) | 0.1675 (0.004) |
| 0.15, 0.15 | 30 | 0.2925 (0.005) | 0.2753 (0.004) | 0.2301 (0.004) | 0.2427 (0.004) |
| 0.15, 0.15 | 40 | 0.3526 (0.005) | 0.3257 (0.005) | 0.2871 (0.005) | 0.3054 (0.005) |
| 0.15, 0.15 | 50 | 0.4117 (0.005) | 0.3882 (0.005) | 0.3454 (0.005) | 0.3731 (0.005) |
| 0.15, 0.15 | 60 | 0.4756 (0.005) | 0.4527 (0.005) | 0.4079 (0.005) | 0.4391 (0.005) |
| 0.15, 0.15 | 70 | 0.5038 (0.005) | 0.4770 (0.005) | 0.4284 (0.005) | 0.4663 (0.005) |
| 0.15, 0.15 | 80 | 0.5505 (0.005) | 0.5278 (0.005) | 0.4786 (0.005) | 0.5194 (0.005) |
| 0.15, 0.15 | 90 | 0.5947 (0.005) | 0.5664 (0.005) | 0.5269 (0.005) | 0.5647 (0.005) |
| 0.15, 0.15 | 100 | 0.6346 (0.005) | 0.6114 (0.005) | 0.5594 (0.005) | 0.6072 (0.005) |
| 0.15, 0.15 | 200 | 0.8768 (0.003) | 0.8582 (0.003) | 0.8280 (0.004) | 0.8618 (0.003) |
| 0.15, 0.15 | 507 | 0.9978 (0.000) | 0.9966 (0.001) | 0.9941 (0.001) | 0.9973 (0.001) |
| 0.25, 0.25 | 20 | 0.1972 (0.004) | 0.1899 (0.004) | 0.1142 (0.003) | 0.1241 (0.003) |
| 0.25, 0.25 | 30 | 0.2609 (0.004) | 0.2509 (0.004) | 0.1792 (0.004) | 0.2013 (0.004) |
| 0.25, 0.25 | 40 | 0.3102 (0.005) | 0.2976 (0.005) | 0.2245 (0.004) | 0.2636 (0.004) |
| 0.25, 0.25 | 50 | 0.3658 (0.005) | 0.3424 (0.005) | 0.2630 (0.004) | 0.3183 (0.005) |
| 0.25, 0.25 | 60 | 0.4135 (0.005) | 0.3826 (0.005) | 0.2983 (0.005) | 0.3691 (0.005) |
| 0.25, 0.25 | 70 | 0.4517 (0.005) | 0.4209 (0.005) | 0.3427 (0.005) | 0.4142 (0.005) |
| 0.25, 0.25 | 80 | 0.4941 (0.005) | 0.4641 (0.005) | 0.3724 (0.005) | 0.4610 (0.005) |
| 0.25, 0.25 | 90 | 0.5371 (0.005) | 0.5020 (0.005) | 0.4130 (0.005) | 0.5004 (0.005) |
| 0.25, 0.25 | 100 | 0.5638 (0.005) | 0.5389 (0.005) | 0.4460 (0.005) | 0.5308 (0.005) |
| 0.25, 0.25 | 200 | 0.8145 (0.004) | 0.7843 (0.004) | 0.6926 (0.005) | 0.8002 (0.004) |
| 0.25, 0.25 | 507 | 0.9919 (0.001) | 0.9882 (0.001) | 0.9702 (0.002) | 0.9895 (0.001) |
| 0.1, 0.3 | 20 | 0.2294 (0.004) | 0.3089 (0.005) | 0.1272 (0.003) | 0.1203 (0.003) |
| 0.1, 0.3 | 30 | 0.2841 (0.005) | 0.4051 (0.005) | 0.2114 (0.004) | 0.1964 (0.004) |
| 0.1, 0.3 | 40 | 0.3497 (0.005) | 0.4945 (0.005) | 0.2520 (0.004) | 0.2553 (0.004) |
| 0.1, 0.3 | 50 | 0.3979 (0.005) | 0.5541 (0.005) | 0.2983 (0.005) | 0.3184 (0.005) |
| 0.1, 0.3 | 60 | 0.4455 (0.005) | 0.6171 (0.005) | 0.3482 (0.005) | 0.3700 (0.005) |
| 0.1, 0.3 | 70 | 0.4895 (0.005) | 0.6732 (0.005) | 0.3950 (0.005) | 0.4241 (0.005) |
| 0.1, 0.3 | 80 | 0.5316 (0.005) | 0.7191 (0.004) | 0.4376 (0.005) | 0.4718 (0.005) |
| 0.1, 0.3 | 90 | 0.5658 (0.005) | 0.7462 (0.004) | 0.4695 (0.005) | 0.5035 (0.005) |
| 0.1, 0.3 | 100 | 0.6012 (0.005) | 0.7808 (0.004) | 0.5023 (0.005) | 0.5459 (0.005) |
| 0.1, 0.3 | 200 | 0.8396 (0.004) | 0.9139 (0.003) | 0.7683 (0.004) | 0.8071 (0.004) |
| 0.1, 0.3 | 507 | 0.9942 (0.001) | 0.9903 (0.001) | 0.9851 (0.001) | 0.9908 (0.001) |

Table 21: Power for the scenarios with non-inferiority margin $\delta=0.1$, true risk difference $\theta=0$ and within-patient correlation $\rho=0.5$; Monte-Carlo standard error in parenthesis

| Probability of missingness per group | Cases | GEE | Hybrid CI | Nam/Tango | Nam/Tango with multiple imputation |
| --- | --- | --- | --- | --- | --- |
| 0, 0 | 20 | 0.2575 (0.004) | 0.2757 (0.004) | 0.2322 (0.004) | 0.2322 (0.004) |
| 0, 0 | 30 | 0.4382 (0.005) | 0.3846 (0.005) | 0.3183 (0.005) | 0.3183 (0.005) |
| 0, 0 | 40 | 0.4719 (0.005) | 0.4494 (0.005) | 0.4453 (0.005) | 0.4453 (0.005) |
| 0, 0 | 50 | 0.5611 (0.005) | 0.5319 (0.005) | 0.4901 (0.005) | 0.4901 (0.005) |
| 0, 0 | 60 | 0.6001 (0.005) | 0.5960 (0.005) | 0.5737 (0.005) | 0.5737 (0.005) |
| 0, 0 | 70 | 0.6670 (0.005) | 0.6571 (0.005) | 0.6454 (0.005) | 0.6454 (0.005) |
| 0, 0 | 80 | 0.7260 (0.004) | 0.7094 (0.005) | 0.6795 (0.005) | 0.6795 (0.005) |
| 0, 0 | 90 | 0.7546 (0.004) | 0.7548 (0.004) | 0.7375 (0.004) | 0.7375 (0.004) |
| 0, 0 | 100 | 0.8015 (0.004) | 0.7916 (0.004) | 0.7761 (0.004) | 0.7761 (0.004) |
| 0, 0 | 200 | 0.9694 (0.002) | 0.9679 (0.002) | 0.9649 (0.002) | 0.9649 (0.002) |
| 0, 0 | 507 | 1.0000 (0.000) | 1.0000 (0.000) | 1.0000 (0.000) | 1.0000 (0.000) |
| 0.15, 0.15 | 20 | 0.2577 (0.004) | 0.2406 (0.004) | 0.1726 (0.004) | 0.1737 (0.004) |
| 0.15, 0.15 | 30 | 0.3359 (0.005) | 0.3083 (0.005) | 0.2538 (0.004) | 0.2644 (0.004) |
| 0.15, 0.15 | 40 | 0.4180 (0.005) | 0.3702 (0.005) | 0.3113 (0.005) | 0.3356 (0.005) |
| 0.15, 0.15 | 50 | 0.4821 (0.005) | 0.4418 (0.005) | 0.3857 (0.005) | 0.4168 (0.005) |
| 0.15, 0.15 | 60 | 0.5331 (0.005) | 0.4922 (0.005) | 0.4468 (0.005) | 0.4714 (0.005) |
| 0.15, 0.15 | 70 | 0.5881 (0.005) | 0.5399 (0.005) | 0.4975 (0.005) | 0.5352 (0.005) |
| 0.15, 0.15 | 80 | 0.6293 (0.005) | 0.5916 (0.005) | 0.5440 (0.005) | 0.5808 (0.005) |
| 0.15, 0.15 | 90 | 0.6768 (0.005) | 0.6290 (0.005) | 0.5898 (0.005) | 0.6326 (0.005) |
| 0.15, 0.15 | 100 | 0.7136 (0.005) | 0.6710 (0.005) | 0.6386 (0.005) | 0.6750 (0.005) |
| 0.15, 0.15 | 200 | 0.9254 (0.003) | 0.9013 (0.003) | 0.8892 (0.003) | 0.9125 (0.003) |
| 0.15, 0.15 | 507 | 0.9997 (0.000) | 0.9990 (0.000) | 0.9987 (0.000) | 0.9992 (0.000) |
| 0.25, 0.25 | 20 | 0.2312 (0.004) | 0.2194 (0.004) | 0.1148 (0.003) | 0.1300 (0.003) |
| 0.25, 0.25 | 30 | 0.2896 (0.005) | 0.2715 (0.004) | 0.2025 (0.004) | 0.2145 (0.004) |
| 0.25, 0.25 | 40 | 0.3469 (0.005) | 0.3257 (0.005) | 0.2496 (0.004) | 0.2809 (0.004) |
| 0.25, 0.25 | 50 | 0.4042 (0.005) | 0.3733 (0.005) | 0.2767 (0.004) | 0.3393 (0.005) |
| 0.25, 0.25 | 60 | 0.4668 (0.005) | 0.4268 (0.005) | 0.3375 (0.005) | 0.4062 (0.005) |
| 0.25, 0.25 | 70 | 0.5164 (0.005) | 0.4740 (0.005) | 0.3898 (0.005) | 0.4586 (0.005) |
| 0.25, 0.25 | 80 | 0.5529 (0.005) | 0.5028 (0.005) | 0.4218 (0.005) | 0.5014 (0.005) |
| 0.25, 0.25 | 90 | 0.5986 (0.005) | 0.5498 (0.005) | 0.4627 (0.005) | 0.5477 (0.005) |
| 0.25, 0.25 | 100 | 0.6367 (0.005) | 0.5830 (0.005) | 0.5047 (0.005) | 0.5880 (0.005) |
| 0.25, 0.25 | 200 | 0.8741 (0.003) | 0.8306 (0.004) | 0.7737 (0.004) | 0.8489 (0.004) |
| 0.25, 0.25 | 507 | 0.9968 (0.001) | 0.9926 (0.001) | 0.9862 (0.001) | 0.9943 (0.001) |
| 0.1, 0.3 | 20 | 0.2501 (0.004) | 0.3269 (0.005) | 0.1292 (0.003) | 0.1228 (0.003) |
| 0.1, 0.3 | 30 | 0.3173 (0.005) | 0.4460 (0.005) | 0.2328 (0.004) | 0.2074 (0.004) |
| 0.1, 0.3 | 40 | 0.3985 (0.005) | 0.5357 (0.005) | 0.2766 (0.004) | 0.2815 (0.004) |
| 0.1, 0.3 | 50 | 0.4589 (0.005) | 0.6007 (0.005) | 0.3342 (0.005) | 0.3505 (0.005) |
| 0.1, 0.3 | 60 | 0.5105 (0.005) | 0.6632 (0.005) | 0.3896 (0.005) | 0.4047 (0.005) |
| 0.1, 0.3 | 70 | 0.5546 (0.005) | 0.7069 (0.005) | 0.4385 (0.005) | 0.4595 (0.005) |
| 0.1, 0.3 | 80 | 0.5947 (0.005) | 0.7477 (0.004) | 0.4766 (0.005) | 0.5114 (0.005) |
| 0.1, 0.3 | 90 | 0.6331 (0.005) | 0.7807 (0.004) | 0.5324 (0.005) | 0.5605 (0.005) |
| 0.1, 0.3 | 100 | 0.6643 (0.005) | 0.8019 (0.004) | 0.5675 (0.005) | 0.5931 (0.005) |
| 0.1, 0.3 | 200 | 0.8984 (0.003) | 0.9321 (0.003) | 0.8369 (0.004) | 0.8622 (0.003) |
| 0.1, 0.3 | 507 | 0.9983 (0.000) | 0.9921 (0.001) | 0.9951 (0.001) | 0.9966 (0.001) |

Table 22: Coverage for the scenarios with non-inferiority margin $\delta=0.1$, true risk difference $\theta=0$ and within-patient correlation $\rho=0$; Monte-Carlo standard error in parenthesis

| Probability of missingness per group | Cases | GEE | Hybrid CI | Nam/Tango | Nam/Tango with multiple imputation |
| --- | --- | --- | --- | --- | --- |
| 0, 0 | 20 | 0.9179 (0.003) | 0.9271 (0.003) | 0.9179 (0.003) | 0.9107 (0.003) |
| 0, 0 | 30 | 0.8973 (0.003) | 0.9170 (0.003) | 0.8973 (0.003) | 0.9038 (0.003) |
| 0, 0 | 40 | 0.9004 (0.003) | 0.9114 (0.003) | 0.9004 (0.003) | 0.9046 (0.003) |
| 0, 0 | 50 | 0.8993 (0.003) | 0.9028 (0.003) | 0.8993 (0.003) | 0.9024 (0.003) |
| 0, 0 | 60 | 0.9005 (0.003) | 0.9036 (0.003) | 0.9005 (0.003) | 0.9039 (0.003) |
| 0, 0 | 70 | 0.9074 (0.003) | 0.9078 (0.003) | 0.9074 (0.003) | 0.9107 (0.003) |
| 0, 0 | 80 | 0.9025 (0.003) | 0.9040 (0.003) | 0.9025 (0.003) | 0.9055 (0.003) |
| 0, 0 | 90 | 0.8993 (0.003) | 0.9012 (0.003) | 0.8993 (0.003) | 0.9029 (0.003) |
| 0, 0 | 100 | 0.8980 (0.003) | 0.9013 (0.003) | 0.8980 (0.003) | 0.9046 (0.003) |
| 0, 0 | 200 | 0.9012 (0.003) | 0.9029 (0.003) | 0.9012 (0.003) | 0.9059 (0.003) |
| 0, 0 | 507 | 0.8969 (0.003) | 0.8969 (0.003) | 0.8969 (0.003) | 0.9014 (0.003) |
| 0.15, 0.15 | 20 | 0.9217 (0.003) | 0.9353 (0.002) | 0.9217 (0.003) | 0.9369 (0.002) |
| 0.15, 0.15 | 30 | 0.8960 (0.003) | 0.9108 (0.003) | 0.9022 (0.003) | 0.9117 (0.003) |
| 0.15, 0.15 | 40 | 0.9023 (0.003) | 0.9120 (0.003) | 0.9029 (0.003) | 0.9130 (0.003) |
| 0.15, 0.15 | 50 | 0.9027 (0.003) | 0.9114 (0.003) | 0.8945 (0.003) | 0.9111 (0.003) |
| 0.15, 0.15 | 60 | 0.9065 (0.003) | 0.9125 (0.003) | 0.9018 (0.003) | 0.9134 (0.003) |
| 0.15, 0.15 | 70 | 0.9015 (0.003) | 0.9059 (0.003) | 0.9054 (0.003) | 0.9102 (0.003) |
| 0.15, 0.15 | 80 | 0.8983 (0.003) | 0.9033 (0.003) | 0.8982 (0.003) | 0.9046 (0.003) |
| 0.15, 0.15 | 90 | 0.8985 (0.003) | 0.9045 (0.003) | 0.9029 (0.003) | 0.9082 (0.003) |
| 0.15, 0.15 | 100 | 0.9011 (0.003) | 0.9028 (0.003) | 0.9080 (0.003) | 0.9072 (0.003) |
| 0.15, 0.15 | 200 | 0.9026 (0.003) | 0.9013 (0.003) | 0.9015 (0.003) | 0.9055 (0.003) |
| 0.15, 0.15 | 507 | 0.8971 (0.003) | 0.8984 (0.003) | 0.9021 (0.003) | 0.9016 (0.003) |
| 0.25, 0.25 | 20 | 0.9273 (0.003) | 0.9432 (0.002) | 0.9221 (0.003) | 0.9503 (0.002) |
| 0.25, 0.25 | 30 | 0.9007 (0.003) | 0.9136 (0.003) | 0.9028 (0.003) | 0.9253 (0.003) |
| 0.25, 0.25 | 40 | 0.8956 (0.003) | 0.9093 (0.003) | 0.9028 (0.003) | 0.9131 (0.003) |
| 0.25, 0.25 | 50 | 0.8918 (0.003) | 0.8990 (0.003) | 0.9011 (0.003) | 0.9118 (0.003) |
| 0.25, 0.25 | 60 | 0.8920 (0.003) | 0.8979 (0.003) | 0.8871 (0.003) | 0.9048 (0.003) |
| 0.25, 0.25 | 70 | 0.8965 (0.003) | 0.9091 (0.003) | 0.9001 (0.003) | 0.9072 (0.003) |
| 0.25, 0.25 | 80 | 0.9022 (0.003) | 0.9067 (0.003) | 0.9009 (0.003) | 0.9139 (0.003) |
| 0.25, 0.25 | 90 | 0.8965 (0.003) | 0.9025 (0.003) | 0.8975 (0.003) | 0.9060 (0.003) |
| 0.25, 0.25 | 100 | 0.8977 (0.003) | 0.9021 (0.003) | 0.8997 (0.003) | 0.9052 (0.003) |
| 0.25, 0.25 | 200 | 0.9066 (0.003) | 0.9055 (0.003) | 0.8988 (0.003) | 0.9130 (0.003) |
| 0.25, 0.25 | 507 | 0.8963 (0.003) | 0.8960 (0.003) | 0.8976 (0.003) | 0.9006 (0.003) |
| 0.1, 0.3 | 20 | 0.9201 (0.003) | 0.9148 (0.003) | 0.9145 (0.003) | 0.9402 (0.002) |
| 0.1, 0.3 | 30 | 0.9075 (0.003) | 0.8906 (0.003) | 0.9152 (0.003) | 0.9247 (0.003) |
| 0.1, 0.3 | 40 | 0.8901 (0.003) | 0.8678 (0.003) | 0.8993 (0.003) | 0.9084 (0.003) |
| 0.1, 0.3 | 50 | 0.8949 (0.003) | 0.8669 (0.003) | 0.8984 (0.003) | 0.9086 (0.003) |
| 0.1, 0.3 | 60 | 0.9007 (0.003) | 0.8653 (0.003) | 0.8986 (0.003) | 0.9114 (0.003) |
| 0.1, 0.3 | 70 | 0.8932 (0.003) | 0.8583 (0.003) | 0.8948 (0.003) | 0.9032 (0.003) |
| 0.1, 0.3 | 80 | 0.9020 (0.003) | 0.8608 (0.003) | 0.9039 (0.003) | 0.9117 (0.003) |
| 0.1, 0.3 | 90 | 0.8993 (0.003) | 0.8577 (0.003) | 0.9023 (0.003) | 0.9070 (0.003) |
| 0.1, 0.3 | 100 | 0.8984 (0.003) | 0.8533 (0.004) | 0.9031 (0.003) | 0.9091 (0.003) |
| 0.1, 0.3 | 200 | 0.8960 (0.003) | 0.8489 (0.004) | 0.9005 (0.003) | 0.9000 (0.003) |
| 0.1, 0.3 | 507 | 0.9010 (0.003) | 0.8786 (0.003) | 0.8991 (0.003) | 0.9040 (0.003) |

Table 23: Coverage for the scenarios with non-inferiority margin $\delta=0.1$, true risk difference $\theta=0$ and within-patient correlation $\rho=0.37$; Monte-Carlo standard error in parenthesis

| Probability of missingness per group | Cases | GEE | Hybrid CI | Nam/Tango | Nam/Tango with multiple imputation |
| --- | --- | --- | --- | --- | --- |
| 0, 0 | 20 | 0.9063 (0.003) | 0.9174 (0.003) | 0.9100 (0.003) | 0.9207 (0.003) |
| 0, 0 | 30 | 0.9020 (0.003) | 0.9078 (0.003) | 0.9024 (0.003) | 0.9096 (0.003) |
| 0, 0 | 40 | 0.8922 (0.003) | 0.9035 (0.003) | 0.8922 (0.003) | 0.9099 (0.003) |
| 0, 0 | 50 | 0.8964 (0.003) | 0.9092 (0.003) | 0.8964 (0.003) | 0.9147 (0.003) |
| 0, 0 | 60 | 0.8959 (0.003) | 0.9041 (0.003) | 0.8959 (0.003) | 0.9180 (0.003) |
| 0, 0 | 70 | 0.9007 (0.003) | 0.9046 (0.003) | 0.9007 (0.003) | 0.9238 (0.003) |
| 0, 0 | 80 | 0.8952 (0.003) | 0.8973 (0.003) | 0.8952 (0.003) | 0.9152 (0.003) |
| 0, 0 | 90 | 0.9012 (0.003) | 0.9025 (0.003) | 0.9012 (0.003) | 0.9189 (0.003) |
| 0, 0 | 100 | 0.9018 (0.003) | 0.9020 (0.003) | 0.9018 (0.003) | 0.9134 (0.003) |
| 0, 0 | 200 | 0.9025 (0.003) | 0.9025 (0.003) | 0.9025 (0.003) | 0.9194 (0.003) |
| 0, 0 | 507 | 0.9005 (0.003) | 0.9008 (0.003) | 0.9005 (0.003) | 0.9162 (0.003) |
| 0.15, 0.15 | 20 | 0.9256 (0.003) | 0.9304 (0.003) | 0.9122 (0.003) | 0.9428 (0.002) |
| 0.15, 0.15 | 30 | 0.9020 (0.003) | 0.9119 (0.003) | 0.8929 (0.003) | 0.9263 (0.003) |
| 0.15, 0.15 | 40 | 0.8972 (0.003) | 0.9068 (0.003) | 0.8983 (0.003) | 0.9256 (0.003) |
| 0.15, 0.15 | 50 | 0.8987 (0.003) | 0.9030 (0.003) | 0.9044 (0.003) | 0.9228 (0.003) |
| 0.15, 0.15 | 60 | 0.8976 (0.003) | 0.9030 (0.003) | 0.8978 (0.003) | 0.9206 (0.003) |
| 0.15, 0.15 | 70 | 0.8990 (0.003) | 0.9046 (0.003) | 0.8927 (0.003) | 0.9211 (0.003) |
| 0.15, 0.15 | 80 | 0.8939 (0.003) | 0.9000 (0.003) | 0.8925 (0.003) | 0.9151 (0.003) |
| 0.15, 0.15 | 90 | 0.8997 (0.003) | 0.9056 (0.003) | 0.8968 (0.003) | 0.9184 (0.003) |
| 0.15, 0.15 | 100 | 0.8968 (0.003) | 0.9056 (0.003) | 0.8928 (0.003) | 0.9139 (0.003) |
| 0.15, 0.15 | 200 | 0.9016 (0.003) | 0.9046 (0.003) | 0.9048 (0.003) | 0.9161 (0.003) |
| 0.15, 0.15 | 507 | 0.9040 (0.003) | 0.9033 (0.003) | 0.8986 (0.003) | 0.9156 (0.003) |
| 0.25, 0.25 | 20 | 0.9428 (0.002) | 0.9433 (0.002) | 0.9358 (0.002) | 0.9598 (0.002) |
| 0.25, 0.25 | 30 | 0.9108 (0.003) | 0.9189 (0.003) | 0.9074 (0.003) | 0.9442 (0.002) |
| 0.25, 0.25 | 40 | 0.9007 (0.003) | 0.9134 (0.003) | 0.9014 (0.003) | 0.9364 (0.002) |
| 0.25, 0.25 | 50 | 0.8921 (0.003) | 0.9021 (0.003) | 0.8958 (0.003) | 0.9269 (0.003) |
| 0.25, 0.25 | 60 | 0.8939 (0.003) | 0.9021 (0.003) | 0.8991 (0.003) | 0.9224 (0.003) |
| 0.25, 0.25 | 70 | 0.9000 (0.003) | 0.9098 (0.003) | 0.9004 (0.003) | 0.9239 (0.003) |
| 0.25, 0.25 | 80 | 0.8959 (0.003) | 0.9024 (0.003) | 0.8983 (0.003) | 0.9167 (0.003) |
| 0.25, 0.25 | 90 | 0.9004 (0.003) | 0.9072 (0.003) | 0.8983 (0.003) | 0.9176 (0.003) |
| 0.25, 0.25 | 100 | 0.9010 (0.003) | 0.9054 (0.003) | 0.8962 (0.003) | 0.9193 (0.003) |
| 0.25, 0.25 | 200 | 0.8988 (0.003) | 0.9044 (0.003) | 0.8977 (0.003) | 0.9131 (0.003) |
| 0.25, 0.25 | 507 | 0.9037 (0.003) | 0.9045 (0.003) | 0.9028 (0.003) | 0.9116 (0.003) |
| 0.1, 0.3 | 20 | 0.9278 (0.003) | 0.9059 (0.003) | 0.9173 (0.003) | 0.9467 (0.002) |
| 0.1, 0.3 | 30 | 0.9024 (0.003) | 0.8781 (0.003) | 0.8998 (0.003) | 0.9325 (0.003) |
| 0.1, 0.3 | 40 | 0.8968 (0.003) | 0.8684 (0.003) | 0.8980 (0.003) | 0.9275 (0.003) |
| 0.1, 0.3 | 50 | 0.9010 (0.003) | 0.8680 (0.003) | 0.9011 (0.003) | 0.9276 (0.003) |
| 0.1, 0.3 | 60 | 0.8971 (0.003) | 0.8693 (0.003) | 0.8981 (0.003) | 0.9194 (0.003) |
| 0.1, 0.3 | 70 | 0.8964 (0.003) | 0.8640 (0.003) | 0.8982 (0.003) | 0.9181 (0.003) |
| 0.1, 0.3 | 80 | 0.8963 (0.003) | 0.8662 (0.003) | 0.8954 (0.003) | 0.9153 (0.003) |
| 0.1, 0.3 | 90 | 0.8932 (0.003) | 0.8666 (0.003) | 0.8970 (0.003) | 0.9181 (0.003) |
| 0.1, 0.3 | 100 | 0.8964 (0.003) | 0.8652 (0.003) | 0.8965 (0.003) | 0.9126 (0.003) |
| 0.1, 0.3 | 200 | 0.8958 (0.003) | 0.8856 (0.003) | 0.9021 (0.003) | 0.9087 (0.003) |
| 0.1, 0.3 | 507 | 0.9006 (0.003) | 0.9395 (0.002) | 0.9035 (0.003) | 0.9146 (0.003) |

Table 24: Coverage for the scenarios with non-inferiority margin$\delta=0.1$, true risk difference $\theta=0$ and within-patient correlation $\rho=0.5$; Monte-Carlo standard error in parenthesis

| Probability of missingness per group | Cases | GEE | Hybrid CI | Nam/Tango | Nam/Tango with multiple imputation |
| --- | --- | --- | --- | --- | --- |
| 0, 0 | 20 | 0.8960 (0.003) | 0.9178 (0.003) | 0.9062 (0.003) | 0.9324 (0.003) |
| 0, 0 | 30 | 0.8996 (0.003) | 0.9130 (0.003) | 0.9012 (0.003) | 0.9214 (0.003) |
| 0, 0 | 40 | 0.9041 (0.003) | 0.9145 (0.003) | 0.9041 (0.003) | 0.9240 (0.003) |
| 0, 0 | 50 | 0.9009 (0.003) | 0.9139 (0.003) | 0.9009 (0.003) | 0.9300 (0.003) |
| 0, 0 | 60 | 0.8994 (0.003) | 0.9116 (0.003) | 0.8994 (0.003) | 0.9272 (0.003) |
| 0, 0 | 70 | 0.8959 (0.003) | 0.9061 (0.003) | 0.8959 (0.003) | 0.9307 (0.003) |
| 0, 0 | 80 | 0.8952 (0.003) | 0.9015 (0.003) | 0.8952 (0.003) | 0.9237 (0.003) |
| 0, 0 | 90 | 0.9016 (0.003) | 0.9060 (0.003) | 0.9016 (0.003) | 0.9298 (0.003) |
| 0, 0 | 100 | 0.8993 (0.003) | 0.9009 (0.003) | 0.8993 (0.003) | 0.9252 (0.003) |
| 0, 0 | 200 | 0.9007 (0.003) | 0.9007 (0.003) | 0.9007 (0.003) | 0.9263 (0.003) |
| 0, 0 | 507 | 0.8981 (0.003) | 0.8997 (0.003) | 0.8981 (0.003) | 0.9272 (0.003) |
| 0.15, 0.15 | 20 | 0.9355 (0.002) | 0.9380 (0.002) | 0.9236 (0.003) | 0.9471 (0.002) |
| 0.15, 0.15 | 30 | 0.9069 (0.003) | 0.9152 (0.003) | 0.8936 (0.003) | 0.9408 (0.002) |
| 0.15, 0.15 | 40 | 0.8980 (0.003) | 0.9074 (0.003) | 0.8958 (0.003) | 0.9325 (0.003) |
| 0.15, 0.15 | 50 | 0.8979 (0.003) | 0.9071 (0.003) | 0.8979 (0.003) | 0.9302 (0.003) |
| 0.15, 0.15 | 60 | 0.8934 (0.003) | 0.9016 (0.003) | 0.8945 (0.003) | 0.9266 (0.003) |
| 0.15, 0.15 | 70 | 0.8979 (0.003) | 0.9082 (0.003) | 0.8971 (0.003) | 0.9300 (0.003) |
| 0.15, 0.15 | 80 | 0.8990 (0.003) | 0.9080 (0.003) | 0.8965 (0.003) | 0.9264 (0.003) |
| 0.15, 0.15 | 90 | 0.9037 (0.003) | 0.9100 (0.003) | 0.8990 (0.003) | 0.9301 (0.003) |
| 0.15, 0.15 | 100 | 0.8962 (0.003) | 0.9045 (0.003) | 0.8956 (0.003) | 0.9235 (0.003) |
| 0.15, 0.15 | 200 | 0.9011 (0.003) | 0.9079 (0.003) | 0.9067 (0.003) | 0.9235 (0.003) |
| 0.15, 0.15 | 507 | 0.9007 (0.003) | 0.9084 (0.003) | 0.8973 (0.003) | 0.9231 (0.003) |
| 0.25, 0.25 | 20 | 0.9477 (0.002) | 0.9471 (0.002) | 0.9540 (0.002) | 0.9659 (0.002) |
| 0.25, 0.25 | 30 | 0.9235 (0.003) | 0.9253 (0.003) | 0.9171 (0.003) | 0.9535 (0.002) |
| 0.25, 0.25 | 40 | 0.9029 (0.003) | 0.9113 (0.003) | 0.8984 (0.003) | 0.9435 (0.002) |
| 0.25, 0.25 | 50 | 0.9012 (0.003) | 0.9115 (0.003) | 0.8938 (0.003) | 0.9407 (0.002) |
| 0.25, 0.25 | 60 | 0.9002 (0.003) | 0.9078 (0.003) | 0.8992 (0.003) | 0.9365 (0.002) |
| 0.25, 0.25 | 70 | 0.8989 (0.003) | 0.9092 (0.003) | 0.9005 (0.003) | 0.9349 (0.002) |
| 0.25, 0.25 | 80 | 0.8978 (0.003) | 0.9086 (0.003) | 0.9019 (0.003) | 0.9306 (0.003) |
| 0.25, 0.25 | 90 | 0.8952 (0.003) | 0.9030 (0.003) | 0.9033 (0.003) | 0.9251 (0.003) |
| 0.25, 0.25 | 100 | 0.8998 (0.003) | 0.9090 (0.003) | 0.8988 (0.003) | 0.9268 (0.003) |
| 0.25, 0.25 | 200 | 0.9024 (0.003) | 0.9097 (0.003) | 0.9032 (0.003) | 0.9205 (0.003) |
| 0.25, 0.25 | 507 | 0.8991 (0.003) | 0.9035 (0.003) | 0.9015 (0.003) | 0.9162 (0.003) |
| 0.1, 0.3 | 20 | 0.9432 (0.002) | 0.9171 (0.003) | 0.9373 (0.002) | 0.9580 (0.002) |
| 0.1, 0.3 | 30 | 0.9116 (0.003) | 0.8843 (0.003) | 0.9123 (0.003) | 0.9429 (0.002) |
| 0.1, 0.3 | 40 | 0.8960 (0.003) | 0.8685 (0.003) | 0.8924 (0.003) | 0.9383 (0.002) |
| 0.1, 0.3 | 50 | 0.8932 (0.003) | 0.8624 (0.003) | 0.8961 (0.003) | 0.9350 (0.002) |
| 0.1, 0.3 | 60 | 0.8979 (0.003) | 0.8756 (0.003) | 0.9008 (0.003) | 0.9302 (0.003) |
| 0.1, 0.3 | 70 | 0.9010 (0.003) | 0.8765 (0.003) | 0.9045 (0.003) | 0.9307 (0.003) |
| 0.1, 0.3 | 80 | 0.9010 (0.003) | 0.8784 (0.003) | 0.9041 (0.003) | 0.9279 (0.003) |
| 0.1, 0.3 | 90 | 0.8979 (0.003) | 0.8782 (0.003) | 0.9010 (0.003) | 0.9263 (0.003) |
| 0.1, 0.3 | 100 | 0.8915 (0.003) | 0.8704 (0.003) | 0.8888 (0.003) | 0.9197 (0.003) |
| 0.1, 0.3 | 200 | 0.8995 (0.003) | 0.9055 (0.003) | 0.8996 (0.003) | 0.9218 (0.003) |
| 0.1, 0.3 | 507 | 0.9045 (0.003) | 0.9574 (0.002) | 0.9035 (0.003) | 0.9219 (0.003) |

Table 25: Interval width for the scenarios with non-inferiority margin $\delta=0.1$, true risk difference $\theta=0$ and within-patient correlation $\rho=0$

| Probability of missingness per group | Cases | GEE | Hybrid CI | Nam/Tango | Nam/Tango with multiple imputation |
| --- | --- | --- | --- | --- | --- |
| 0, 0 | 20 | 0.4368 | 0.4291 | 0.4259 | 0.4195 |
| 0, 0 | 30 | 0.3493 | 0.3475 | 0.3454 | 0.3426 |
| 0, 0 | 40 | 0.2999 | 0.2995 | 0.2980 | 0.2971 |
| 0, 0 | 50 | 0.2677 | 0.2676 | 0.2666 | 0.2665 |
| 0, 0 | 60 | 0.2432 | 0.2434 | 0.2426 | 0.2429 |
| 0, 0 | 70 | 0.2246 | 0.2248 | 0.2242 | 0.2249 |
| 0, 0 | 80 | 0.2101 | 0.2103 | 0.2098 | 0.2108 |
| 0, 0 | 90 | 0.1978 | 0.1980 | 0.1975 | 0.1987 |
| 0, 0 | 100 | 0.1876 | 0.1877 | 0.1874 | 0.1886 |
| 0, 0 | 200 | 0.1320 | 0.1321 | 0.1320 | 0.1334 |
| 0, 0 | 507 | 0.0828 | 0.0828 | 0.0828 | 0.0839 |
| 0.15, 0.15 | 20 | 0.4718 | 0.4701 | 0.5146 | 0.4647 |
| 0.15, 0.15 | 30 | 0.3766 | 0.3809 | 0.4157 | 0.3773 |
| 0.15, 0.15 | 40 | 0.3236 | 0.3280 | 0.3583 | 0.3255 |
| 0.15, 0.15 | 50 | 0.2886 | 0.2928 | 0.3195 | 0.2913 |
| 0.15, 0.15 | 60 | 0.2634 | 0.2664 | 0.2920 | 0.2664 |
| 0.15, 0.15 | 70 | 0.2435 | 0.2464 | 0.2696 | 0.2466 |
| 0.15, 0.15 | 80 | 0.2270 | 0.2293 | 0.2513 | 0.2302 |
| 0.15, 0.15 | 90 | 0.2143 | 0.2166 | 0.2373 | 0.2173 |
| 0.15, 0.15 | 100 | 0.2032 | 0.2050 | 0.2250 | 0.2062 |
| 0.15, 0.15 | 200 | 0.1432 | 0.1442 | 0.1582 | 0.1456 |
| 0.15, 0.15 | 507 | 0.0897 | 0.0902 | 0.0990 | 0.0914 |
| 0.25, 0.25 | 20 | 0.5056 | 0.5027 | 0.6188 | 0.5031 |
| 0.25, 0.25 | 30 | 0.3997 | 0.4080 | 0.4968 | 0.4055 |
| 0.25, 0.25 | 40 | 0.3447 | 0.3532 | 0.4285 | 0.3503 |
| 0.25, 0.25 | 50 | 0.3069 | 0.3142 | 0.3813 | 0.3128 |
| 0.25, 0.25 | 60 | 0.2795 | 0.2863 | 0.3474 | 0.2854 |
| 0.25, 0.25 | 70 | 0.2585 | 0.2636 | 0.3206 | 0.2638 |
| 0.25, 0.25 | 80 | 0.2414 | 0.2462 | 0.2994 | 0.2466 |
| 0.25, 0.25 | 90 | 0.2273 | 0.2318 | 0.2813 | 0.2324 |
| 0.25, 0.25 | 100 | 0.2160 | 0.2201 | 0.2674 | 0.2208 |
| 0.25, 0.25 | 200 | 0.1523 | 0.1544 | 0.1877 | 0.1556 |
| 0.25, 0.25 | 507 | 0.0955 | 0.0965 | 0.1174 | 0.0976 |
| 0.1, 0.3 | 20 | 0.4933 | 0.4217 | 0.5612 | 0.4876 |
| 0.1, 0.3 | 30 | 0.3905 | 0.3389 | 0.4514 | 0.3936 |
| 0.1, 0.3 | 40 | 0.3356 | 0.2918 | 0.3883 | 0.3403 |
| 0.1, 0.3 | 50 | 0.2995 | 0.2606 | 0.3467 | 0.3041 |
| 0.1, 0.3 | 60 | 0.2726 | 0.2377 | 0.3152 | 0.2771 |
| 0.1, 0.3 | 70 | 0.2524 | 0.2211 | 0.2917 | 0.2569 |
| 0.1, 0.3 | 80 | 0.2362 | 0.2079 | 0.2731 | 0.2407 |
| 0.1, 0.3 | 90 | 0.2226 | 0.1971 | 0.2571 | 0.2269 |
| 0.1, 0.3 | 100 | 0.2106 | 0.1877 | 0.2431 | 0.2147 |
| 0.1, 0.3 | 200 | 0.1486 | 0.1422 | 0.1711 | 0.1515 |
| 0.1, 0.3 | 507 | 0.0932 | 0.1097 | 0.1070 | 0.0953 |

Table 26: Interval width for the scenarios with non-inferiority margin $\delta=0.1$, true risk difference $\theta=0$ and within-patient correlation $\rho=0.37$

| Probability of missingness per group | Cases | GEE | Hybrid CI | Nam/Tango | Nam/Tango with multiple imputation |
| --- | --- | --- | --- | --- | --- |
| 0, 0 | 20 | 0.3411 | 0.3480 | 0.3634 | 0.3435 |
| 0, 0 | 30 | 0.2745 | 0.2802 | 0.2889 | 0.2815 |
| 0, 0 | 40 | 0.2359 | 0.2406 | 0.2464 | 0.2442 |
| 0, 0 | 50 | 0.2106 | 0.2141 | 0.2187 | 0.2191 |
| 0, 0 | 60 | 0.1923 | 0.1951 | 0.1986 | 0.2005 |
| 0, 0 | 70 | 0.1775 | 0.1798 | 0.1827 | 0.1854 |
| 0, 0 | 80 | 0.1661 | 0.1680 | 0.1705 | 0.1739 |
| 0, 0 | 90 | 0.1560 | 0.1577 | 0.1598 | 0.1636 |
| 0, 0 | 100 | 0.1482 | 0.1496 | 0.1514 | 0.1555 |
| 0, 0 | 200 | 0.1045 | 0.1050 | 0.1057 | 0.1101 |
| 0, 0 | 507 | 0.0655 | 0.0656 | 0.0658 | 0.0692 |
| 0.15, 0.15 | 20 | 0.3917 | 0.4026 | 0.4539 | 0.4034 |
| 0.15, 0.15 | 30 | 0.3077 | 0.3217 | 0.3552 | 0.3224 |
| 0.15, 0.15 | 40 | 0.2644 | 0.2760 | 0.3013 | 0.2784 |
| 0.15, 0.15 | 50 | 0.2353 | 0.2453 | 0.2661 | 0.2478 |
| 0.15, 0.15 | 60 | 0.2145 | 0.2229 | 0.2408 | 0.2265 |
| 0.15, 0.15 | 70 | 0.1987 | 0.2063 | 0.2218 | 0.2091 |
| 0.15, 0.15 | 80 | 0.1856 | 0.1922 | 0.2061 | 0.1958 |
| 0.15, 0.15 | 90 | 0.1749 | 0.1811 | 0.1936 | 0.1843 |
| 0.15, 0.15 | 100 | 0.1659 | 0.1719 | 0.1830 | 0.1750 |
| 0.15, 0.15 | 200 | 0.1171 | 0.1206 | 0.1271 | 0.1235 |
| 0.15, 0.15 | 507 | 0.0735 | 0.0753 | 0.0789 | 0.0776 |
| 0.25, 0.25 | 20 | 0.4440 | 0.4471 | 0.5599 | 0.4540 |
| 0.25, 0.25 | 30 | 0.3419 | 0.3560 | 0.4355 | 0.3593 |
| 0.25, 0.25 | 40 | 0.2902 | 0.3055 | 0.3667 | 0.3071 |
| 0.25, 0.25 | 50 | 0.2588 | 0.2727 | 0.3226 | 0.2735 |
| 0.25, 0.25 | 60 | 0.2351 | 0.2481 | 0.2902 | 0.2490 |
| 0.25, 0.25 | 70 | 0.2181 | 0.2295 | 0.2671 | 0.2306 |
| 0.25, 0.25 | 80 | 0.2036 | 0.2136 | 0.2477 | 0.2149 |
| 0.25, 0.25 | 90 | 0.1916 | 0.2009 | 0.2320 | 0.2023 |
| 0.25, 0.25 | 100 | 0.1821 | 0.1907 | 0.2195 | 0.1923 |
| 0.25, 0.25 | 200 | 0.1288 | 0.1343 | 0.1517 | 0.1356 |
| 0.25, 0.25 | 507 | 0.0807 | 0.0838 | 0.0936 | 0.0849 |
| 0.1, 0.3 | 20 | 0.4181 | 0.3639 | 0.5002 | 0.4302 |
| 0.1, 0.3 | 30 | 0.3246 | 0.2883 | 0.3902 | 0.3412 |
| 0.1, 0.3 | 40 | 0.2772 | 0.2474 | 0.3293 | 0.2931 |
| 0.1, 0.3 | 50 | 0.2473 | 0.2218 | 0.2900 | 0.2613 |
| 0.1, 0.3 | 60 | 0.2256 | 0.2035 | 0.2623 | 0.2388 |
| 0.1, 0.3 | 70 | 0.2085 | 0.1896 | 0.2409 | 0.2203 |
| 0.1, 0.3 | 80 | 0.1946 | 0.1783 | 0.2236 | 0.2056 |
| 0.1, 0.3 | 90 | 0.1843 | 0.1706 | 0.2107 | 0.1945 |
| 0.1, 0.3 | 100 | 0.1745 | 0.1630 | 0.1988 | 0.1837 |
| 0.1, 0.3 | 200 | 0.1233 | 0.1282 | 0.1377 | 0.1300 |
| 0.1, 0.3 | 507 | 0.0774 | 0.1045 | 0.0853 | 0.0816 |

Table 27: Interval width for the scenarios with non-inferiority margin$\delta=0.1$, true risk difference $\theta=0$ and within-patient correlation $\rho=0.5$

| Probability of missingness per group | Cases | GEE | Hybrid CI | Nam/Tango | Nam/Tango with multiple imputation |
| --- | --- | --- | --- | --- | --- |
| 0, 0 | 20 | 0.3012 | 0.3159 | 0.3400 | 0.3175 |
| 0, 0 | 30 | 0.2429 | 0.2533 | 0.2667 | 0.2598 |
| 0, 0 | 40 | 0.2104 | 0.2176 | 0.2267 | 0.2260 |
| 0, 0 | 50 | 0.1884 | 0.1938 | 0.2006 | 0.2030 |
| 0, 0 | 60 | 0.1712 | 0.1754 | 0.1809 | 0.1850 |
| 0, 0 | 70 | 0.1583 | 0.1617 | 0.1662 | 0.1715 |
| 0, 0 | 80 | 0.1482 | 0.1510 | 0.1548 | 0.1607 |
| 0, 0 | 90 | 0.1392 | 0.1417 | 0.1449 | 0.1513 |
| 0, 0 | 100 | 0.1324 | 0.1345 | 0.1373 | 0.1439 |
| 0, 0 | 200 | 0.0934 | 0.0941 | 0.0952 | 0.1018 |
| 0, 0 | 507 | 0.0586 | 0.0588 | 0.0590 | 0.0640 |
| 0.15, 0.15 | 20 | 0.3619 | 0.3785 | 0.4340 | 0.3822 |
| 0.15, 0.15 | 30 | 0.2796 | 0.2992 | 0.3341 | 0.3029 |
| 0.15, 0.15 | 40 | 0.2387 | 0.2550 | 0.2802 | 0.2603 |
| 0.15, 0.15 | 50 | 0.2120 | 0.2259 | 0.2451 | 0.2316 |
| 0.15, 0.15 | 60 | 0.1935 | 0.2054 | 0.2211 | 0.2112 |
| 0.15, 0.15 | 70 | 0.1792 | 0.1899 | 0.2029 | 0.1951 |
| 0.15, 0.15 | 80 | 0.1668 | 0.1761 | 0.1876 | 0.1816 |
| 0.15, 0.15 | 90 | 0.1582 | 0.1670 | 0.1766 | 0.1719 |
| 0.15, 0.15 | 100 | 0.1498 | 0.1579 | 0.1665 | 0.1627 |
| 0.15, 0.15 | 200 | 0.1058 | 0.1108 | 0.1147 | 0.1147 |
| 0.15, 0.15 | 507 | 0.0665 | 0.0693 | 0.0709 | 0.0721 |
| 0.25, 0.25 | 20 | 417.193 | 0.4288 | 0.5422 | 0.4388 |
| 0.25, 0.25 | 30 | 0.3188 | 0.3400 | 0.4147 | 0.3433 |
| 0.25, 0.25 | 40 | 0.2690 | 0.2892 | 0.3457 | 0.2924 |
| 0.25, 0.25 | 50 | 0.2377 | 0.2556 | 0.3010 | 0.2595 |
| 0.25, 0.25 | 60 | 0.2161 | 0.2322 | 0.2699 | 0.2356 |
| 0.25, 0.25 | 70 | 0.1994 | 0.2140 | 0.2462 | 0.2173 |
| 0.25, 0.25 | 80 | 0.1858 | 0.1996 | 0.2273 | 0.2023 |
| 0.25, 0.25 | 90 | 0.1758 | 0.1885 | 0.2135 | 0.1907 |
| 0.25, 0.25 | 100 | 0.1668 | 0.1786 | 0.2011 | 0.1806 |
| 0.25, 0.25 | 200 | 0.1177 | 0.1254 | 0.1373 | 0.1269 |
| 0.25, 0.25 | 507 | 0.0740 | 0.0784 | 0.0843 | 0.0797 |
| 0.1, 0.3 | 20 | 0.3916 | 0.3450 | 0.4816 | 0.4116 |
| 0.1, 0.3 | 30 | 0.2994 | 0.2703 | 0.3702 | 0.3248 |
| 0.1, 0.3 | 40 | 0.2520 | 0.2292 | 0.3069 | 0.2762 |
| 0.1, 0.3 | 50 | 0.2249 | 0.2060 | 0.2692 | 0.2461 |
| 0.1, 0.3 | 60 | 0.2052 | 0.1891 | 0.2427 | 0.2238 |
| 0.1, 0.3 | 70 | 0.1895 | 0.1768 | 0.2216 | 0.2069 |
| 0.1, 0.3 | 80 | 0.1770 | 0.1667 | 0.2053 | 0.1927 |
| 0.1, 0.3 | 90 | 0.1666 | 0.1589 | 0.1920 | 0.1813 |
| 0.1, 0.3 | 100 | 0.1585 | 0.1528 | 0.1816 | 0.1719 |
| 0.1, 0.3 | 200 | 0.1120 | 0.1224 | 0.1247 | 0.1211 |
| 0.1, 0.3 | 507 | 0.0704 | 0.1019 | 0.0767 | 0.0759 |
